# New Algorithm for Managing Childhood Illness Using Mobile Technology (ALMANACH): A Controlled Non-Inferiority Study on Clinical Outcome and Antibiotic Use in Tanzania

**DOI:** 10.1371/journal.pone.0132316

**Published:** 2015-07-10

**Authors:** Amani Flexson Shao, Clotilde Rambaud-Althaus, Josephine Samaka, Allen Festo Faustine, Seneca Perri-Moore, Ndeniria Swai, Judith Kahama-Maro, Marc Mitchell, Blaise Genton, Valérie D’Acremont

**Affiliations:** 1 Swiss Tropical and Public Health Institute, Basel, Switzerland; 2 University of Basel, Basel, Switzerland; 3 National Institute for Medical Research, Tukuyu Medical Research Center, Tukuyu, Tanzania; 4 University of Utah, Salt Lake City, Utah, United States of America; 5 Harvard School of Public Health, Boston, Massachusetts, United States of America; 6 City Medical Office of Health, Dar es Salaam City Council, Dar es Salaam, Tanzania; 7 Department of Ambulatory Care and Community Medicine, University Hospital, Lausanne, Switzerland; 8 Infectious Diseases Service, University Hospital, Lausanne, Switzerland; 9 Amana Hospital, Dar es Salaam, Tanzania; 10 Kilombero District Council, PO Box 263, Morogoro, Tanzania; Kenya Medical Research Institute—Wellcome Trust Research Programme, KENYA

## Abstract

**Introduction:**

The decline of malaria and scale-up of rapid diagnostic tests calls for a revision of IMCI. A new algorithm (ALMANACH) running on mobile technology was developed based on the latest evidence. The objective was to ensure that ALMANACH was safe, while keeping a low rate of antibiotic prescription.

**Methods:**

Consecutive children aged 2–59 months with acute illness were managed using ALMANACH (2 intervention facilities), or standard practice (2 control facilities) in Tanzania. Primary outcomes were proportion of children cured at day 7 and who received antibiotics on day 0.

**Results:**

130/842 (15∙4%) in ALMANACH and 241/623 (38∙7%) in control arm were diagnosed with an infection in need for antibiotic, while 3∙8% and 9∙6% had malaria. 815/838 (97∙3%;96∙1–98.4%) were cured at D7 using ALMANACH versus 573/623 (92∙0%;89∙8–94∙1%) using standard practice (p<0∙001). Of 23 children not cured at D7 using ALMANACH, 44% had skin problems, 30% pneumonia, 26% upper respiratory infection and 13% likely viral infection at D0. Secondary hospitalization occurred for one child using ALMANACH and one who eventually died using standard practice. At D0, antibiotics were prescribed to 15∙4% (12∙9–17∙9%) using ALMANACH versus 84∙3% (81∙4–87∙1%) using standard practice (p<0∙001). 2∙3% (1∙3–3.3) versus 3∙2% (1∙8–4∙6%) received an antibiotic secondarily.

**Conclusion:**

Management of children using ALMANACH improve clinical outcome and reduce antibiotic prescription by 80%. This was achieved through more accurate diagnoses and hence better identification of children in need of antibiotic treatment or not. The building on mobile technology allows easy access and rapid update of the decision chart.

**Trial Registration:**

Pan African Clinical Trials Registry PACTR201011000262218

## Introduction

About 7 million children under 5 years of age die each year despite the availability of effective low-cost interventions [1]. The Integrated Management of Childhood Illness (IMCI) strategy developed by the World Health Organization (WHO), UNICEF and other partners in mid 1990s [2] could potentially prevent two-thirds of these deaths [3]. To date, IMCI is still a good tool and studies that assessed its impact showed borderline reduction of childhood mortality [4]. When health workers were trained to use IMCI, their performance in case management improved [5, 6], although cautious interpretation is needed due to heterogeneities in methodologies of assessment [6]. Worldwide, the impact of IMCI has been less than expected due to health system challenges, such as shortage of health workers [7–9], poor motivation and lack of supervision [10, 11]. All this leads to low compliance to the IMCI guidelines [12–14] and probably poorer health outcomes than it could be.

IMCI is facing additional challenges. First, a precise evaluation of the clinical outcome of children when managed with the IMCI algorithm is lacking, which leaves a doubt about its real benefit. Some studies evaluated the clinical outcome of children with specific diseases or conditions, such as severe pneumonia at peripheral health facilities [15–17] or malaria and pneumonia at community level [18, 19]. These studies also demonstrated that effectively trained and supervised community health workers using malaria rapid diagnostic tests (mRDTs) with [18], or without [19] respiratory rate (RR) timers could adequately classify and treat children less than 5 years with malaria and/or pneumonia at community level. The overuse of antimalarials was limited, but varying degrees of antibiotics over-prescription were observed [18]. Secondly, patterns of disease and drug resistance have evolved dramatically in the last 20 years. The prevalence of malaria has considerably declined in the last decade across different settings [20]. While mRDT has just been incorporated in the new IMCI version [21], many national IMCI guidelines still recommend to treat presumptively all febrile children with antimalarials. Third, since the advent of mRDT, the proportion of patients receiving antibiotics has increased [22–24], probably because clinicians have not enough guidance on how to proceed when mRDTs results are negative. The new IMCI guidelines do not include precise guidance on typhoid fever, urinary tract infections (UTI) or other causes of unspecific fever. Even if these conditions might have limited impact on mortality, they are feared by primary health care clinicians who often prescribe antibiotics to prevent potential complications.

Based on the IMCI algorithm, a review of the literature, and the results of an etiology of fever study conducted in Tanzania [25], a novel ALgorithm for the MANAgement of CHildhood illness (ALMANACH) was developed (Rambaud Althaus et al, submitted) (S1 File). This algorithm was primarily aimed at decreasing unnecessary prescription of antibiotics in children, while ensuring same or even better clinical outcome compared to routine practice (non inferiority trial).The objective of the present study was to measure the impact of its use on clinical outcome and antibiotic prescription in children attending primary care facilities in rural and urban settings of Tanzania.

## Materials and Methods

### Study sites and subjects

The study was conducted as part of a larger project which aimed at improving the quality of care and rational use of medicines for children in Tanzania (PeDiAtrick project, registration number PACTR201011000262218 at www.pactr.org) (S2 File). For the present study, two pairs (one from urban Dar es Salaam and one from the rural Morogoro region) of two nearby primary care health facilities (HF), similar in terms of natural environment, malaria prevalence, socio-economic status of the catchment population, and type of services available, were conveniently selected. Then, in Dar es Salaam, Ilala municipality (city center), Buguruni was randomly selected as intervention and Vingunguti as control HF; in Morogoro region, Kilombero district, Signal was selected as intervention and Mangula as control. We chose to conduct the study in different health facilities rather than to use a parallel design or recruit consecutively patients in the same health facility because the latter increased the risk of including patients with different disease frequency between the intervention (ALMANACH) and routine practice arms due to seasonal variation. There was also the risk of biased results due to contamination between arms because clinicians would have gained a better understanding of disease classification or change their behaviour in terms of antimicrobial prescription because of the ALMANACH training and use. Consecutive children aged 2 to 59 months were enrolled by trained study nurse if they fulfilled the inclusion criteria: 1) first consultation for the current illness; 2) absence of severe illness requiring immediate life-saving procedures; 3) main complaint(s) not related to injury or trauma; 4) living in the catchment area of the HF and; 5) written informed consent by the caretaker.

### Study design and procedures

A controlled non-inferiority trial (S3 File) was conducted to compare the clinical outcome of children managed according to ALMANACH or to standard practice. Children enrolled in the intervention arm were managed by two study clinicians (one for each setting) who were trained to strictly comply with the ALMANACH algorithm, which was available on paper at the start of the study and used in the first 100 patients, and then built in an electronic support (smartphone running Open MRS) and used for the remaining 742 patients (S4 and S5 Files). Both versions were identical. In the control arm, children were attended by the usual HF clinicians, of which about 80% had been trained for IMCI [26]. In general, this training had taken place several years before, and compliance to guidelines was known to be rather poor, with most patients receiving antibiotics, especially when tested negative for malaria [22]. mRDT and commonly prescribed medicines were made available throughout the study period in both arms.

During the one-month pilot phase, study clinicians in the ALMANACH arm received face-to-face supervision with several real patients to check their ability to identify all relevant signs, including RR measurement. In the control arm, no algorithm, training or supervision was performed. Observing study clinician obtained oral consent from the routine clinician and observed the consultation to record key information such as symptoms, signs, laboratory investigation(s) performed, diagnosis(es), advice to caretakers and treatment(s) prescribed. He was instructed not to interfere with the consultation to avoid introducing additional bias to the observer effect.

### Ethics statement

All procedures followed the Good Clinical Practice guidelines. The study protocol and related documents were approved by Ethikkommission beider Basel in Switzerland, by the Institutional Review Board of the Ifakara Health Institute and by the National Institute for Medical Research Review Board in Tanzania (NIMR/HQ/R.8a/Vol.IX/823).

### Content of the ALMANACH algorithm

The development and content of ALMANACH is described in another paper (Rambaud Althaus et al, submitted). In brief, this new algorithm was based on IMCI, but differed on some key features presented in Table 1.

**Table 1:** Key differences between the IMCI and the new ALgorithm for the MANAgement of Childhood illness (ALMANACH) (section dedicated to the management of acute conditions in children aged 2 months to 5 years).

	IMCI algorithm	New algorithm (ALMANACH)
**Danger signs**	5 danger signs managed at the start: unable to drink or breastfeed; lethargic or unconscious; vomits everything; convulsing now or has had convulsions Six additional danger signs assessed later: stridor; chest indrawing; sunken eyes; skin pinch goes back very slowly; stiff neck; tender swelling behind ear	10 danger signs managed at the start: unable to drink or breastfeed; lethargic or unconscious; jaundice; vomits everything; convulsing now or has had convulsions; cyanosis; severe pallor; stiff neck and severe wasting Six additional danger signs assessed later: stridor; chest indrawing;; sunken eyes; skin pinch goes back very slowly; tender swelling behind ear; infected skin lesion or lump larger than 4 cm or with red streaks or with tender nodes or multiple abscesses
**Fever**	1 out of 4 Main symptoms	A dividing point between a febrile branch and a non- febrile branch
**Pneumonia**	Cough + fast breathing^a^	Fever + cough + very fast breathing^b^
**Urinary Tract Infection**	Not considered	Febrile child<2 years with no source identified at this point^c^, and with a positive (leucocytes or nitrites) urine dipstick.
**Typhoid fever**	Not considered	Febrile child ≥2 years with no source identified at this point^c^, and with abdominal tenderness
**Likely viral infection**	Not existing	Febrile child with no classification at the end of the algorithm

^a^ 50 breaths/min for children aged 2 to 12 months, 40 breaths/min for children aged 12 months to 5 years.

^b^ 50 breaths/min for all children (aged 2 months to 5 years).

^c^ No cough or difficult breathing, no diarrhea, no ear problem, no measles, no infected skin lesion or lump.

### Management of children during spontaneous re-attendance

In the intervention arm, caretakers were informed to bring the child back to the study HF if he/she was not able to drink or breastfeed, became sicker, developed fever, fast or difficult breathing, or blood in stool. During working hours, sick children were reassessed by the study clinician, and managed again according to ALMANACH. Out of working hours, children were managed by routine clinicians who were asked to record demographic data, laboratory results, diagnoses, treatments and need for referral in order to hand them back to the study clinician the day after. In the control arm, children were advised on when to come back and managed during re-attendance at the discretion of the routine clinician, who were asked to record the same information on the re-attendances and to hand them back to the study team. In both arms, information regarding visits to other health facilities than the study facilities, and on additional treatment received, was recorded during the follow-up visit at day 7.

### Follow-up of children at day 7 and 14

Caretakers in both arms were asked to bring back their child on day 7 to assess if he/she was cured or not. Children were declared cured if the caretaker reported the child to be well. All children reported as not cured were attended by the clinician and managed again according to ALMANACH in the intervention arm and to usual practice in the control one. When the child had not recovered at day 7, caretakers were asked to return on day 14 for a new assessment. Caretakers whose children did not turn up at day 7 were reminded by phone about the visit and, if not reached, visited at home.

### Data collection, management and analysis

In the intervention arm, a standardized case report form (CRF) was completed during the paper phase of the study. Data collection included demographics, all relevant symptoms and signs, laboratory investigation(s), diagnosis(es), advice and treatment(s) received. During the electronic phase (smartphone), a shorter version of the CRF was used not to repeat data that were automatically sent to the server when running through the decision chart. In the control arm, the observing study clinician filled another CRF that included all relevant information mentioned above. The CRFs were adapted from the health facility survey checklist questionnaire developed by WHO [27].

Beside data sent directly from the smartphone to the server, all information was double-entered in Epi-info software version 3∙5∙3 (CDC Atlanta, USA). Data management and analysis were done using STATA software version 10∙1 (College Station, Texas, USA). The primary outcome measures were: i) proportion of children cured at day 7, and ii) proportion of children who received antibiotics on day 0. Secondary outcome measures were i) proportion of children admitted secondarily or who died, ii) proportion of children who received antibiotics during the whole study period. The above proportions were compared between the intervention and control group using Chi-square test and, when appropriate, Fisher exact test.

To calculate the sample size, we assumed that 95% of children managed with standard practice would be cured on day 7 [28]. To show non-inferiority of the intervention arm with a 3% margin, 80% power and 0.05 level of significance, and using a ratio of 3:2 in order to have more patients in the intervention arm, we calculated that 816 patients in the intervention and 544 in the control arm were needed. Taking into account a 3% rate of loss to follow-up, the target sample size was thus 840 and 560 patients in the intervention and control arms respectively.

## Results

### Status at inclusion

**Fig 1:**
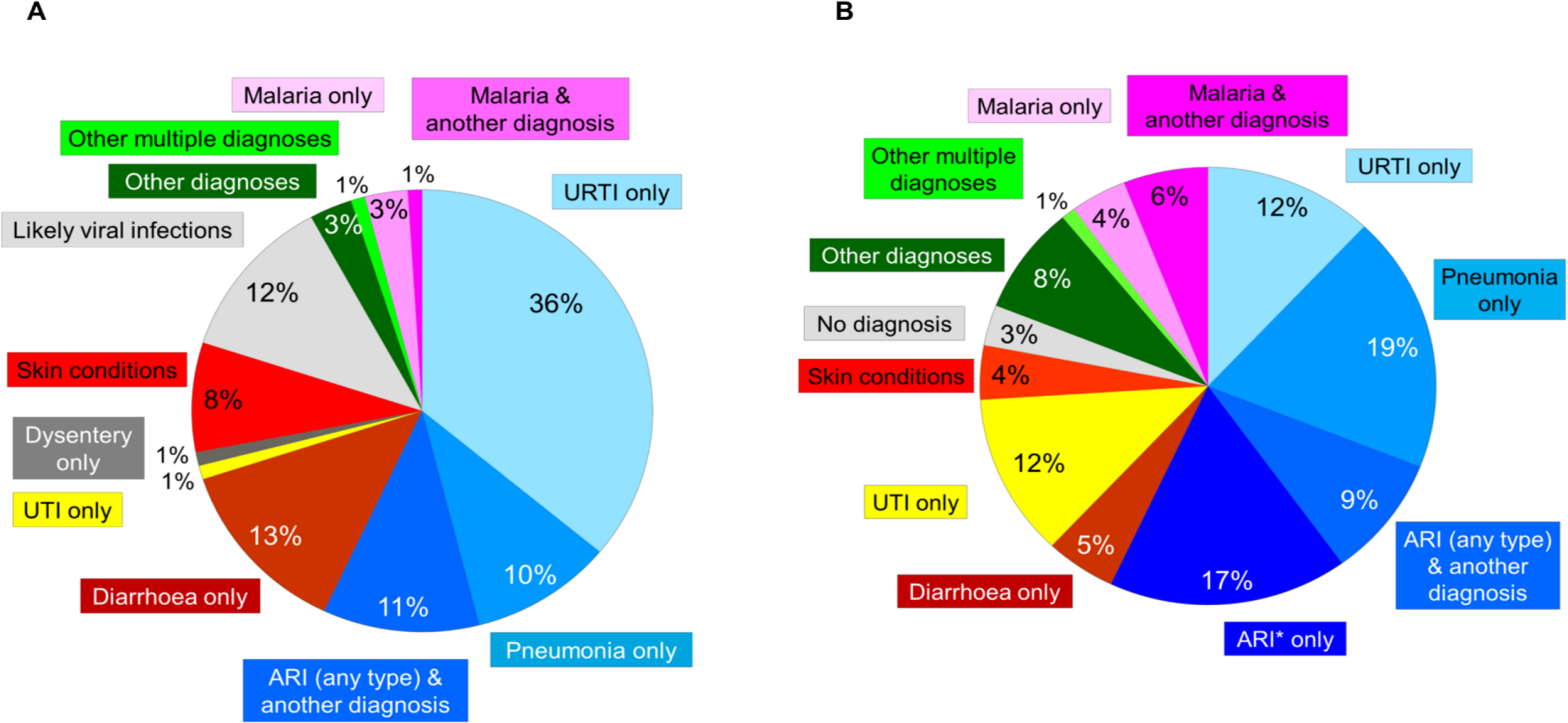
Distribution of diagnoses at inclusion in the ALMANACH (A) and standard practice (B) arms. * Diagnosis given by clinicians when they do not classify further the respiratory infection.

Between December 2010 and June 2011, 1467 children [median age 14 months) were enrolled, 844 (523 in the urban and 321 in the rural setting) in the ALMANACH and 623 (353 in the urban and 270 in the rural setting) in the standard practice arm. Two children were then excluded, one because he was not visiting the health facility for the first time for the current problem, and one who was <2 months of age. Baseline characteristics of patients included are presented in Table 2. The diagnoses distribution in the ALMANACH and standard practice arms are featured in Fig 1. Acute respiratory infections (ARI), either alone or in combination with another condition besides malaria, accounted for 57% and 58% of the diagnoses in intervention and control arms. However, the classification within respiratory infections was quite different between arms: 10∙3% (95%CI 8∙3–12∙4%) were classified as having pneumonia in the ALMANACH arm while 18∙5% (15∙4–21∙5%) as having pneumonia and 17∙0% (14∙1–20∙2%) as having ARI (a diagnosis given by routine clinicians when they did not classify further the respiratory infection but for which they tended to prescribe antibiotics) in the standard practice arm. Only 1∙0% (0∙1–1∙2%) in the ALMANACH versus 12∙0% (9∙3–14∙4%) in the standard practice arm were classified as having UTI. 3∙8% (2∙5–5∙1%) were diagnosed with malaria alone or in combination with another diagnosis in the ALMANACH versus 9∙6% (7∙3–12∙0%) in the standard practice arm, despite full availability of mRDT in all HFs.

**Table 2:** Baseline characteristics of the patients in intervention (ALMANACH) and control (standard practice) arms (n = 1465).

Characteristic	ALMANACH		Standard practice	
	n/N	%	n/N	%
**Gender**				
Females	407/842	48∙3	300/623	48∙2
**Age (in months)**				
2–12	426/842	50∙6	241/623	38∙7
13–24	216/842	25∙7	174/623	27∙9
25–36	106/842	12∙6	89/623	14∙3
37–48	64/842	7∙6	81/623	13∙0
49–59	30/842	3∙6	38/623	6∙1
**Main symptoms**				
Fever	571/842	67∙8	511/623	82∙0
Cough	498/842	59.1	355/623	57∙0
Diarrhoea	184/842	21∙9	76/623	12∙2
Vomiting	57/842	6∙8	78/623	12∙5
Ear problem	14/842	1∙7	13/623	1∙5
**Fast breathing**				
≥50 breaths per minute	100/351	28∙5	42/310	13∙6
**Danger signs**	1/842	0∙1	5/623	0∙8
Lethargic	1/842	0∙1	0/623	0
Vomiting everything	0/842	0	1/623	0∙2
Unable to drink/breastfeed	0/842	0	2/623	0∙3
History of convulsion	0/842	0	2/623	0∙3
**Hospitalization at inclusion**				
Admission on day 0*	3/842	0∙4	21/623	3∙4

*The control health facility of the rural area (Mang’ula Health Center) had a higher number of admissions on day zero because of the possibility to admit patients on site (unlike the intervention rural health facility (Signal Dispensary).

### Clinical outcome

#### Cure rate at day 7 and 14

**Fig 2:**
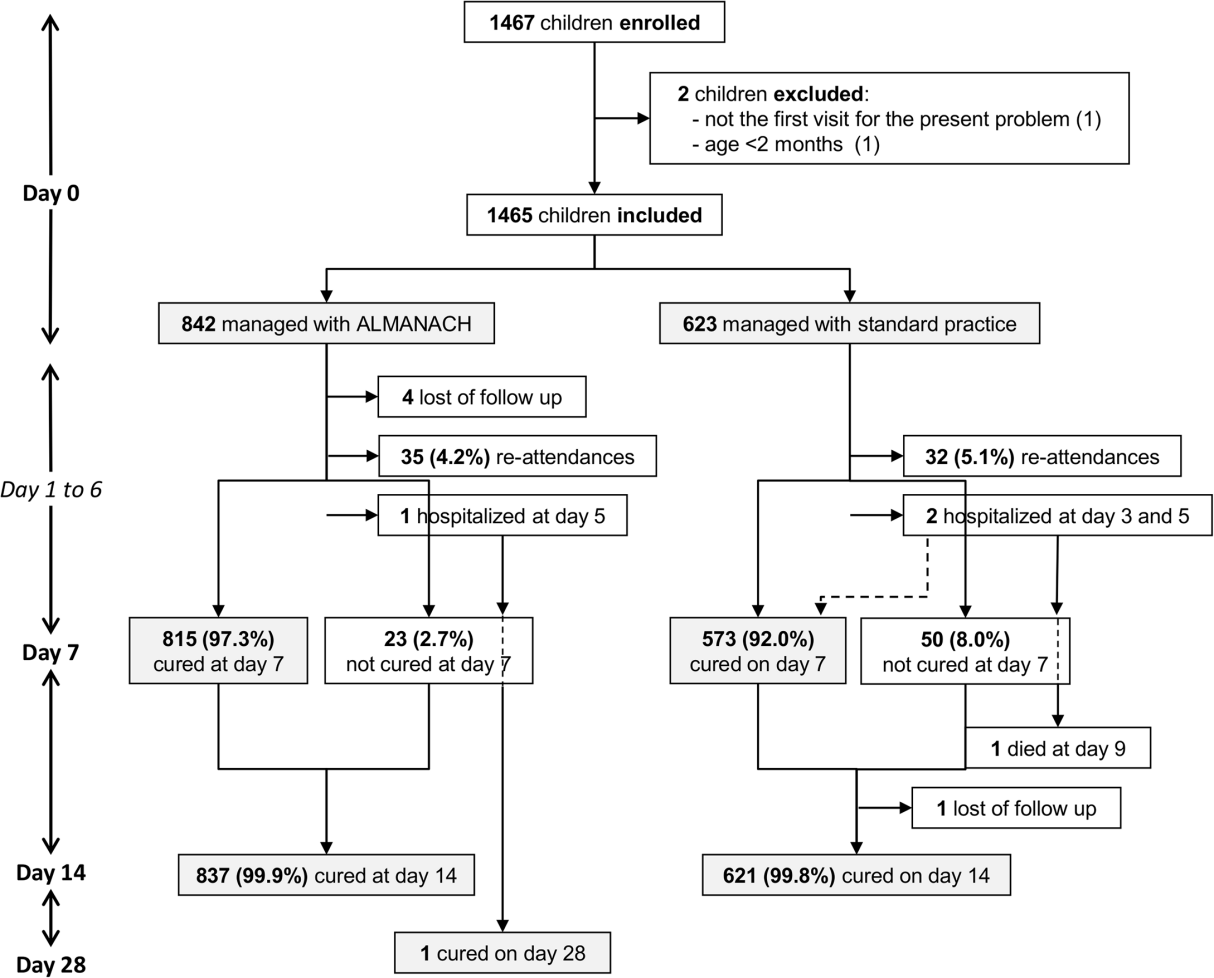
Study profile.

0∙5% (4/842) of children in the ALMANACH and 0∙2% (1/623) in the standard practice arm were lost of follow-up. 97∙3% children managed with ALMANACH were cured on day 7 versus 92∙0% by standard practice (p<0∙001) (Table 3). In the ALMANACH arm, of the 23 children not cured at day 7, 11 received an antibiotic on day 7; 22 were cured on day 14 and one on day 28 (Fig 2). 10 of these 23 (44%) children had been diagnosed at inclusion with skin problems, either alone or in combination with another diagnosis, 7 (30%) with pneumonia, 7 (30%) with URTI, 3 (13%) with likely viral infection outside URTI, and one patient with acute ear infection (Table 4). In the control arm, of the 50 children not cured on day 7, 48 children were cured on day 14, one child died and one was lost of follow-up (Fig 2).

**Table 3:** Clinical outcome and antimicrobials prescribed in children managed by ALMANACH and standard practice.

Outcome measure	ALMANACH			Standard practice			p-value
	n/N	%	95%CI	n/N	%	95%CI	
**Clinical outcome**							
Cured on day 7	815/838	97∙3	96∙1–98∙4	573/623	92∙0	89∙8–94∙1	<0∙001
Cured on Day 14	837/838	99∙9	96∙6–100.1	621/622	99∙8	99∙5–100∙2	0∙8
**Antibiotics prescribed**							
On day 0	130/842	15∙4	12∙9–17∙9	525/623	84∙3	81∙4–87∙1	<0∙001
Between day 0 and 7	19/838	2∙3*	1∙3–3∙3	20/623	3∙2	1∙8–4∙6	0∙3
On day 7	11/838	1∙3	0∙7–2∙3	0/623	0	0	0∙003
Total (at any day)	160/838	19∙0	16∙3–21∙6	545/623	87∙5	84∙9–90∙1	<0∙001
**Antimalarials prescribed**							
On day 0	33/842	3∙9	2∙6–5∙2	41/623	6∙6	4∙6–8∙5	0∙02
Between day 0 and 7	1/838	0∙1	-0∙1–0∙4	7/623	1∙1	0∙3–2∙0	0∙01
On day 7	0/838	0	0	2/623	0∙3	-0∙1–0∙8	0∙2
Total (at any day)	34/838	4∙1	2∙7–5∙4	50/623	8∙0	5∙9–10∙2	0∙001

* 10 patients received antibiotics from study clinicians during working hours and 9 from routine clinicians out of working hours.

**Table 4:** Characteristics of the 23 patients who were not cured at day 7 in the ALMANACH arm. URTI = Upper respiratory tract infection, RR = respiratory rate, NA = not available.

N°	Age (months)	Diagnosis at day 0	RR at day 0	Antibiotic prescribed on day 0	Diagnosis at day 7	Antibiotic prescribed on day 7	Hospitalized	Cured at day 14
1	2	Severe pneumonia	NA	Yes	Pneumonia	Yes	No	Yes
2	6	Pneumonia	52	Yes	Pneumonia	Yes	No	Yes
3	6	Pneumonia	55	Yes	Pneumonia	Yes	No	Yes
4	7	Pneumonia	NA	Yes	URTI and dysentery	Yes	No	Yes
5	12	Pneumonia	53	Yes	URTI and diarrhoea	No	No	Yes
6	13	Pneumonia	66	Yes	URTI	No	No	Yes
7	7	Pneumonia and impetigo	NA	Yes	Infected heat rashes	No	No	Yes
8	23	URTI	NA	No	Diarrhoea	No	No	Yes
9	18	URTI	NA	No	URTI	No	No	Yes
10	9	URTI	NA	No	Diarrhoea	No	No	Yes
11	15	URTI and impetigo	32	No	URTI and soft tissue infection	Yes	No	Yes
12	10	URTI and impetigo	44	No	Impetigo	No	No	Yes
13	7	URTI, diarrhoea and impetigo	36	No	Pneumonia and diarrhoea	Yes	No	Yes
14	10	URTI and scabies	NA	No	Pneumonia	Yes	No	Yes
15	12	Impetigo		No	Soft tissue infection	Yes	No	Yes
16	16	Impetigo		No	Soft tissue infection	Yes	No	Yes
17	10	Infected skin rashes		No	Skin abscess	Yes	No	Yes
18	3	Scabies		No	URTI	No	No	Yes
19	16	Fungal infection		No	Fungal infection	No	No	Yes
20	12	Likely viral infection	NA	No	URTI	No	No	Yes
21	20	Likely viral infection	NA	No	URTI	No	No	Yes
22*	11	Likely viral infection	NA	No	Cellulitis	Yes	Yes	No
23	38	Acute ear infection		No	Acute ear discharge	Yes	No	Yes

* This patient is the same as patient n°8 in Table 5. He was secondarily admitted for cellulitis on day 5, received antibiotics on admission, was discharged after 10 days and was cured on day 28.

Among diagnoses found in at least 10 children at inclusion, pneumonia (7/101 = 7∙0%), skin conditions alone (5/84 = 6∙0%) and multiple conditions not requiring antibiotics (4/81 = 4∙9%) were the conditions leading to the highest proportions of clinical failure at day 7 in the ALMANACH arm, and ARI (13/119 = 11∙0%), skin conditions (3/28 = 11∙0%) and diarrhea (3/33 = 9∙0%) in the standard practice arm.

#### Complications

Of the 838 children managed with ALMANACH, one child with likely viral infection on day 0 was brought by the caretaker on day 5 to a referral hospital where he was diagnosed with cellulitis. He was hospitalized for 10 days, received antibiotics and had recovered when visited on day 28. Of the 623 children managed by standard practice, two children were hospitalized secondarily. One had diarrhoea on day 0 and received cotrimoxazole, oral rehydration salt and zinc tablets. At day 3 he was brought to the same HF and diagnosed with severe dehydration. He was admitted for one day, received ringer lactate intravenously and was discharged the next day. At day 7 he had recovered. The other child was diagnosed with pneumonia on day 0, received benzyl penicillin and amoxicillin, and was sent home. He was brought 5 days later to another HF where he was admitted for the same diagnosis and died 4 days later (see Fig 2).

#### Spontaneous attendance before day 7

4∙2% of children (35/838) in the ALMANACH and 5∙1% (32/623) in the standard practice arm re-attended spontaneously between day 0 and 7 (p = 0∙4). In the intervention arm, 19 (2∙3%) patients were secondarily prescribed an antibiotic, 10 because of pneumonia, 5 diarrhea, 2 UTI, 1 cellulitis and one tonsillitis (Table 5). All were cured at day 7, except the one who was hospitalized with cellulitis. Among 8 children who developed pneumonia secondarily, 6 were <12 months and had a RR at inclusion between 36 and 48/min, and 2 were ≥12 months and one had a RR of 42 (not measured for the other child due to absence of cough). On the other hand, 30 children ≥12 months with a RR between 40 and 50/min at inclusion did not develop pneumonia, and were cured at D7 without antibiotic.

**Table 5:** Characteristics of the 19 patients who received antibiotics during re-attendance in the ALMANACH arm.

N°	Age (months)	Diagnosis at day 0	Respiratory rate at day 0	Antibiotic prescribed on day 0	Clinician who prescribed antibiotics*	Diagnosis at re-attendance visit	Cured at day 7
1	10	Pneumonia	53	Yes	Study	Pneumonia	Yes
2	8	Pneumonia	NA	Yes	Routine	Pneumonia	Yes
3	10	Pneumonia	52	Yes	Study	UTI	Yes
4	8	Pneumonia and measles	52	Yes	Routine	Diarrhoea	Yes
5	18	Likely viral infection	NA	No	Study	Pneumonia	Yes
6	21	Likely viral infection	NA	No	Study	Tonsillitis	Yes
7	56	Likely viral infection	NA	No	Study	UTI	Yes
8^&^	11	Likely viral infection	NA	No	Study	Cellulitis	No
9	10	Likely viral infection	NA	No	Routine	Diarrhoea	Yes
10	37	Likely viral infection	NA	No	Routine	Diarrhoea	Yes
11	6	URTI	46	No	Study	Pneumonia	Yes
12	4	URTI	NA	No	Study	Pneumonia	Yes
13	5	URTI	NA	No	Routine	Pneumonia	Yes
14	11	URTI	40	No	Routine	Pneumonia	Yes
15	13	URTI	42	No	Routine	Pneumonia	Yes
16	8	URTI and diarrhoea	48	No	Study	Pneumonia	Yes
17	5	UTI		Yes	Study	Pneumonia	Yes
18	9	Diarrhoea		No	Routine	Diarrhoea	Yes
19	36	Impetigo		No	Routine	Diarrhoea	Yes

*Out of working hours, the patient was evaluated and managed by a routine clinician of the HF rather than the study clinician.

^&^This patient is the same as patient n°22 in Table 4.

### Treatment prescribed

15∙4% (130/842) of children managed with ALMANACH received antibiotics on day 0 compared to 84∙3% (525/623) by standard practice (p<0∙001). In the ALMANACH arm, only 19 (2∙3%) and 11 (1∙3%) children out of 842 received antibiotics secondarily, during spontaneous attendance and visit at day 7 respectively. The cumulative proportion of children prescribed antibiotics over the whole follow-up period (on day 0, between day 0 and day 7 and on day 7) was 19∙0% in the intervention versus 87∙5% in the control arm (p<0∙001) (Table 3). In the ALMANACH arm, the diagnoses at inclusion present in at least 10 patients for which antibiotics were most frequently prescribed secondarily were pneumonia (8/101 = 7∙9%), likely viral infection (6/96 = 6∙3%) and skin problem (4/84 = 4∙8%).

## Discussion

When strictly applied, the new ALMANACH algorithm resulted in better clinical outcome than standard practice, and in 80% reduction of antibiotics prescribed to children with acute illness. These improvements are probably due to a better identification of children with likely viral infection, and hence not needing antibiotics, while still identifying those with bacterial infections, or at least those who were likely to benefit from antibiotics.

The rate of clinical failure with ALMANACH was expected to be equivalent to that of the control arm, because the standard practice in Tanzania is to prescribe antibiotics to most of the febrile patients, especially when mRDT are available [22]. One could have even expected more failures with ALMANACH since the algorithm withholds antibiotics (compared to IMCI) in frequent clinical situations such as cough and RR between 40 and 50/min in children ≥12 months, or for children without a classification at the end of the algorithm (likely viral infection). On the contrary, we observed a better cure rate with ALMANACH, probably because clinicians were able to better identify and treat children with possible bacterial infection. Moreover, the better outcome at day 7 was neither at the price of a higher rate of spontaneous re-attendance, nor of secondary prescription of antibiotics. These rates were indeed almost identical in both arms. These findings suggest that significant bacterial infections were not missed when using ALMANACH, which is the big fear of clinicians and their main reason to give antibiotics. They often wrongly believe that antibiotics prevent secondary bacterial infections. Their behavior is also due to clinical guidelines that are often ambiguous, including the latest 2014 version of IMCI that recommends to ‘Give appropriate antibiotic treatment for an identified bacterial cause of fever’ to febrile children that are negative for malaria [29]. Such a recommendation has a high risk to increase over-prescription of antibiotics. Policy makers sometimes argue that children will not be brought back if their condition worsens, because of long distance from home to health facilities, lack of transport and of cash money etc. The good clinical outcome observed in the intervention arm suggests that caretakers did come back when their child was worse, maybe because of clear messages given by clinicians. The present study thus demonstrates that giving antibiotics to all children at first place to prevent re-attendances or complications is not worth; it does not improve clinical outcome, provided the few children who need antibiotics are accurately identified.

Giving unnecessary antibiotics does have deleterious consequences, namely the rapid spread of bacterial resistance, unnecessary adverse drug reactions, and unnecessary cost. In Tanzania, high levels of antibiotic resistance have already been reported [30, 31]. Also, children infected with resistant microorganisms are more likely to die [32]. Unfortunately the different approaches to reduce antibiotic prescription have been largely ineffective. In a systematic review, educational/training interventions successfully improved targeted antibiotic prescribing outcomes by only 20%, and these changes were not sustainable over time [33]. Holistic strategies are needed to contain antibiotic resistance, including the use of electronic decision support to improve clinician’s compliance to guidelines. Such a strategy using electronic algorithms for the management of childhood illness in a rural dispensary in Tanzania showed promises [34]. The next step is thus to further evaluate this electronic ALMANACH in programmatic conditions.

### Clinical failure and/or secondary antibiotic prescription according to diagnosis type in ALMANACH arm

Among children managed using ALMANACH, the diagnosis that led to the highest rate of clinical failure was pneumonia (7%), which also led to the highest rate of secondary antibiotic prescription (8%). In contrast, URTI was rarely associated with clinical failures (1%) or secondary antibiotic prescription (2%). Because the vast majority of ARI are located in the upper tract and of viral origin, these children do not require antibiotics and cure by themselves. In young children, even most of lower respiratory tract infections, including pneumonias, are due to viruses and will thus not improve with the provision of antibiotics. This also explains why a significant number of these patients were not cured at day 7.

The second diagnosis that led to the highest rate of clinical failure (6%) and secondary antibiotic prescription (5%) was skin conditions. Skin problems are not included in the main algorithm of IMCI algorithm. Half of the skin problems were mild infections such as impetigo that had worsened enough to require antibiotics at day 7. The other half corresponded to skin problems that took longer than 7 days to cure such as fungal infection or stable impetigo, but that did not require secondary antibiotics.

A ‘likely viral infection’ was the third diagnosis that led to a relatively high rate (6%) of secondary antibiotic prescription, but to a rather low rate (3%) of clinical failure at day 7. Antibiotics were given during follow-up because of the emergence of various conditions such as pneumonia, tonsillitis, UTI, cellulitis and diarrhea. This diversity shows that it is not possible to predict at day 0 if, and what these children may develop in the following days. The only safe and rational solution is thus to evaluate them again when they do not improve. The aim of an efficient clinical algorithm is indeed not to have zero follow-up visits, but rather to have no child dying because of a delay once antibiotic are required. The message to bring the child back in case of persisting or worsening condition, or emergence of a new health problem seems to have been followed appropriately, as only one child has been secondarily admitted. When used wisely, it prevents a lot of unnecessary prescription of antibiotics during first clinical encounter.

### Limitations of the study

One can argue that the appropriate control arm would have been a perfectly complied to IMCI algorithm. However, no study on the clinical outcome of children strictly managed according to IMCI has been performed in the past, so such results could not be used as gold standard. Also a perfectly implemented IMCI does not exist, which shows its limitation in terms of feasibility. We opted thus for the use of IMCI in real life conditions (routine practice) for the control arm to assess more precisely the public health benefit of the ALMANACH.

The new algorithm was implemented in controlled conditions, which is a necessary step before implementation in routine conditions. Its real impact, which should directly depend on the level of uptake and compliance by clinicians, needs to be precisely evaluated. We already performed this step in a study investigating health worker’s performance when using ALMANACH in pragmatic conditions (reported in Rambaud-Althaus et al, submitted).

No formal assessment of health worker satisfaction when using electronic devices was made in the present study. Previous findings from a pilot study conducted in Tanzania assessing the use of electronic IMCI showed that clinicians were enthusiastic to use it. However, this was not enough justification to believe that the clinicians would indeed follow the “standard practice ALMANACH” better than standard/routine practice. We also performed subsequently a qualitative study to assess health workers’ perception on barriers and facilitators for uptake of the ALMANACH algorithm in pragmatic conditions over time (reported in Shao et al, submitted).

## Conclusion

The new ALMANACH algorithm for the management of childhood illness, primarily aimed at the rational use of antimicrobials, improved clinical outcome and led to a drastic reduction of unnecessary antibiotic prescription when compared to standard practice. This achievement was related to more precise diagnoses and better identification of children with infections that required and did not require antibiotics. These results, obtained in both urban and rural places, are probably generalizable for most locations in Sub-Saharan Africa, and even wider, since the distribution of diagnoses in small children does not vary so much across regions and over-prescription of antibiotics is a widespread problem in low resource settings [35]. The building on mobile technology allowed easy access for clinicians and rapid update of the decision chart when new recommendations are put in place. Further studies are underway to assess the appropriateness and feasibility of using this electronic algorithm in routine practice.

## Supporting Information

S1 File A companion ALMANACH development paper. (PDF)

S2 File The study protocol. (PDF)

S3 File Consort statement for non-inferiority trial. (DOCX)

S4 File Simplified ALMANACH. (PDF)

S5 File ALMANACH application user manual. (PDF)
