# Supplementary material for: New Algorithm for Managing Childhood Illness Using Mobile Technology (ALMANACH): A Controlled Non-Inferiority Study on Clinical Outcome and Antibiotic Use in Tanzania
Source: PLoS One. 2015 Jul 10;10(7):e0132316. doi: 10.1371/journal.pone.0132316 (PMC4498627; doi:10.1371/journal.pone.0132316)
Supplement: S1 File — (PDF) [file pone.0132316.s001.pdf]

PLOS ONE

Managing the sick child in the era of declining malaria transmission: development of ALMANACH, an evidence-based electronic algorithm for appropriate use of antimicrobials

--Manuscript Draft--

**Manuscript Number:**	
**Article Type:**	Research Article
**Full Title:**	Managing the sick child in the era of declining malaria transmission: development of ALMANACH, an evidence-based electronic algorithm for appropriate use of antimicrobials
**Short Title:**	ALMANACH, an electronic algorithm for childhood illness
**Corresponding Author:**	Clotilde Rambaud Althaus, MD<br>Swiss Tropical and Public Health Institute<br>Basel, Schweiz SWITZERLAND
**Keywords:**	electronic clinical decision support system; child health; Mobile Health; integrated management of childhood illness; primary health care; Tanzania; ALMANACH; non-malaria fever; pediatrics; under 5
**Abstract:**	Objective:<br>To review the available evidence on Childhood illness and develop an electronic algorithm for the Integrated Management of Childhood Illness to reach optimal clinical outcome and rational use of medicines<br>Methods:<br>A structured literature review in Medline, Embase and the Cochrane Database of Systematic Review (CDRS) looked for available evidence on i) diseases prevalence in pediatric outpatients, ii) accuracy of clinical predictors, and iii) performance of point-of-care tests for targeted diseases. A new algorithm for the management of childhood illness (ALMANACH) was designed based on evidence retrieved and results of a study on etiologies of fever in Tanzanian children outpatients.<br>Findings:<br>The major changes in ALMANACH compared to IMCI (2008 version) are the following: i) assessment of 10 danger signs, ii) classification of non-severe children into febrile and non-febrile illness, the latter receiving no antibiotics, iii) classification of pneumonia based on a respiratory rate threshold of 50 assessed twice for febrile children 12-59 months; iv) malaria rapid diagnostic test performed for all febrile children. In the absence of identified source of fever at the end of the assessment, v) urine dipstick performed for febrile children <2years to consider urinary tract infection, vi) classification of 'possible typhoid' for febrile children >2 years with abdominal tenderness; and lastly vii) classification of 'likely viral infection' in case of negative results.<br>Conclusion:<br>This smartphone-run algorithm based on new evidence and two point-of-care tests should improve the quality of care of <5 year children and lead to more rational use of antimicrobials.
**Order of Authors:**	Clotilde Rambaud Althaus, MD
	Amani Flexson Shao
	Judith Kahama-Maro
	Blaise Genton
	Valerie D'Acremont
**Suggested Reviewers:**	Shamim A Qazi, MD<br>World Health Organization<br>qazis@who.int<br>Dr Qazi is an international expert on childhood illness management and has leaded

Powered by Editorial Manager® and ProduXion Manager® from Aries Systems Corporation

	research on pneumonia diagnosis and management in order to refine Integrated Management of Childhood Illness
	Eric A Simoes, MD<br>Professor, Children's Hospital, Colorado<br>eric.simoes@ucdenver.edu<br>Prof Simoes has been leading early research for the design and validation of the 'Integrated Management of Childhood Illness' clinical algorithms. He is still leading research to bring new evidence to improve the these algorithm.
**Opposed Reviewers:**	
**Additional Information:**	
**Question**	**Response**
**Financial Disclosure**<br><br>Please describe all sources of funding that have supported your work. A complete funding statement should do the following:<br><br>Include **grant numbers and the URLs** of any funder's website. Use the full name, not acronyms, of funding institutions, and use initials to identify authors who received the funding.<br>**Describe the role** of any sponsors or funders in the study design, data collection and analysis, decision to publish, or preparation of the manuscript. If they had no role in any of the above, include this sentence at the end of your statement: "*The funders had no role in study design, data collection and analysis, decision to publish, or preparation of the manuscript.*"<br><br>If the study was **unfunded**, provide a statement that clearly indicates this, for example: "*The author(s) received no specific funding for this work.*"<br><br>* typeset	The study was part of a larger project which aimed at improving the quality of health care and rational use of drugs for children in Tanzania (PeDiAtrick project), funded by the Swiss National Science Foundation (www.snf.ch; Grant Number IZ70Z0 – 124023). The funders played no role in study design, collection, analysis, interpretation of data, writing the report, or in the decision to submit the paper for publication.<br>Pan Africa Clinical Trial registration number PACTR201011000262218
**Competing Interests**<br><br>You are responsible for recognizing and disclosing on behalf of all authors any competing interest that could be perceived to bias their work, acknowledging all financial support and any other relevant financial or non-financial competing interests.<br><br>Do any authors of this manuscript have competing interests (as described in the PLOS Policy on Declaration and	The authors have declared that no competing interests exist.

Evaluation of Competing Interests)?<br><br>**If yes**, please provide details about any and all competing interests in the box below. Your response should begin with this statement: *I have read the journal's policy and the authors of this manuscript have the following competing interests:*<br><br>**If no** authors have any competing interests to declare, please enter this statement in the box: "*The authors have declared that no competing interests exist.*"<br><br>* typeset	
**Ethics Statement**<br><br>You must provide an ethics statement if your study involved human participants, specimens or tissue samples, or vertebrate animals, embryos or tissues. All information entered here should **also be included in the Methods section** of your manuscript. Please write "N/A" if your study does not require an ethics statement.<br><br>**Human Subject Research (involved human participants and/or tissue)**<br><br>All research involving human participants must have been approved by the authors' Institutional Review Board (IRB) or an equivalent committee, and all clinical investigation must have been conducted according to the principles expressed in the Declaration of Helsinki. Informed consent, written or oral, should also have been obtained from the participants. If no consent was given, the reason must be explained (e.g. the data were analyzed anonymously) and reported. The form of consent (written/oral), or reason for lack of consent, should be indicated in the Methods section of your manuscript.<br><br>Please enter the name of the IRB or Ethics Committee that approved this study in the space below. Include the approval number and/or a statement indicating approval of this research.	N/A

Powered by Editorial Manager® and ProduXion Manager® from Aries Systems Corporation

**Animal Research (involved vertebrate animals, embryos or tissues)**<br><br>All animal work must have been conducted according to relevant national and international guidelines. If your study involved non-human primates, you must provide details regarding animal welfare and steps taken to ameliorate suffering; this is in accordance with the recommendations of the Weatherall report, "The use of non-human primates in research." The relevant guidelines followed and the committee that approved the study should be identified in the ethics statement.<br><br>If anesthesia, euthanasia or any kind of animal sacrifice is part of the study, please include briefly in your statement which substances and/or methods were applied.<br><br>Please enter the name of your Institutional Animal Care and Use Committee (IACUC) or other relevant ethics board, and indicate whether they approved this research or granted a formal waiver of ethical approval. Also include an approval number if one was obtained.<br><br>**Field Permit**<br><br>Please indicate the name of the institution or the relevant body that granted permission.	
**Data Availability**<br><br>PLOS journals require authors to make all data underlying the findings described in their manuscript fully available, without restriction and from the time of publication, with only rare exceptions to address legal and ethical concerns (see the PLOS Data Policy and FAQ for further details). When submitting a manuscript, authors must provide a Data Availability Statement that describes where the data underlying their manuscript can be found.<br><br>Your answers to the following constitute your statement about data availability and will be included with the article in the event of publication. **Please note that simply stating 'data available on request from the author' is not acceptable.** ***If,***	Yes - all data are fully available without restriction

however, your data are only available upon request from the author(s), you must answer "No" to the first question below, and explain your exceptional situation in the text box provided.<br><br>Do the authors confirm that all data underlying the findings described in their manuscript are fully available without restriction?	
Please describe where your data may be found, writing in full sentences. **Your answers should be entered into the box below and will be published in the form you provide them, if your manuscript is accepted.** If you are copying our sample text below, please ensure you replace any instances of XXX with the appropriate details.<br><br>If your data are all contained within the paper and/or Supporting Information files, please state this in your answer below. For example, "All relevant data are within the paper and its Supporting Information files."<br>If your data are held or will be held in a public repository, include URLs, accession numbers or DOIs. For example, "All XXX files are available from the XXX database (accession number(s) XXX, XXX)." If this information will only be available after acceptance, please indicate this by ticking the box below.<br>If neither of these applies but you are able to provide details of access elsewhere, with or without limitations, please do so in the box below. For example:<br><br>"Data are available from the XXX Institutional Data Access / Ethics Committee for researchers who meet the criteria for access to confidential data."<br><br>"Data are from the XXX study whose authors may be contacted at XXX."<br><br>* typeset	Data will be made available after acceptance
Additional data availability information:	Tick here if the URLs/accession numbers/DOIs will be available only after acceptance of the manuscript for publication so that we can ensure their inclusion before publication.

Dr. Clotilde Rambaud Althaus
Health Interventions Unit
Epidemiology and Public Health department
Swiss Tropical and Public Health Institute
Socinstrasse 57, CH- Basel
+4176 445 15 83
Clotilde.Rambaud@unibas.ch

Plos One
Editor

Basel, October 2nd 2014,

Dear Editor,

Thank you for considering our manuscript entitled: **Managing the sick child in the era of declining malaria transmission: development of ALMANACH, an evidence-based electronic algorithm for appropriate use of antimicrobials** for publication as an original research article in *Plos One*. ALMANACH is a new electronic clinical algorithm derived from the Integrated Management of Childhood Illness algorithm that includes in particular guidance on the management of non-malaria febrile illnesses. Its aim is to improve health workers' performance at diagnosing bacterial versus viral diseases, in order to ensure optimal clinical outcome and rational use of antibiotics. The algorithm has been developed as an android application for smartphones and tablets to improve adherence of clinicians to the recommendations. This article presents the evidence behind, the method, the content and the technology used for ALMANACH algorithm development.

Our findings and new algorithm address important concerns of stakeholders worldwide, from health workers to policy makers, namely the absence of guidance regarding non-malaria fevers, the low specificity of the criteria used in IMCI to decide on antibiotic prescription and the low compliance of health workers to existing paper guidelines which leads to overprescription of antimicrobials. It takes into account the new evidence gathered from recent studies on etiology of fevers and systematic reviews on clinical predictors, as well as the availability of point-of-care diagnostic tools. Appropriate clinical management of childhood illness is a topic of great importance in low income countries, where trained health workers are scarce, and antibiotic resistance is rapidly spreading.

Our paper is submitted as a companion paper to that of Dr A. Shao et al entitled "**New algorithm for managing childhood illness using mobile technology (ALMANACH): a controlled non-inferiority study on clinical outcome and antibiotic use in Tanzania**", which showed that when used in controlled conditions, ALMANACH has led to improved clinical outcome and considerable reduction of antimicrobial prescription.

During a pilot implementation, ALMANACH, used in programmatic conditions, has also shown to lead to higher health workers performance and reduced antibiotic prescription. The paper describing this pilot implementation, in preparation, could also be submitted to Plos One soon.

This paper has not been considered for publication in any other journal.

We hope that you will acknowledge the importance and novelty of this tool that should improve the quality health delivered to children.

Yours sincerely,

Clotilde Rambaud Althaus
MD
Research Physician, PhD candidate,
Swiss TPH and Basel University

Manuscript
Click here to download Manuscript: Rambaud et al.ALMANACHdevelopment_manuscript_without ref_ PlosOne.docx

Managing the sick child in the era of declining malaria transmission: development of ALMANACH, an evidence-based electronic algorithm for appropriate use of antimicrobials

Clotilde Rambaud Althaus[1*], Amani Flexson Shao[2], Judith Kahama-Maro[3†], Blaise Genton[1,4,5], Valérie d'Acremont[1,4,5].

[1]Epidemiology and Public Health department, Swiss Tropical and Public Health Institute, Basel, Switzerland,

[2] Tukuyu Medical Research Center, National Institute for Medical Research, Tukuyu, Tanzania,

[3]City Medical Office of Health, Dar es Salaam City Council, Dar es Salaam, Tanzania

[4]Infectious Disease Service, Lausanne University Hospital, Lausanne, Switzerland

[5]Department of Ambulatory Care and Community Medicine, University of Lausanne, Switzerland

*Corresponding Author:

e-mail: Clotilde.Rambaud@unibas.ch (CRA)

[†]Deceased

Word count:

Abstract: 248 words. Manuscript: 4083 words.

Number of Tables and Figures:

Tables: 2, Figures: 4

// Abstract

Objective:

To review the available evidence on Childhood illness and develop an electronic algorithm for the Integrated Management of Childhood Illness to reach optimal clinical outcome and rational use of medicines

Methods:

A structured literature review in Medline, Embase and the Cochrane Database of Systematic Review (CDRS) looked for available evidence on i) diseases prevalence in pediatric outpatients, ii) accuracy of clinical predictors, and iii) performance of point-of-care tests for targeted diseases. A new algorithm for the management of childhood illness (ALMANACH) was designed based on evidence retrieved and results of a study on etiologies of fever in Tanzanian children outpatients.

Findings:

The major changes in ALMANACH compared to IMCI (2008 version) are the following: i) assessment of 10 danger signs, ii) classification of non-severe children into febrile and non-febrile illness, the latter receiving no antibiotics, iii) classification of pneumonia based on a respiratory rate threshold of 50 assessed twice for febrile children 12-59 months; iv) malaria rapid diagnostic test performed for all febrile children. In the absence of identified source of fever at the end of the assessment, v) urine dipstick performed for febrile children <2years to consider urinary tract infection, vi) classification of 'possible typhoid' for febrile children >2 years with abdominal tenderness; and lastly vii) classification of 'likely viral infection' in case of negative results.

Conclusion:

This smartphone-run algorithm based on new evidence and two point-of-care tests should improve the quality of care of <5 year children and lead to more rational use of antimicrobials.

Introduction

The rapid spread of resistant pathogens worldwide calls for urgent action to improve the rational use of antimicrobials. In low and middle income countries, where infectious diseases childhood mortality is high[1], substandard drugs, auto-medication and health workers (HWs)' over-prescription of antimicrobials are driving the rapid spread of antimicrobial resistance. [2,3] Recent experience in malaria case management has shown that using appropriate diagnostic tools (malaria rapid diagnostic tests – mRDT) has the potential to improve rational use of antimalarial[4–6] without negative impact on health outcome. [4,7–10] Unfortunately it has often been accompanied with an increased antibiotics prescription[4,5,11], reflecting the challenge faced by HWs in front of a negative malaria test result, where diagnostic tools and skills to rule out bacterial diseases are scarce.

To support HWs' decision making in the management of a sick child in low resource settings, WHO and UNICEF have developed the Integrated Management of Childhood Illness (IMCI) clinical algorithm in the mid 90's[12]. The IMCI guidelines rely on the classification of patients based on clinical signs that can be recognized by trained HWs even if their educational background is limited[12]; no laboratory test was included in the IMCI version of 2008: presumptive malaria treatment was recommended for all febrile children (in high malaria risks area). Other causes of fever were not considered (except if the child presented also a complaint leading to another branch of the algorithm). With the advent of new evidence on etiologies and management of childhood illness and reliable point-of-care tests (POCTs), there is a need to rethink the IMCI guidelines and to propose a new algorithm for the management of acute medical illness for children aged 2 to 59 months living in low resource settings. This new algorithm should integrate reliable POCTs and, when the latter are not available, clinical predictors for

acute illnesses, so that evidence-based guidance is provided to clinicians to decide on withholding antimalarials and antibiotics when not beneficial to the child.

An algorithm developed for HWs in remote primary health care facilities (PHCF) should rely on simple clinical signs and easy-to-perform POCTs. Its structure should remain simple, although addressing a larger set of diseases may require a more complex one. The use of hand-held electronic technology to deliver the algorithm may facilitate the use of a complex clinical algorithm by HWs of varying backgrounds. Smartphones and tablets have the potential to facilitate the scale-up of the evidence based recommendations in low resource settings.

Methods

Structured literature reviews

In order to identify the relevant diseases to be addressed in the algorithm, data on the causes of global childhood mortality and morbidity from the Child Health Epidemiology Reference Group (CHERG)[13] publications, and from the Global Burden of Disease website[14] were reviewed to assess the burden of diseases in African children. A structured literature review (SLR) was also conducted to understand the clinical presentation (accurate clinical predictors) and diseases' distribution in children under 5 years of age (U5) attending PHCFs in developing countries, as well as appropriate POCTs for the diagnosis of the targeted diseases.

Medline (PubMed), Embase (Ovid), and the Cochrane Database of Systematic Reviews (CDSR) were explored from inception to December 31st 2010, looking for articles assessing i) the prevalence of diseases and clinical features in U5 attending outpatient facilities in developing countries, and ii) the accuracy of diagnostic procedures for each of the targeted diseases. The detailed search strategy is described in Table 1. Papers involving U5 managed for acute medical conditions in ambulatory settings were selected. Studies involving only infants below 3 months

of age or only adults were excluded. For prevalence of syndromes and diseases at PHCF, studies describing the clinical presentation and/or diagnoses presented by U5 attending outpatients facilities in developing countries were selected. For diagnostic procedures of targeted diseases, studies assessing accuracy of either clinical predictors or POCT were chosen. Systematic reviews addressing the questions of interest were also considered. An additional hand searching of reference lists of selected papers completed these searches. In order to better explore the accuracy of the clinical diagnosis for pneumonia, a systematic review of the literature and meta-analyses of studies assessing the diagnostic accuracy of clinical predictors was conducted, reported elsewhere (Rambaud Althaus et al, submitted).

Findings of the study on causes of fever in outpatient Tanzanian children

In a recently published study on etiologies of fever conducted in outpatient clinics in Tanzania (Tanzanian fever study), clinical assessments and laboratory tests were performed in 1005 febrile children aged 2 months to 10 years (95% were U5) to establish the most probable causes of fever[15]. The distribution of diagnoses, overall and stratified by age, in severe and non-severe children was taken into account to select the targeted disease included in the final algorithm. The clinical predictors for the targeted diseases identified in the Tanzanian fever study were also used to build the new algorithm (De Santis et al, in preparation).

Algorithm construction

With the IMCI algorithm for children 2-59 months of age as departure point, the evidence retrieved from the SLRs and from the Tanzanian fever study was used to propose modifications and new recommendations when relevant, and to design a new decision tree. Diseases were included in the algorithm if they were treatable, and responsible for i) high child mortality and

morbidity, ii) high attendance rate at outpatient facilities, and, iii) high antimicrobial prescription rate. Clinical features that could easily be assessed by HWs of varying background and POCT easy to deploy in low resource ambulatory settings were integrated in the classification procedures, when its use improved the classification accuracy. Once the new algorithm was finalized, both a paper booklet and an electronic software running on android smartphones and tablets were developed.

Results

Flow diagrams of studies selection for the SLRs are available in Figure 1.

All modifications made to the IMCI content based on new findings are presented in Table 2. The major changes concerned: malaria and pneumonia diagnosis; otitis media treatment; the addition of urinary tract infection (UTI) and possible typhoid fever; and a new classification entitled "likely viral infection". The most important modifications are discussed below.

Selection of syndromes or diseases to be addressed by the algorithm

Estimations of burden of diseases by CHERG[1] and IHME[14,16] reported that low respiratory tract infections/pneumonia, malaria, and diarrhea were the leading causes of child mortality in 2010, globally and in Sub Saharan Africa (SSA). These 3 infectious diseases were estimated to be responsible for more than 40% of U5 deaths in SSA. They were also the leading causes of morbidity, responsible for 41% of the total 2010 DALYs in SSA[14]. Other frequent causes of child mortality were HIV/AIDS (3.5 to 4% of U5 deaths in SSA[1,14]), meningitis (3 to 4%[1,14]), measles (1%[1,14]), and tuberculosis (0.8%[14]). In infants aged 1 to 11 months, pertussis (2.8% of deaths in 1-11months infant in SSA) and syphilis (2.3%) were also frequent causes of death[14]. In children aged 1 to 4 years, typhoid fever was estimated to be responsible

for 0.6% of both DALYs and deaths, and bacterial skin diseases for 0.7% of DALYs, and 0.2% of deaths[14].

The SLR identified 22 articles assessing either symptoms or diagnoses distributions, or both, in children attending outpatient facilities in developing countries. In all selected papers assessing symptoms, fever (by history or measured, hereafter referred as fever), cough and diarrhea were the most frequent symptoms reported, respectively by 65 to 93%[17–22], 44 to 82%[17–19,21,22], and 22% to 45%[17–19,21] of children. Diseases of potential bacterial origin reported in the studies retrieved by the SLR were: pneumonia (reported in 5 to 30% of children[15,17–19,22–29]), typhoid fever (3 to 13%[15,22,24]), dysentery (3 to 12% [17,26,27]), otitis media (2 to 12%[7,17–19,26–29]), UTI (1 to 7%[15,22,26,28,30,31]); and meningitis (0 to 3%[17,23,26]). Tonsillitis was reported in 1% of 1005 children in the Tanzanian fever study; all had a negative streptococcal diagnostic test[15]. Another study reported tonsillitis or pharyngitis in 10% of the children, but no streptococcal test was performed[28].

Among the bacterial infections frequently reported, only typhoid fever, UTI and tonsillitis were not yet addressed in IMCI. The fear of these 3 infections is often a reason to prescribe antibiotics in low resource setting. With regards to tonsillitis, early recognition and treatment of streptococcal tonsillitis is of high importance to prevent rheumatic fever and its complications, but prevalence of group A β-hemolytic streptococcus is much lower in U5 than in older children[32], and close to zero in children under 2 years of age[33]. Moreover, acute rheumatic fever and rheumatic heart disease are rare in U5[34,35]. Therefore addressing streptococcal tonsillitis in the management of U5 was considered not to be necessary. UTI and typhoid fever were thus selected to be addressed in the new algorithm, together with the other diseases already addressed in IMCI.

Identification of severe illnesses

In the IMCI algorithm, urgent referral to hospital is recommended in the presence of any of 5 general danger signs (difficulty in drinking, repeated vomiting, had convulsion, lethargy or unconsciousness, convulsing) or in presence of any of the 8 syndrome-related danger signs (fever: stiff neck; cough: stridor or chest indrawing; measles: clouding of cornea or extensive mouth ulcers; malnutrition: severe wasting or oedema of both feet; anemia: severe palmar pallor). Two studies have assessed the accuracy of these IMCI referral criteria to predict hospital referral as decided by routine clinicians in Kenya (sensitivity 46%, specificity 79%)[36], or by a study pediatrician in Bangladesh (sensitivity 86%, specificity 64%)[37]. In the Kenyan study, accuracy of these criteria to predict death in admitted U5 patients was also assessed (sensitivity 89%, specificity 44%)[36]. A systematic review for children in developed countries[38] has also identified reduced consciousness, convulsions, cyanosis, rapid breathing, and slow capillary refill as the strongest predictors of severe illness. Meningeal irritation was also a strong predictor of serious bacterial infection in 3 reported studies [positive likelihood ratio (LR+) ranging from 2.57 to 275] [38]. One of the aims of the present algorithm being to allow early identification and referral of severe conditions and serious bacterial infections, all the IMCI referral criteria were kept although underlying evidence was scarce and criteria's specificity seemed low. In addition, 2 signs that are broadly recognized to be predictors of severity were added to the IMCI general danger signs: cyanosis[38] and jaundice[39]. In order to improve and fasten the identification of severe patients, all general danger signs, were grouped together with stiff neck, severe wasting, and severe pallor at the beginning of the assessment chart, instead of having some of them included in the branches for each syndrome.

Malaria diagnosis

Decline in the proportion of fevers due to malaria [40] together with the availability of easy-to-use, reliable POCTs– i.e. mRDTs – have driven the WHO recommendations to shift in 2010 from

presumptive to test-based malaria case management[41]. The safety of a mRDT-based malaria case management in U5 has been demonstrated[7–10,42–44]. Several African countries have now changed their malaria diagnosis policy and adopted the use of mRDTs in their national programs. Following the new WHO malaria treatment guidelines, the use of mRDTs was integrated in present algorithm. mRDTs were also recently added officially to the WHO/UNICEF generic IMCI algorithm[45].

Pneumonia diagnosis

In a recent meta-analysis of clinical predictors for radiological pneumonia (Rambaud Althaus et al, submitted), the clinical features with the higher pooled LR+ were respiratory rate >50 breaths/min (1.90; 95%CI 1.45-2.48), grunting (1.78; 1.10-2.88), lower chest indrawing (1.76; 0.86-3.58), and nasal flaring (1.75; 1.20-2.56). The features with the best (lowest) pooled LR- were: history of fever (0.53; 0.41-0.69), and respiratory rate <40 breaths/min (0.43; 0.23-0.83). Cough had also a good but heterogeneous LR- (0.30; 0.09-0.96). The IMCI criterion for non-severe pneumonia classification, i.e. age-related fast breathing (>50/min from 2 to 11 months, and >40/min from 12 to 59 months) showed low diagnostic performance in the meta-analysis, with a pooled LR+ of 1.55 (0.44-5.42) and a pooled LR- of 0.63 (0.16-2.55). In the Tanzanian fever study, the best predictors to include radiological pneumonia among all febrile children were difficult breathing (LR+ 7.9, 2.8-22.1), chest indrawing (7.1; 2.9-17.6), nasal flaring (7.0; 2.5-19.4), respiratory rate >50/min (6.1; 3.5-10.4) and abnormal chest auscultation (5.5; 3.7-8.1). No feature was good at excluding the diagnosis. In the present algorithm, in the absence of a reliable point-of-care diagnostic test, we decided to combine the best available clinical predictors (history of fever, cough, difficult breathing and fast breathing), except nasal flaring and grunting because of the difficulty for low level health workers to detect these signs, and abnormal chest auscultation because most clinicians do not have a stethoscope or are not familiar with its use.

Chest indrawing was kept but to decide on referral to hospital rather than to diagnose pneumonia, because of the relatively high proportion of these children that harbor hypoxemia[46] Regarding fast breathing, because using an age-related threshold did not improve the diagnostic test accuracy in the meta-analysis (Rambaud Althaus et al, submitted), a single threshold of >50/min for all age groups was chosen; 50/min rather than 40/min was chosen to ensure a reasonable specificity, knowing that most of pneumonias in young children are due to viruses[47]. The recommendation in the present algorithm is thus to prescribe antibiotics for pneumonia to children with [history of fever or elevated temperature] AND [cough or difficult breathing] AND respiratory rate >50/min,

Otitis Media

In the SLR 7 articles and a systematic review that addressed the question of the accuracy of symptoms and signs for the diagnosis of otitis media were retrieved[48]. In these studies, some otoscopic signs were strongly associated with otitis media diagnosis[48], but in low resource settings otoscopy is not available in ambulatory care. Other symptoms, such as earache, ear rubbing, and fever, although reported as associated with otitis media in 4 old studies (LR+ 3.03 to 7.3[49–51]), were not associated with this diagnosis when reported by parents of children aged 6 to 36 months attending primary care offices in a more recent study(52). Otitis media is often a self-limiting condition in young children. The 2010 Coker[48] and Sanders' Cochrane[52] reviews, looking at available evidence of the benefit of antibiotic treatment for otitis media, report that there is little benefit (compared to placebo) and no evidence that antibiotics reduce complications or recurrence[48,52]. An individual patient data meta-analysis from 6 randomised trial reported that antibiotics were more beneficial in children aged less than 2 years with bilateral otitis media, and in those with both otitis media and otorrhoea. In children with otorrhoea, 60% of controls and 25% of those on antibiotics still had pain, fever or both at 3-7 days, with a rate

difference of -36% (95%CI -53% to -19%) and a number needed to treat of 3, whereas in children without otorrhoea the rate difference and NNT were respectively -14% (-23% to -5%) and 8[53]. Otitis media being often a self-limiting condition in young children, in the absence of accurate non-otoscopic clinical predictors the new algorithm propose to limit antibiotic prescription to children presenting with ear discharge.

Urinary tract infection

Two articles and 12 reviews assessing the accuracy of clinical predictors for the diagnosis of UTI in children were retrieved from the SLR. No additional article since the most recent review published in 2007 was found[54]. The following predictors were identified: temperature >40°C (2 studies, LR+ 3.3; 1.3-8.3[55] and LR+ 3.2; 0.7-15.6[56]), jaundice (LR+ 2.1; 0.3-17.4)[57], and suprapubic tenderness (LR+ 4.4; 1.6-12.4)[58]. The absence of another source of fever on examination increased the probability of UTI (3 studies, summary LR+ 2.8; 1.9-4.3)[54]. Among children ≥2 years, abdominal pain (LR+: 6.3; 2.5-16.0) [57], dysuria (LR+ 2.4; 1.8-3.1)[59] and new-onset of urinary incontinence (LR+ 4.6; 2.8-7.6)[59] also increased the probability of UTI. In the Tanzanian fever study, the following predictors to include UTI were found: pollakiuria (LR+ 3.5; 1.4-8.8), temperature >40°C (3.1; 1.4-7.1), fever for more than 3 days (2.1; 1.2-3.6) and age<2 years (1.4, 1.22-1.57); the best predictors to exclude UTI were: age<3 years (LR- 0.22; 0.07-0.66), no headache (0.27; 0.04-1.89) and no diarrhea (0.33; 0.08-1.32) (De Santis et al, in preparation). Based on these predictors, several national and international guidelines recommend to consider this condition in febrile children below 2 years of age, with no obvious cause of fever[30,60]. No symptom or sign, nor combination of them is predictive enough in this age group to appropriately identify children with UTI. The gold standard (urine culture) is generally not available in low resources ambulatory setting. Urinalysis with urine dipsticks detecting leucocyte esterase and nitrite has been evaluated in many settings: 4 systematic reviews with

meta-analyses estimated sensitivities for leucocyte esterase and/or nitrites to be 81%[61], 88%[62,63], and 93%[30] and specificities 72%[30], 79%[63], 93%[62] and 97%[61]. A dipstick urinalysis negative for both nitrites and leukocyte esterase had a LR- of 0.2 (95% CI, 0.16-0.26)[54]. With either leucocyte esterase or nitrite positive the LR+ was 6.1 (95% CI, 4.3-8.6) , increasing to 28 (95% CI, 17-46) when both leucocyte esterase and nitrite were positive[61]. In 2005, the WHO department of Child and Adolescent Health and Development recommended the use of urinalysis by urine dipstick for the diagnosis of UTI in children wherever dipstick were feasible[60]. With the implementation of the WHO focused antenatal care guidelines, urine dipstick for proteinuria detection have been implemented and are thus available in PHCFs in many African countries. Based on the good diagnostic performance of urine dipstick, and it's feasibility in low resource setting, the new algorithm proposes to perform urine dipstick for the diagnostic of UTI in the patients at higher risk of UTI, i.e. children below 2 years of age having fever with no cause identified during the assessment (but regardless of the malaria test result). For children from 2 to 5 years of age, only those complaining of dysuria are proposed a dipstick urinalysis. Antibiotic treatment for UTI is recommended when either leucocyte esterase or nitrite, or both are positive.

Typhoid fever

Regarding the diagnosis of enteric fever, 6 articles assessing clinical predictors of enteric fever were retrieved [64–69]. Only 2 were conducted in outpatients: one included patients above 15 years of age[67] and the other patients above 4 years of age[66]. None of the studies thus included our target population of U5 outpatients. In the Tanzanian fever study[15], the following predictors to include typhoid where identified: liver pain (LR+ 9.8; 2.7-35.5), abdominal tenderness (7.0; 3.3-15.2), jaundice (6.2; 3.1-12.4) and age >2 years (2.0; 1.6-2.4). To exclude typhoid, only 'rainy season' was predictive (LR- 0.50; 0.27-0.92) (De Santis et al, in

preparation). Jaundice being already included as danger sign and liver pain being difficult to assess in a child, the new algorithm recommends to look for abdominal tenderness in children ≥2 years of age having fever with no cause identified during the child's assessment (regardless of the malaria test result). When present, antibiotic treatment for typhoid fever is indicated.

Likely viral infection

Likely viral infection is a classification proposed in the present algorithm that does not exist in IMCI. Unnecessary antibiotics are often prescribed in febrile children by HWs when they do not manage to reach a diagnosis after their assessment, because they fear to have potentially missed a life-threatening bacterial infection. Because in the present algorithm most of the frequent bacterial infections have been assessed for, the probability that the child is still suffering from one is low if all findings are negative. Therefore, in the absence of danger signs, cough or fast breathing, diarrhea, ear discharge, symptoms of measles, infected skin lesion, abdominal tenderness, a positive dipstick urinalysis and a positive malaria RDT, the child is classified as having a "Likely viral infection". HWs are then proposed to withhold antibiotics and antimalarials, prescribe symptomatic treatment for fever if any, and advise the caretaker on when to come back if symptoms persist or worsen.

Design of the algorithm

Based on the modifications and adjunctions to IMCI that were retained, a new algorithm for the management of childhood illnesses (ALMANACH) was designed. The IMCI assessment in 3 steps was kept - "Assess, Classify, and Treat" - , as well as the color-coded triage system: red for conditions that require urgent referral, orange for conditions requiring specific treatment, and green for condition needing simple counseling and symptomatic home management[12]. The main difference with IMCI is however that ALMANACH is divided into 3 charts. The first chart provides recommendations for assessment of general danger signs and management of severe

patients, the second chart provides recommendations for patients with fever, and the last one for patients without fever (see Figure 2 for an overview of ALMANACH's structure). This 3-charts structure allows fastening the assessment and management of severe children, for whom all recommendations are available in the very first part of the algorithm. This structure also allows a more comprehensive assessment of febrile children, with pneumonia, malaria, UTI and typhoid fever being considered only in febrile children.

ALMANACH was first designed as a paper booklet, for which efforts were made to keep the ALMANACH structure simple and graphically easy to follow by HWs (Figure 3). It was then developed as an android application for smartphones, coding the different steps of the algorithm into a Java-Rosa X form run by OpenDataKit and OpenMRS software[70,71]. The electronic ALMANACH (e-ALMANACH) guides HWs through the child's assessment up to the classification and treatment recommendations (Figure 4). Treatment dosages are computed according to the body weight or age when weight is not available. Moreover e-ALMANACH collects in real time information on child demographic characteristics, disease classification and treatment prescribed. This information is stored by the mobile device, can be sent to a server and feed health information systems.

Discussion

The aim of ALMANACH is to provide evidence-based guidance to health workers on antimicrobial prescription, in order to treat only children aged 2 to 59 months who will potentially benefit from them. Apart from malaria, IMCI was not directly addressing causes of fever, leaving HWs with their fear of life-threatening conditions once malaria was ruled out by mRDT. On the other hand, viral infections that represent the vast majority of the causes of fever in U5 children[15] are never explicitly mentioned or proposed as diagnosis in IMCI, giving a wrong impression to health workers that bacterial infections are frequent and that children should often be prescribed antibiotics. Using the best available and feasible diagnostic procedures for the main causes of acute illness in children attending PHCFs, the present new algorithm should address most of the concerns of HWs regarding bacterial infections and remind them that children often suffer from self-limited viral conditions that do not warrant any specific treatment beside antipyretics. By providing tools to rule out malaria, UTI, and typhoid fever and by proposing a new ‘Likely viral infection’ classification, the use of ALMANACH has thus the potential to improve the health outcome of febrile children and at the same time decrease unnecessary antimalarial and antibiotic prescriptions.

The level of evidence provided by the literature was generally low. The heterogeneity of the findings may be due to uneven quality in study designs, or to the insufficient performance of clinical predictors for the diagnosis of bacterial infections. Within the current project, only the POCTs currently available in low resource settings were considered, constraining the new algorithm to rely mostly on the best available simple clinical predictors. To further improve the quality of the management of pediatric illnesses and the rational use of medicines, accurate and affordable POCTs for bacterial, or even viral infections are highly needed.

While broadening the spectrum of diseases to be addressed, the algorithm became more complex than IMCI. This might be an issue for the targeted audience, i.e. HWs of different background working in low resource ambulatory settings. In order to facilitate understanding and usability of the decision chart, the 3 steps IMCI structure (Assess, Classify and Treat) and the color coded triage, already known by IMCI trained HWs, were kept. Electronic algorithms, by guiding HWs step by step through the algorithm, allow to using a more complex structure with lower risk of misuse. The electronic version of ALMANACH running on smartphones and tablets was designed to address these needs.

The paper and electronic ALMANACH have the potential to improve the management of the sick child. This has been demonstrated in a recently completed feasibility study, which showed the ALMANACH algorithm to improve health outcome of children managed with this tool and to drastically reduce antibiotic prescription (Shao et al, submitted). Further improvement could be brought by integrating other POCT detecting key pathogens once they become available, or even better, by integrating host biomarkers able to predict children in need of antibiotics or at risk of dying.

Acknowledgement

We would like to thank Wilson Were, MD and Mario Gehri, MD for useful discussions and comments on the content and design of the clinical algorithm, and Fabrice Althaus for his participation to the literature reviews and input on methodology.

References

1. Liu L, Johnson HL, Cousens S, Perin J, Scott S, et al. (2012) Global, regional, and national causes of child mortality: an updated systematic analysis for 2010 with time trends since 2000. Lancet 379: 2151–2161. doi:10.1016/S0140-6736(12)60560-1.

2. World Health Organization M (2009) Medicines use in primary care in developing and transitional countries. World Health Organization (WHO). Available: http://www.who.int/medicines/publications/who_emp_2009.3/en/. Accessed 23 July 2013.

3. Mayor S (2010) Better access to drugs in developing countries is accelerating resistance. BMJ 340: c3234.

4. Msellem MI, Mårtensson A, Rotllant G, Bhattarai A, Strömberg J, et al. (2009) Influence of rapid malaria diagnostic tests on treatment and health outcome in fever patients, Zanzibar: a crossover validation study. PLoS Med 6: e1000070. doi:10.1371/journal.pmed.1000070.

5. D'Acremont V, Kahama-Maro J, Swai N, Mtasiwa D, Genton B, et al. (2011) Reduction of anti-malarial consumption after rapid diagnostic tests implementation in Dar es Salaam: a before-after and cluster randomized controlled study. Malar J 10: 107. doi:10.1186/1475-2875-10-107.

6. Thiam S, Thior M, Faye B, Ndiop M, Diouf ML, et al. (2011) Major reduction in anti-malarial drug consumption in Senegal after nation-wide introduction of malaria rapid diagnostic tests. PloS One 6: e18419. doi:10.1371/journal.pone.0018419.

7. D' Acremont V, Malila A, Swai N, Tillya R, Kahama-Maro J, et al. (2010) Withholding Antimalarials in Febrile Children Who Have a Negative Result for a Rapid Diagnostic Test. Clin Infect Dis Off Publ Infect Dis Soc Am. Available: http://www.ncbi.nlm.nih.gov/pubmed/20642354.

8. Mtove G, Hendriksen IC, Amos B, Mrema H, Mandia V, et al. (2011) Treatment guided by rapid diagnostic tests for malaria in Tanzanian children: safety and alternative bacterial diagnoses. Malar J 10: 290. doi:10.1186/1475-2875-10-290.

9. Baiden F, Webster J, Tivura M, Delimini R, Berko Y, et al. (2012) Accuracy of rapid tests for malaria and treatment outcomes for malaria and non-malaria cases among under-five children in rural Ghana. PloS One 7: e34073. doi:10.1371/journal.pone.0034073.

10. Senn N, Rarau P, Manong D, Salib M, Siba P, et al. (2012) Rapid diagnostic test-based management of malaria: an effectiveness study in Papua New Guinean infants with Plasmodium falciparum and Plasmodium vivax malaria. Clin Infect Dis Off Publ Infect Dis Soc Am 54: 644–651. doi:10.1093/cid/cir901.

11. Baiden F, Webster J, Owusu-Agyei S, Chandramohan D (2011) Would rational use of antibiotics be compromised in the era of test-based management of malaria? Trop Med Int Health 16: 142–144. doi:10.1111/j.1365-3156.2010.02692.x.

12. Gove S (1997) Integrated management of childhood illness by outpatient health workers: technical basis and overview. The WHO Working Group on Guidelines for Integrated Management of the Sick Child. Bull World Health Organ 75 Suppl 1: 7–24.

13. Child Health Epidemiology Reference Group (n.d.) CHERG | Publications. Available: http://cherg.org/publications.html. Accessed 25 July 2013.

14. Institute for Health Metrics and Evaluation (n.d.) Global Burden of Disease (GBD) Compare. Available: http://viz.healthmetricsandevaluation.org/gbd-compare/. Accessed 21 November 2013.

15. D'Acremont V, Kilowoko M, Kyungu E, Philipina S, Sangu W, et al. (2014) Beyond Malaria — Causes of Fever in Outpatient Tanzanian Children. N Engl J Med 370: 809–817. doi:10.1056/NEJMoa1214482.

16. Lozano R, Naghavi M, Foreman K, Lim S, Shibuya K, et al. (2012) Global and regional mortality from 235 causes of death for 20 age groups in 1990 and 2010: a systematic analysis for the Global Burden of Disease Study 2010. Lancet 380: 2095–2128. doi:10.1016/S0140-6736(12)61728-0.

17. Kolstad PR, Burnham G, Kalter HD, Kenya-Mugisha N, Black RE (1997) The integrated management of childhood illness in western Uganda. Bull World Health Organ 75 Suppl 1: 77–85.

18. Perkins BA, Zucker JR, Otieno J, Jafari HS, Paxton L, et al. (1997) Evaluation of an algorithm for integrated management of childhood illness in an area of Kenya with high malaria transmission. Bull World Health Organ 75 Suppl 1: 33–42.

19. Weber MW, Mulholland EK, Jaffar S, Troedsson H, Gove S, et al. (1997) Evaluation of an algorithm for the integrated management of childhood illness in an area with seasonal malaria in the Gambia. Bull World Health Organ 75 Suppl 1: 25–32.

20. Barat L, Chipipa J, Kolczak M, Sukwa T (1999) Does the availability of blood slide microscopy for malaria at health centers improve the management of persons with fever in Zambia? Am J Trop Med Hyg 60: 1024–1030.

21. Rowe SY, Olewe MA, Kleinbaum DG, McGowan JE Jr, McFarland DA, et al. (2007) Longitudinal analysis of community health workers' adherence to treatment guidelines, Siaya, Kenya, 1997-2002. Trop Med Int Health TM IH 12: 651–663. doi:10.1111/j.1365-3156.2007.01824.x.

22. Van Hemelrijck MJ, Lindblade KA, Kubaje A, Hamel MJ, Odhiambo F, et al. (2009) Trends observed during a decade of paediatric sick visits to peripheral health facilities in rural western Kenya, 1997-2006. Trop Med Int Health 14: 62–69.

23. Salaria M, Singhi SC (2003) Profile of patients attending pediatric emergency service at Chandigarh. Indian J Pediatr 70: 621–624.

24. Animut A, Mekonnen Y, Shimelis D, Ephraim E (2009) Febrile illnesses of different etiology among outpatients in four health centers in Northwestern Ethiopia. Jpn J Infect Dis 62: 107–110.

25. Chanda P, Hamainza B, Mulenga S, Chalwe V, Msiska C, et al. (2009) Early results of integrated malaria control and implications for the management of fever in under-five children at a peripheral health facility: a case study of Chongwe rural health centre in Zambia. Malar J 8: 49. doi:10.1186/1475-2875-8-49.

26. Factor SH, Schillinger JA, Kalter HD, Saha S, Begum H, et al. (2001) Diagnosis and management of febrile children using the WHO/UNICEF guidelines for IMCI in Dhaka, Bangladesh. Bull World Health Organ 79: 1096–1105.

27. Simoes EA, Desta T, Tessema T, Gerbresellassie T, Dagnew M, et al. (1997) Performance of health workers after training in integrated management of childhood illness in Gondar, Ethiopia. Bull World Health Organ 75 Suppl 1: 43–53.

28. Njama-Meya D, Clark TD, Nzarubara B, Staedke S, Kamya MR, et al. (2007) Treatment of malaria restricted to laboratory-confirmed cases: a prospective cohort study in Ugandan children. Malar J 6: 7. doi:10.1186/1475-2875-6-7.

29. Khallaf N, el-Ansary S, Hassan M (1996) Acute respiratory infections: sentinel survey in Egypt. World Health Forum 17: 297–300.

30. Downs SM (1999) Technical Report: Urinary Tract Infections in Febrile Infants and Young Children. Pediatrics 103: e54–e54.

31. Shaikh N, Morone NE, Bost JE, Farrell MH (2008) Prevalence of Urinary Tract Infection in Childhood: A Meta-Analysis. Pediatr Infect Dis J 27: 302–308. doi:10.1097/INF.0b013e31815e4122.

32. Shaikh N, Leonard E, Martin JM (2010) Prevalence of Streptococcal Pharyngitis and Streptococcal Carriage in Children: A Meta-analysis. Pediatrics 126: e557–e564. doi:10.1542/peds.2009-2648.

33. Woods WA, Carter CT, Schlager TA (1999) Detection of group A streptococci in children under 3 years of age with pharyngitis. Pediatr Emerg Care 15: 338–340.

34. Carapetis JR, Currie BJ, Mathews JD (2000) Cumulative incidence of rheumatic fever in an endemic region: a guide to the susceptibility of the population? Epidemiol Infect 124: 239–244.

35. Tibazarwa KB, Volmink JA, Mayosi BM (2008) Incidence of acute rheumatic fever in the world: a systematic review of population-based studies. Heart 94: 1534–1540. doi:10.1136/hrt.2007.141309.

36. Paxton LA, Redd SC, Steketee RW, Otieno JO, Nahlen B (1996) An evaluation of clinical indicators for severe paediatric illness. Bull World Health Organ 74: 613–618.

37. Kalter HD, Schillinger JA, Hossain M, Burnham G, Saha S, et al. (1997) Identifying sick children requiring referral to hospital in Bangladesh. Bull World Health Organ 75 Suppl 1: 65–75.

38. Van den Bruel A, Haj-Hassan T, Thompson M, Buntinx F, Mant D, et al. (2010) Diagnostic value of clinical features at presentation to identify serious infection in children in developed countries: a systematic review. Lancet 375: 834–845. doi:10.1016/S0140-6736(09)62000-6.

39. World Health Organization, Communicable Diseases Cluster (2000) Severe falciparum malaria. World Health Organization, Communicable Diseases Cluster. Trans R Soc Trop Med Hyg 94 Suppl 1: S1–90.

40. D'Acremont V, Lengeler C, Genton B (2010) Reduction in the proportion of fevers associated with Plasmodium falciparum parasitaemia in Africa: a systematic review. Malar J 9: 240. doi:10.1186/1475-2875-9-240.

41. World Health Organization (2010) Guidelines for the treatment of malaria. 2nd ed. Geneva: World Health Organization. 194 p.

42. Faucher J-F, Makoutode P, Abiou G, Béhéton T, Houzé P, et al. (2010) Can treatment of malaria be restricted to parasitologically confirmed malaria? A school-based study in Benin in children with and without fever. Malar J 9: 104. doi:10.1186/1475-2875-9-104.

43. Mubi M, Janson A, Warsame M, Mårtensson A, Källander K, et al. (2011) Malaria rapid testing by community health workers is effective and safe for targeting malaria treatment: randomised cross-over trial in Tanzania. PloS One 6: e19753. doi:10.1371/journal.pone.0019753.

44. Ukwaja KN, Aina OB, Talabi AA (2010) Outcome of presumptive versus rapid diagnostic tests-based management of childhood malaria - pneumonia overlap in urban Nigeria: a pilot quasi-experimental study. Ethiop J Health Sci 20: 179–183.

45. World Health Organization, UNICEF (2014) Integrated Management of Childhood Illness. Chart booklet. Available: http://apps.who.int/iris/bitstream/10665/104772/16/9789241506823_Chartbook_eng.pdf?ua=1. Accessed 25 March 2014.

46. Subhi R, Adamson M, Campbell H, Weber M, Smith K, et al. (2009) The prevalence of hypoxaemia among ill children in developing countries: a systematic review. Lancet Infect Dis 9: 219–227.

47. Feikin DR, Njenga MK, Bigogo G, Aura B, Aol G, et al. (2013) Viral and bacterial causes of severe acute respiratory illness among children aged less than 5 years in a high malaria prevalence area of western Kenya, 2007-2010. Pediatr Infect Dis J 32: e14–19. doi:10.1097/INF.0b013e31826fd39b.

48. Coker TR, Chan LS, Newberry SJ, Limbos MA, Suttorp MJ, et al. (2010) Diagnosis, microbial epidemiology, and antibiotic treatment of acute otitis media in children. JAMA J Am Med Assoc 304: 2161–2169.

49. Niemela M, Uhari M, Jounio-Ervasti K, Luotonen J, Alho OP, et al. (1994) Lack of specific symptomatology in children with acute otitis media. Pediatr Infect Dis J 13: 765–768.

50. Heikkinen T, Ruuskanen O (1995) Signs and symptoms predicting acute otitis media. Arch Pediatr Adolesc Med 149: 26–29.

51. Uhari M, Niemelä M, Hietala J (1995) Prediction of acute otitis media with symptoms and signs. Acta Paediatr Oslo Nor 1992 84: 90–92.

52. Sanders S, Glasziou PP, Del Mar CB, Rovers MM (2010) Antibiotics for acute otitis media in children. In: The Cochrane Collaboration, Sanders S, editors. Cochrane Database of Systematic Reviews. Chichester, UK: John Wiley & Sons, Ltd. Available: http://doi.wiley.com/10.1002/14651858.CD000219.pub2. Accessed 31 July 2013.

53. Rovers MM, Glasziou P, Appelman CL, Burke P, McCormick DP, et al. (2006) Antibiotics for acute otitis media: a meta-analysis with individual patient data. The Lancet 368: 1429–1435.

54. Shaikh N, Morone NE, Lopez J, et al (2007) DOes this child have a urinary tract infection? JAMA 298: 2895–2904. doi:10.1001/jama.298.24.2895.

55. Hoberman A, Chao HP, Keller DM, Hickey R, Davis HW, et al. (1993) Prevalence of urinary tract infection in febrile infants. J Pediatr 123: 17–23.

56. Krober MS, Bass JW, Powell JM, Smith FR, Seto DS (1985) Bacterial and viral pathogens causing fever in infants less than 3 months old. Am J Dis Child 1960 139: 889–892.

57. Musa-Aisien AS, Ibadin OM, Ukoh G, Akpede GO (2003) Prevalence and antimicrobial sensitivity pattern in urinary tract infection in febrile under-5s at a children's emergency unit in Nigeria. Ann Trop Paediatr 23: 39–45. doi:10.1179/000349803125002850.

58. Shaw KN, Gorelick M, McGowan KL, Yakscoe NM, Schwartz JS (1998) Prevalence of urinary tract infection in febrile young children in the emergency department. Pediatrics 102: e16.

59. Heale WF (1973) Management of urinary infections in children. Drugs 6: 230–236.

60. WHO Department of Child and Adolescent Health and Development (2005) Urinary Tract Infections in Infants and Children in Developing Countries in the Context of IMCI. Available: http://whqlibdoc.who.int/hq/2005/WHO_FCH_CAH_05.11.pdf.

61. Whiting P, Westwood M, Watt I, Cooper J, Kleijnen J (2005) Rapid tests and urine sampling techniques for the diagnosis of urinary tract infection (UTI) in children under five years: a systematic review. BMC Pediatr 5: 4. doi:10.1186/1471-2431-5-4.

62. Gorelick MH, Shaw KN (1999) Screening Tests for Urinary Tract Infection in Children: A Meta-analysis. Pediatrics 104: e54–e54.

63. Williams GJ, Macaskill P, Chan SF, Turner RM, Hodson E, et al. (2010) Absolute and relative accuracy of rapid urine tests for urinary tract infection in children: a meta-analysis. Lancet Infect Dis 10: 240–250.

64. Khan M, Coovadia YM, Connoly C, Sturm AW (1998) The early diagnosis of typhoid fever prior to the Widal test and bacteriological culture results. Acta Trop 69: 165–173.

65. Davis TM, Makepeace AE, Dallimore EA, Choo KE (1999) Relative bradycardia is not a feature of enteric fever in children. Clin Infect Dis 28: 582–586.

66. Vollaard AM, Ali S, Widjaja S, Asten HAGH van, Visser LG, et al. (2005) Identification of typhoid fever and paratyphoid fever cases at presentation in outpatient clinics in Jakarta, Indonesia. Trans R Soc Trop Med Hyg 99: 440–450. doi:10.1016/j.trstmh.2004.09.012.

67. Hosoglu S, Geyik MF, Akalin S, Ayaz C, Kokoglu OF, et al. (2006) A simple validated prediction rule to diagnose typhoid fever in Turkey. Trans R Soc Trop Med Hyg 100: 1068–1074. doi:10.1016/j.trstmh.2005.12.007.

68. Neopane A, Poudel M, Pradhan B, Dhakal R, Karki DB (2006) Enteric fever: diagnostic value of clinical features. Kathmandu Univ Med J KUMJ 4: 307–315.

69. Kuvandik C, Karaoglan I, Namiduru M, Baydar I (2009) Predictive value of clinical and laboratory findings in the diagnosis of enteric fever. New Microbiol 32: 25.

70. Open Data Kit (n.d.). Available: http://opendatakit.org/. Accessed 23 August 2013.

71. OpenMRS (n.d.). Available: http://openmrs.org/. Accessed 4 August 2013.

72. Dellinger RP, Levy MM, Carlet JM, Bion J, Parker MM, et al. (2008) Surviving Sepsis Campaign: International guidelines for management of severe sepsis and septic shock: 2008. Crit Care Med 36: 296–327. doi:10.1097/01.CCM.0000298158.12101.41.

73. Gaieski DF, Mikkelsen ME, Band RA, Pines JM, Massone R, et al. (2010) Impact of time to antibiotics on survival in patients with severe sepsis or septic shock in whom early goal-directed therapy was initiated in the emergency department*. Crit Care Med 38: 1045–1053. doi:10.1097/CCM.0b013e3181cc4824.

74. Hazir T, Nisar YB, Abbasi S, Ashraf YP, Khurshid J, et al. (2011) Comparison of oral amoxicillin with placebo for the treatment of world health organization-defined nonsevere pneumonia in children aged 2-59 months: a multicenter, double-blind, randomized, placebo-controlled trial in pakistan. Clin Infect Dis Off Publ Infect Dis Soc Am 52: 293–300. doi:10.1093/cid/ciq142.

75. Ingvarsson L (1982) Acute otalgia in children - findings and diagnosis. Acta Paediatr Scand 71: 705–710.

Figures legends:

Figure 1. Flow diagrams of study selection process in the structured literature reviews

Figure 2. Overview of ALMANACH's structure

Figure 3. Samples of ALMANACH in paper format

Figure 4. Samples of ALMANACH in electronic format

Tables

Table 1. Structured literature reviews: search strategy

	**Pubmed**	**Embase**
1	"primary health care" OR "outpatients" OR "family practice" OR "emergency service" OR "ambulatory care"	
2	"fever/etiology"[MeSH Terms] OR "fever/diagnosis"[MeSH Terms] OR "fever/epidemiology"[MeSH Terms]	
3	"developing countries"	
4	prevalence OR epidemiology OR incidence	
5	"predictive value of tests"[MeSH Terms] OR "sensitivity and specificity"[MeSH Terms] OR "reproducibility of results"[MeSH Terms] OR diagnostic test OR diagnostic tests OR "physical examination"[MeSH Terms] OR"medical history taking"[MeSH Terms]	'diagnostic accuracy'/exp OR 'predictor variable'/exp
6	"pneumonia"[MeSH Terms]	'pneumonia'/exp OR 'lower respiratory tract infection'/exp OR 'respiratory tract infection'/exp
7	“typhoid fever” [MeSH Terms]	'typhoid fever'/exp
8	“urinary tract infections”[MeSH Terms]	'urinary tract infection'/exp
9	“otitis media”[MeSH Terms]	'otitis media'/exp
10	“shigella”[MeSH Terms] OR “dysentery”[MeSH Terms]	'shigellosis'/exp
11	Filters: Infant: 1-23 months; Preschool Child: 2-5 years	'child'/exp
Prevalence	[1 AND (2 OR {3 AND 4})] AND 11	
Diagnostics	6 AND 5 AND 11	6 AND 5 AND 11
	7 AND 5 AND 11	7 AND 5 AND 11
	8 AND 5 AND 11	8 AND 5 AND 11
	9 AND 5 AND 11	9 AND 5 AND 11
	10 AND 5 AND 11	10 AND 5 AND 11

Table 2. Major changes in ALMANACH as compared to IMCI algorithm based on evidence

Location	Topic	IMCI	ALMANACH	Rationale
**Management of very severe diseases section**	**Very severe diseases**	"A child with any general danger signs needs URGENT attention; **complete the assessment** and pre-referral treatment immediately so that referral is not delayed"	If the child has any general danger sign, HWs are not asked to complete the assessment of all symptoms, but rather to "**Give pre-referral treatment and REFER URGENTLY**"	To complete the assessment would delay pre-referral treatment, and impair prognosis. In presence of general danger sign, the priority is to give rapidly presumptive AB/AM treatment[72,73] and to refer to hospital, where further etiological investigations will allow adapting the treatment.
	**List of general danger signs**	"Lethargic; Convulsing; Unable to drink/breastfeed; Vomits everything; History of convulsion"	"Convulsing; Lethargic; Unable to drink/breastfeed; Vomits everything; History of convulsion; Jaundice; Cyanosis; Stiff neck; Severe pallor; Severe wasting"	Stiff neck, severe pallor, and severe wasting (assessed later on in IMCI) are part of the ALMANACH initial assessment, in order to facilitate and fasten the detection and management of very severe diseases.<br>Jaundice and cyanosis, strong predictors for severe sepsis[15] and severe respiratory conditions have been added to the general danger signs.
	**Pre-referral treatment**	Available in the "TREAT THE CHILD" section in the middle of the booklet	Available in the "Management of very severe diseases" section in the first pages of the booklet	To facilitate and fasten the management of severe patients, the first section "Management of very severe diseases" has all assessment, classification and treatment charts together.
**Management of children with no general danger signs**	**Fever**[§]	Fever[§] is one of the 4 "Main symptoms".	Fever is a crossing point in ALMANACH: different recommendations are made for children (non-severe) with or without fever[§].	In children having no underlying chronic condition, and no danger signs, only few bacterial infections should be considered. Apart from dysentery and soft tissue infection, antibiotics are not recommended in the treatment of non-severe non-febrile conditions in ALMANACH.
	**Fever**	Classifications considered in the Fever chart are: "Very severe disease", "Malaria" and "Measles".<br>*Additional classification in low malaria risk contexts: "Fever, malaria unlikely"*	Classifications considered in the Fever algorithm: "Malaria", Acute respiratory infections, including "Pneumonia"; Diarrhea related classifications; Ear related classification; Measles; Skin infections; "UTI", "Typhoid fever", "Likely viral infection"	Designing a specific chart for patients with fever allows considering more fever related classifications than in IMCI, thus to address relevant NMF. This design allow also to consider "Likely Viral infections" after having excluded potentially life threatening conditions
**Febrile chart**	**Malaria**	Presumptive diagnosis of malaria for all children with fever in high malaria risk contexts	Test-based malaria diagnosis is recommended, using mRDTs in all children with fever. Antimalarials only recommended in test positive patients	The accuracy, the performance and the safety[7] of a diagnostic strategy based on mRDTs have been evaluated and demonstrated in U5.
**Febrile Cough chart**	**Pneumonia**	Pneumonia diagnosis rely on increased respiratory rate (RR) above age specific threshold: 50 breath/min if aged 2-11 months; 40/min if aged 12-59 months	Pneumonia is considered in children aged 2-59 months, if they report the presence of fever and have a RR above 50 breaths/min	The need of antibiotics in children aged 2-59 months with non-severe pneumonia as defined in IMCI is questioned[74]. In children aged 12-59 months the gain in sensitivity doesn't balance the loss of specificity for the diagnosis of pneumonia when using the threshold 40 instead of 50 breath/min. (see results section)
**Ear problem chart**	Acute ear infection	Oral antibiotics are recommended for "Acute ear infection" defined as either "ear pain" or "ear pus/ discharge for less than 14 days"	Oral antibiotics are only recommended for children with fever and "ear pus/ discharge for less than 14 days"	The need for antibiotics for otitis media is questioned[52]. Ear pain is a weak predictor of otitis media[49,51,75] especially in children below 2 years of age. AB are most useful for children with otitis media and ear discharge[52].
**Febrile chart for "Fever with no identified cause" after symptom charts assessment**	Urinary tract infection	Not considered in IMCI	Considered in non-severe febrile children, under 2 years of age, with no primary focus identified; and in children, above 2 years of age, with dysuria. Urinalysis using a dipstick is recommended for the diagnostic.	UTI is most frequent in children under 2 years of age. Above 2 years of age, the specificity of dysuria symptoms is low.<br>The accuracy and performance of dipstick for UTI diagnosis have been demonstrated.<br>Dipsticks for pregnancy follow-up were already broadly available in PHCFs in Tanzania; dipsticks for urinalyses were available in Health Centers.
	Typhoid fever	Not considered in IMCI	In non-severe febrile children above 2 years of age, with no primary focus identified, abdominal palpation is recommended. In presence of tenderness, a presumptive treatment for typhoid fever and invasive intestinal bacterial infections is recommended.	Typhoid fever and other invasive enteric infections are life threatening conditions. In low resource care facilities, HWs fear to miss these diagnoses and tend to overprescribe antibiotics to children with no identified causes of fever. In the Tanzanian fever study, abdominal tenderness was associated with invasive bacterial infections and typhoid[15], in children above 2 years of age.

AB: antibiotics, AM: antimalarials, HW: health worker, IMCI: Integrated Management of Childhood Illness, PHCF: primary health care facility, U5: children under 5 years of age, UTI: urinary tract infection. [§]Fever is defined by either history of fever or axillary temperature above 37.5°C or child feels hot.

Figure 1. Flow diagram of study selection
Click here to download high resolution image

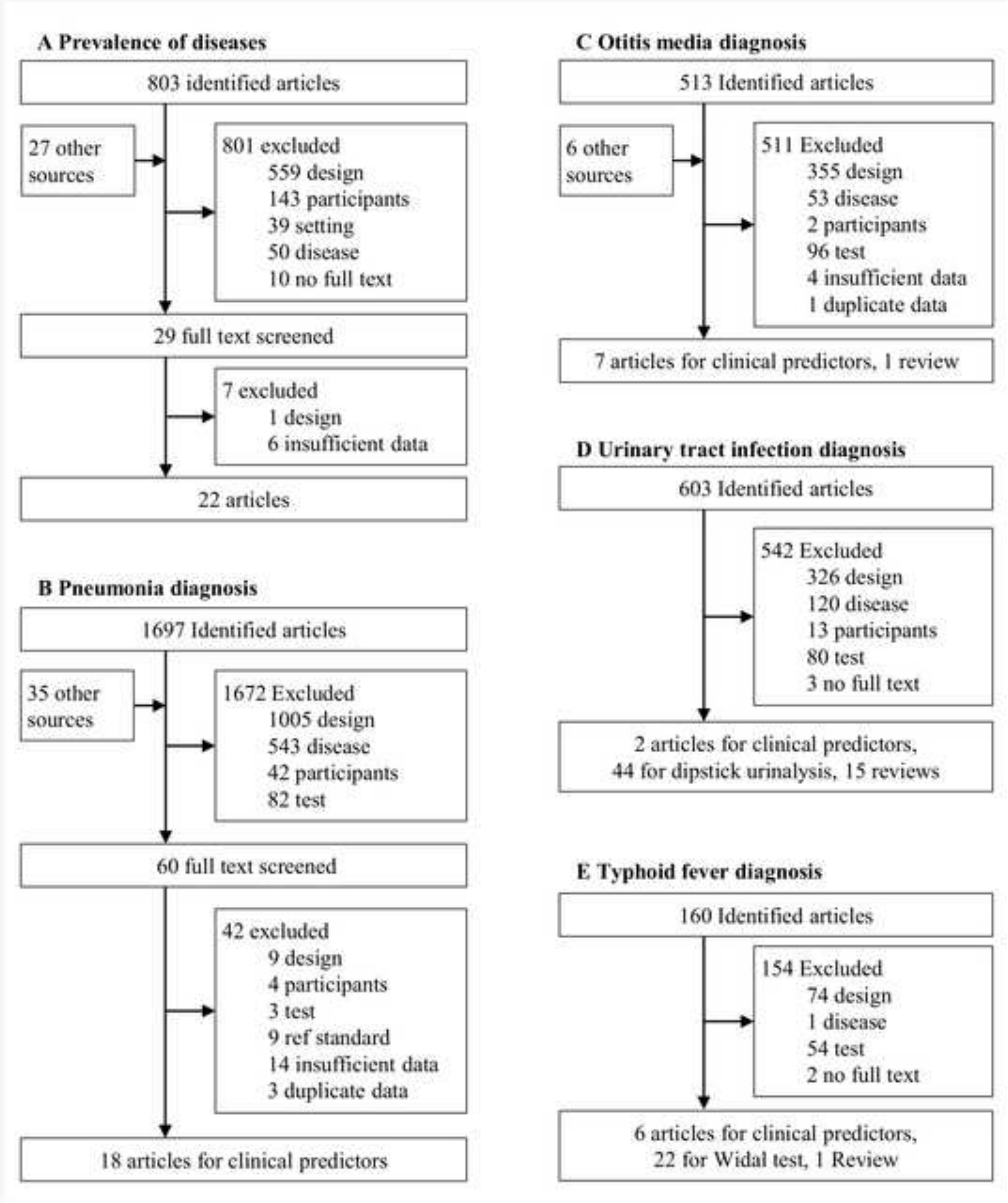

Figure 2. Overview of ALMANACH's structure
Click here to download high resolution image

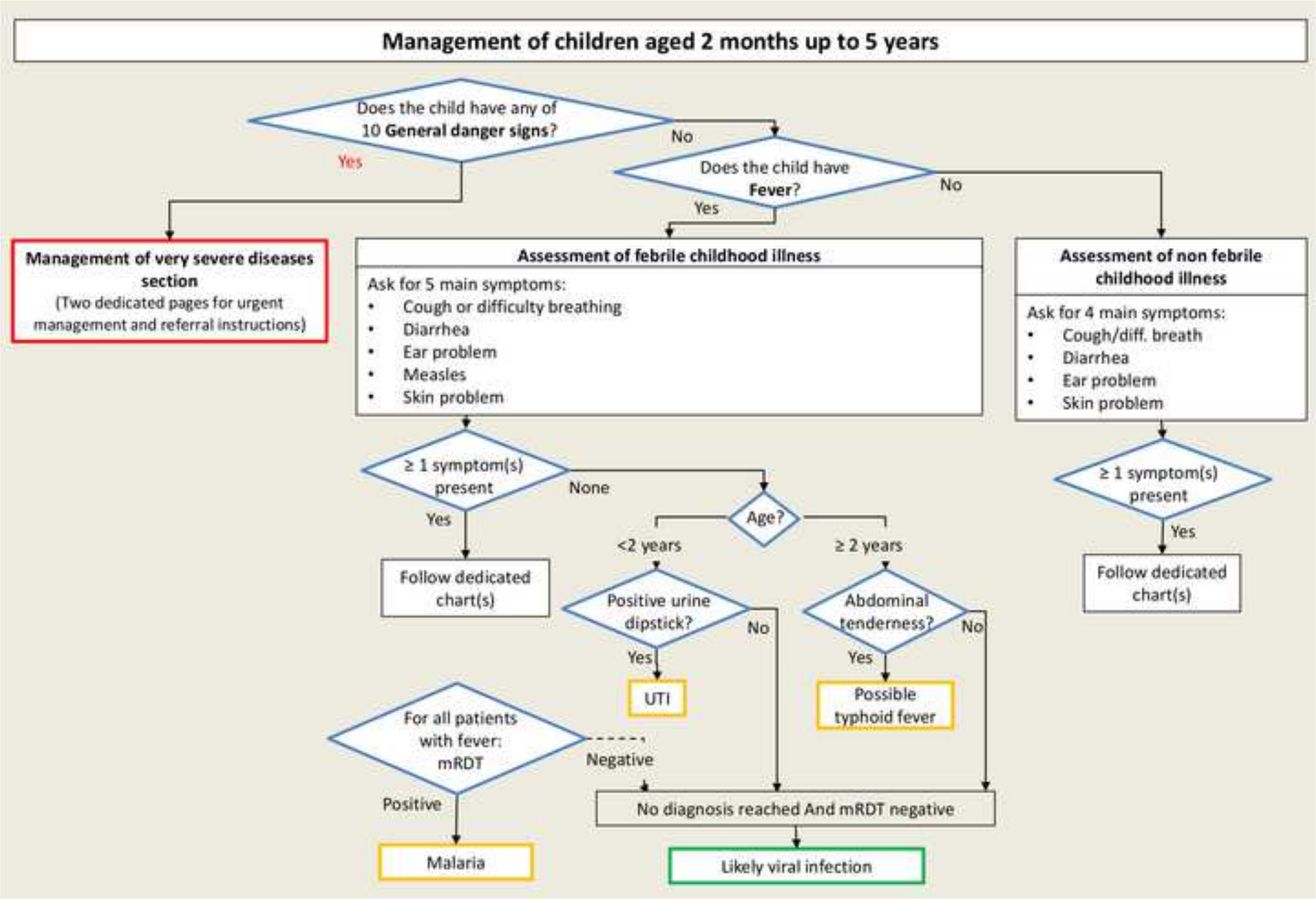

Figure 3. Sample of ALMANACH in paper format

Click here to download high resolution image

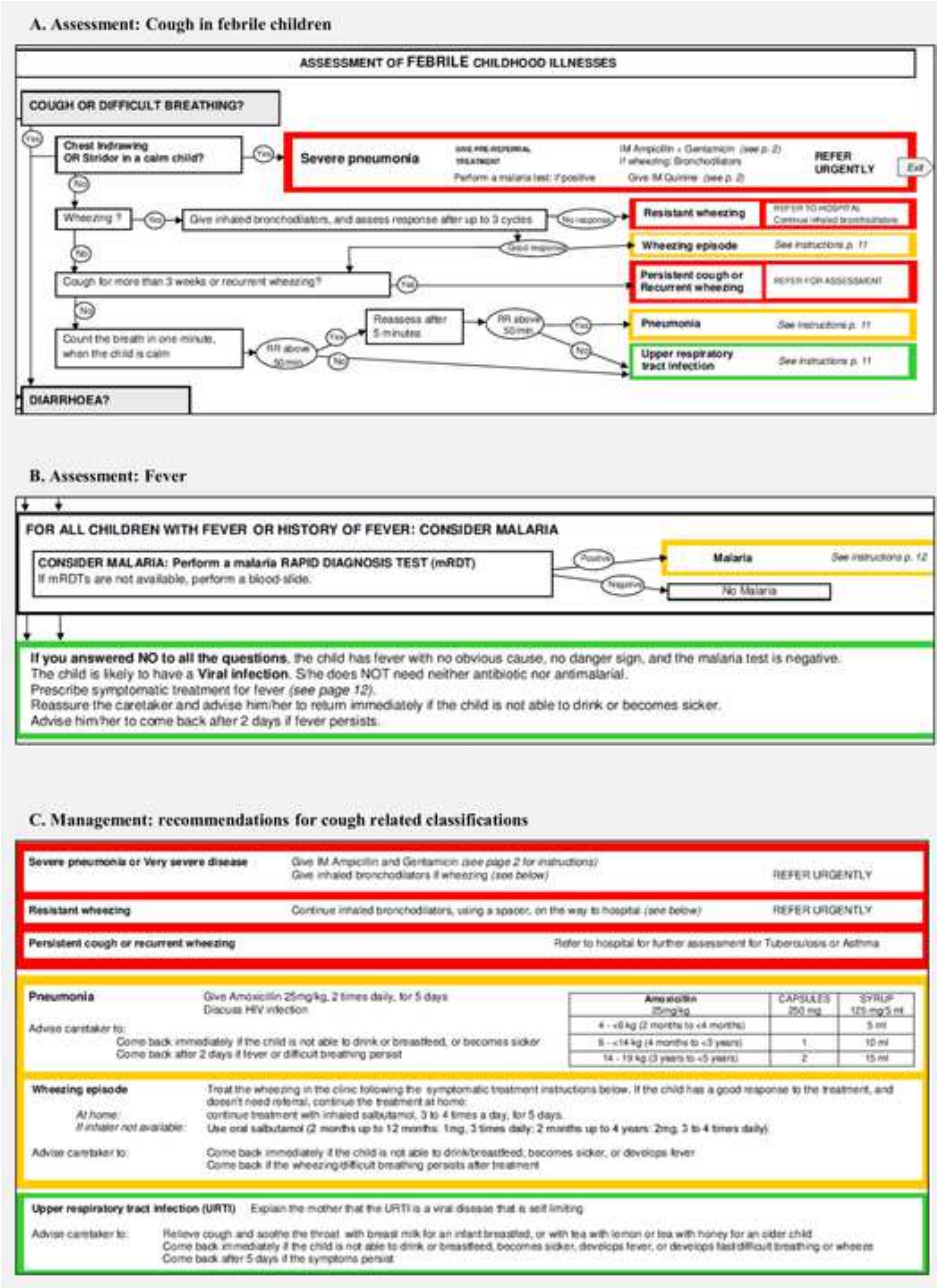

Figure 4. Samples of ALMANACH in electronic format

Click here to download high resolution image

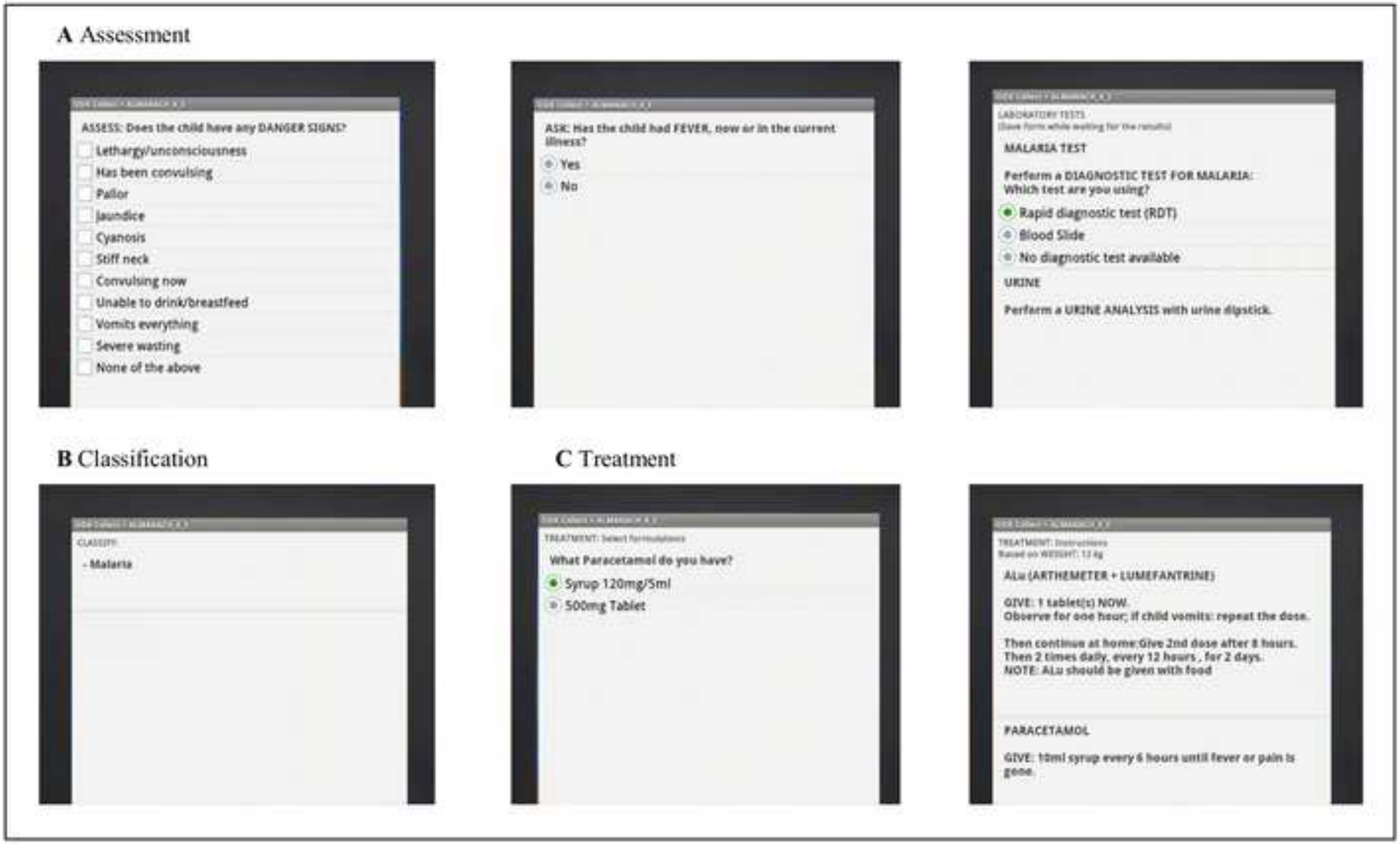

Companion paper submitted concomitantly
Click here to download Other: ALMANAC Safety paper submitted PONE-S-14-56956.pdf
