# Supplementary material for: New Algorithm for Managing Childhood Illness Using Mobile Technology (ALMANACH): A Controlled Non-Inferiority Study on Clinical Outcome and Antibiotic Use in Tanzania
Source: PLoS One. 2015 Jul 10;10(7):e0132316. doi: 10.1371/journal.pone.0132316 (PMC4498627; doi:10.1371/journal.pone.0132316)
Supplement: S2 File — (PDF) [file pone.0132316.s002.pdf]

Improving quality of health care for Tanzanian children: Assessing the use of electronic decision support to promote evidence-based medicine and rational use of drugs (PeDiAtrick)

Research Partnerships with Developing Countries
Proposal for Joint Research Project (JRP)
Call July 2008

Partnership Institutions

Switzerland
Swiss Tropical Institute [B Genton, V D'Acremont (coordinators)]
[C Rambaud, F Althaus]

Tanzania
Ifakara Health Institute (S Abdullah, Amani Shao)
DSM City Council City Medical Office of Health (J Kahama)

Tanzania & International
Non Governmental Organisation: D- Tree (M Mitchell, N Lesh)

United States
Harvard School of Public Health(M Mitchell, N Lesh, S Perri)

Version of April 19th, 2010

Partner Institutions / Organizations, Investigators

SWITERLAND

Swiss Tropical Institute was founded in 1943 as a public organisation. It is supported by the Swiss Federal Government and the Canton of Basel-Stadt. Its mandate is to contribute to the improvement of the health of populations internationally and nationally through excellence in research, services, and teaching and training. The strategies to pursue its mandate are a broad interdisciplinary approach to maintain an iterative process between the laboratory, field, bench and bed addressing by research, training and services the levels of innovation – developing concepts, methods and products, and validation – providing evidence for what works, and application - strengthening public health systems and policies. The present proposal fits therefore perfectly with theses perspectives.

Blaise Genton, MD, PhD, DTMH **, professor**, is a specialist in Tropical/Travel Medicine and Internal Medicine (FMH), project leader at the Swiss Tropical Institute and Head of the Travel Clinic at the Policlinique Médicale Universitaire, Lausanne. He has vast experience in clinical tropical medicine. His research area of expertise is evidence-based medicine, malaria epidemiology and clinical trials. He has more than 180 peer reviewed publications in the above areas.

Blaise Genton is the principal investigator and project grant recipient.

He will be the overall supervisor of the project. He will be responsible for the good conduct of the project. He will be in close contact with the implementers (Dr Clotilde Rambaud, Dr Amani Shao and Dr Judy Kahama-Maro) and take all strategic decisions for the field implementation. He will be guiding for the data analysis and involved in study finding dissemination. He is also the guarantor of the academic degrees (PhD) of Drs C Rambaud and A. Shao.

Valérie D'Acremont, MD, MiH, DTMH is a specialist in Infectious Diseases (FMH), research fellow at the Swiss Tropical Institute. She has vast experience in clinical infectious diseases. She is specialized in guidelines development and implementation, as well as evaluation. She worked in Tanzania (2006-2009) to conduct a project aimed at implementing and evaluating the malaria rapid diagnostic tests. She concurrently run a large study to investigate the etiology of fever in children. This study should provide further evidence to revise the diagnostic and treatment algorithm.

Valérie D'Acremont will be a supervisor of the project.

Her main tasks will be to supervise the development of an improved IMCI decision chart after analysis of the study on etiology of fevers, to supervise the cluster randomized studies in Dar es Salaam and Ifakara, from its implementation in the field to the data analysis and manuscripts writing.

Clotilde Rambaud Althaus, MD is a specialist in General Internal Medicine, Postgraduate in Tropical Medicine and International Health of the Tropical School of Antwerp, Belgium, research physician at the Swiss Tropical and Public Health Institute.

Clotilde Rambaud will conduct the PeDiAtrick project and perform a PhD jointly with Dr Amani Shao.

Her main tasks will be to develop and improve the IMCI decision chart after analysis of the fever etiologies study with Dr Valérie D'Acremont, to develop and organize the new IMCI training, to develop the data collection tools, to conduct in the field the cluster randomized studies in Dar es salaam and Ifakara, with a special focus on the assessment of the health worker adherence to IMCI charts, to build database and supervise data entry, to perform data cleaning and analysis, to write manuscripts, to follow up the expenses and budget reports done by IHI and to write interim and final reports.

Fabrice Althaus, MD is a specialist in General Internal Medicine (FMH), Postgraduate in Tropical Medicine and International Health of the Tropical School of Antwerp, Belgium. He has a 10 years clinical experience in European and African settings and conducted several studies as first author or co-investigator in the field of migrants' health, vulnerable populations, and cross-cultural education.

Fabrice Althaus will be a consultant and research assistant.

His main tasks will be to assist the investigators in the implementation of the studies in the field, to facilitate literature reviews, to help for data management & analysis and assist in the manuscript writing.

TANZANIA

Ifakara Health Institute **(IHI)** (formally Ifakara Health research and Development Center) will be a collaborating partner in this project and facilitate much of the logistics and work with the clinics. IHI was founded in 1957 as the Swiss Tropical Institute Field Laboratory. In 1996 an independent, non-profit foundation was constituted and registered in Tanzania as IHI Trust. Since 1997 the Centre has operated as a Trust under the leadership of the Ministry of Health. For the purpose of this project, IHI will hire staff (or use existing staff) to manage the logistics of this project and clinicians to oversee the use of e-IMCI at health facilities.

Salim Abdulla, MD PhD, director of the Ifakara Health Institute, has a long-standing experience in clinical research in Tanzania. He acknowledges the importance of improving the quality of care of children in Tanzania through the use of novel technologies. The IHI has embarked on several projects using PDAs or new electronic devices and the director sees a complementarity of the present project with other activities of this kind.

Salim will oversee and coordinate the IHI activities linked with the project and oversee the IHI budget management and reports.

Amani Shao, MD, MPH is a general practitioner and public health specialist graduate from Harvard School of Public Health, Boston, USA. He has been working as research scientist for the National Institute for Medical Research in two research sites with different malaria transmission intensities and he has 7 years experience in clinical and research in malaria diagnosis and treatment in children attending outpatient and inpatient services in Tanzania.

Amani Shao will conduct the PeDiAtrick project and perform a PhD jointly with Dr Clotilde Rambaud.

His main tasks will be to develop and organize the new IMCI training, to ensure links with Harvard School of Public Health and D-tree for the development of the electronic IMCI decision chart, to develop the data collection tools, to conduct in the field the cluster randomized studies in Dar es salaam and Ifakara, with a special focus on the assessment of the safety of the interventions and on the cost analysis of the project, to build database and supervise data entry, to perform data cleaning and analysis, to write manuscripts and to write interim and final reports.

DSM City Council City Medical Office of Health

The Swiss Tropical institute has been working with the DSM CMOH within the so-called 'Dar es Salaam Urban Health Project' and more recently in the 'IMALDIA' project, both projects funded for a large part by Swiss and both aimed at decreasing the burden of malaria in urban Dar es Salaam. This collaboration has been very fructuous and has led to a considerable impact on both malaria transmission and malaria case management.

Judy Kahama, MD, MPH is the City Medical Officer for Dar es Salaam and co-investigator. She has been one of the two main players in the previous project of implementation and evaluation of RDT for malaria in Dar es Salaam and Kilombero /Ulanga districts. She has been recently appointed City Medical Officer for Dar es Salaam, due to her recognized competence in the field of public health and excellent communication skills with all partners at the MoH level and the players in the health system. Thanks to her competence, she will be of great value for the successful conduct of this project.

Judith Kahama will facilitate and supervise the implementation of the study in the Health Facilities in Dar es Salaam. She will ensure links with the National, Regional and District IMCI actors.

TANZANIA AND INTERNATIONAL (USA)

D-Tree International

D-tree, International was established in 2004 as a non-profit organization dedicated to improving the quality of health care available to the world's poor by using hand held technology to provide accurate and effective point-of-care diagnosis and treatment. Our vision is to equip frontline health workers with the tools they need to deliver first-class evidence-based medical care in Africa, Asia and throughout the world. Our work to date has focused on designing the protocols and a user interface that enable providers to quickly learn how to navigate the PDA and achieve accurate recording of patient responses and interpretation of results. In Tanzania we have adapted the widely used IMCI protocols for use on a mobile device, testing protocols to screen AIDS patients on ARV treatment for serious medical problems, resulting from their disease or treatment and developing and testing CommCare for use by community health workers for the Millennium Village Project.

Harvard School of Public Health (HSPH) brings together a tradition of scholarship, research, and service in the field of international health, including a commitment to work towards the improvement of health in developing countries. In addition to the work of individual faculty and senior staff, the School of Public Health maintains working relationships with senior health officials in countries and international organizations around the world—many of whom were trained at Harvard. The current student body represents some 50 countries, reflecting a school-wide interest in international health activities. HSPH supports many research projects including several related to the use of mobile technology to improve health care delivery. These projects have included a project in South Africa to screen AIDs patients for treatment and another in the area of IMCI.

Marc Mitchell, MD, MS is president of D-Tree, Inc as well a Lecturer on International Health at Harvard University School of Public Health where his areas of expertise include health systems design, management, and evaluation. Dr. Mitchell is a pediatrician and management specialist and has worked in over 35 countries in Africa, Asia, and Latin America on the design and delivery of health care services. He first worked in Tanzania in 1977.

Marc Mitchell will be a supervisor of the project.

His main tasks will be to be responsible for the electronic development of the IMCI tool, to supervise the development of communication messages for the health workers, to supervise the evaluation of caretaker understanding and action taken at home, and the cost analysis.

Neal Lesh, PhD, MPH is a computer-human interaction specialist who has researched and published extensively in a variety of areas including planning, intent inference, information visualization, interactive optimization, intelligent tutoring, agent interfaces, and human-computer collaboration. As a Senior Scientist at the Mitsubishi Electric Research Laboratory (MERL) in Boston, he managed and collaborated on projects exploring new interfaces to allow humans and computers to cooperatively solve problems. He is chief technology officer of D-tree International.

Neal Lesh main task in this project will be to ensure the development of the electronic IMCI decision chart, to follow up its implementation in the field and to supervise the automatic data collection and reporting systems and server.

Seneca Perri, BSN, is a registered nurse and PhD candidate in Nursing Informatics from the University of Utah. She brings a mix of clinical nursing and ICT experience, having worked in neonatal intensive care in the US as well as pediatric care in India and Thailand.

Seneca Perri will be responsible for supporting the implementation of the e-IMCI tool in the clinics. She will also organize and oversee the training on communications modules and on the electronic device, and participate on the implementation of the cluster randomized studies in Dar es Salaam and Ifakara.

Abbreviations

ARI: Acute Respiratory Infections
BG: Dr Blaise Genton
CMOH: DSM City Council City Medical Office of Health
HM : Dr Hassan Mshinda
IHI: Ifakara Health Institue (formally Ifakara Health Research and Development Center)
IMCI: Integrated Management of Childhood Illness
JK: Dr Judy Kahama
K/U: Kilombero / Ulanga districts
MM: Dr Marc Mitchell
PDA: Personal Digital Assistant
RDT: Rapid Diagnostic Test for malaria
SSA: Sub-Saharan Africa
STI: Swiss Tropical Institute
VDA: Dr Valérie D'Acremont
WHO: World Health Organization

1 Summary

Background

Nearly 10 million children die each year before the age of 5 despite the fact that effective low-cost interventions are available. Integrated Management of Childhood Illness (IMCI) treatment algorithms have proved, when used correctly, to improve quality of care, reduce the cost of treatment, and reduce under-5 mortality. However, the actual impact of IMCI worldwide has been less than anticipated due to limited uptake of the intervention, and poor adherence to algorithms by clinicians. A recent study in Tanzania showed poor adherence to the IMCI protocols, resulting to low quality of care. Indeed 99% of the children attending outpatient clinics with fever who tested negative for malaria were prescribed antibiotics, although this was appropriate in less than 20%. Such procedures lead to poor health outcomes, huge wastage of drugs and rapid spread of bacterial resistance.

The rapidly changing patterns of disease and drug resistance of microorganisms, the poor adherence to clinical guidelines using paper based algorithms and the increasing availability of new technologies for more accurate diagnostics and better adherence to evidence based clinical algorithms are the basis of this project. We will test whether the technologies that are available today can be used to improve the quality of care that is being provided to Tanzanian children through the use of electronic decision support to promote evidence-based medicine and the use of rapid diagnostic tests which promote the rational use of drugs. We will also test whether the use of electronic IMCI protocols can improve the way clinicians communicate with mothers to ensure that mothers understand the diagnosis and treatment plan of the child being seen.

In particular, we expect to show that it is feasible and cost effective to

- Revise standardized diagnostic and treatment procedures for the management of childhood illness (IMCI) incorporating more sophisticated clinical algorithms based on current evidence about disease patterns and drug resistance;
- Incorporate guides for health workers to improve communication about diagnosis and treatment to mother or other caretaker;
- Rapidly incorporate these changes into case management algorithms in an electronic format that is accurately used;
- Demonstrate improved clinical and cost effectiveness of electronic algorithms when compared to the used of paper based IMCI algorithms leading to a reduction in the inappropriate use of antibiotics and antimalarials for children with fever, and therefore improve the quality of care that is being provided to children in Tanzania.

Methodology:

Phase 1: Preparatory phase (6 months). Development of improved IMCI guidelines including RDT in the overall strategy, as endorsed by NMCP and included in the Round 7 Global Fund proposal. The improved IMCI guidelines will be following current WHO amendments regarding fever management and integration of RDT. All training material, study instruments and proposed caretaker messages will be developed during this phase.

Phase 2: Study Phase (24 months) will be a randomized control study of the implementation and use of IMCI protocols for the assessment and treatment of children 2 months-5 years of age with fever. It will compare the results of 3 treatment arms (health facility being the randomisation unit):

a) training and use of new IMCI algorithms for fever using paper decision charts
b) training and use of new IMCI algorithms for fever using electronic algorithms
c) control: old IMCI algorithms on paper charts (or usual procedures)

Phase 3: Analysis and writing (6 months)

Expected outcome

We expect to show that the use of decision support systems using mobile technology will enhance the ability to improve and rapidly implement evidence based clinical algorithms by improving adherence of health workers to these improved algorithms, leading to better health outcomes and more rational

use of antimalarial drugs and antibiotics, and better mothers' understanding of the diagnosis and treatment plan, when compared to paper-based decision charts or usual health facility procedures.

2 RESEARCH PLAN

2.1 Introduction and Literature review and statement of the problem

2.1.1 Low adherence of clinicians to evidence-based recommendation for the management of childhood illnesses

Nearly 10 million children die each year before reaching the age of five (Black 2003) despite the fact that effective low-cost interventions are available that could prevent two-thirds of these deaths (The Bellagio Study Group on Child Survival). One reason for the continuing high rates of death is the poor quality of medical care that many children receive due to shortages of health personnel and the limited training that is often available in child health. In response to this toll, the World Health Organization WHO) has developed the Integrated Management of Childhood Illness (IMCI) treatment algorithms which consist of an evidence based approach to the assessment, classification and treatment of the most common causes of childhood mortality. The impact of the effective use of IMCI has been well documented in a series of multi-country evaluations done by the WHO (Bryce 2004, 2005) that conclude that IMCI has the potential to improve quality of care, reduce the cost of treatment, and reduce under-5 mortality when used correctly. This impact has also been shown in Tanzania (Tanzania IMCI multi-country evaluation 2004).

However, there is a growing body of evidence that the actual impact of IMCI worldwide has been less than anticipated due to limited uptake of the intervention, especially among the world's most poor. (Victora 2006). There are several reasons for this including the following:

- In Tanzania and elsewhere **health worker adherence to algorithms is uneven and often quite low**, both in rural and urban settings (Tanzania IMCI multi-country evaluation 2004; VDA & JK, unpublished). A study in Papua New Guinea, where some of the earliest algorithms were developed, found that despite a correct diagnosis of malaria, and the existence of paper-based algorithms, only 11% of community health workers used the correct treatment (Beracochea 1995).
- The cost and time for training in these use of algorithms (11-16 days of initial provider training is required) has limited their uptake worldwide. Further, this has led to a **limited ability to revise algorithms as disease patterns and drug resistance change** since retraining and reprinting of all materials are required. This means that outdated algorithms are often used well beyond the need for change. This is particularly detrimental when these are changes in drug resistance or disease patterns.
- The current practice of IMCI algorithms requires health workers to follow flow diagrams, understand and use accessory information on each page, and accurately flip through up to 8-10 pages of the chart book in front of the patient. In actual practice, **most health workers decide not to use the charts** due to the embarrassment of using a chart booklet in front of patients.
- Even when the IMCI algorithms are correctly followed by the health worker there is evidence that the mother may not fully understand the diagnosis and the specific instructions for treatment and follow up at home. Unless the mother is able to give the child the correct treatment at home, the full impact of IMCI will not be achieved.

2.1.2 Changes in disease patterns and drug resistance.

During the past 20 years since the first IMCI protocols were developed, patterns of disease and drug resistance has changed dramatically and the protocols have not kept pace with these changes.

As an example, malaria among febrile children in Dar es Salaam has gone down dramatically during this period due to improvements in sanitation and housing and the wider use of insecticide treated bed nets. Further during this period, the drug of choice for treatment has gone from chloroquine which was inexpensive and relatively safe to artemether/lumefantrine (ALU) and quinine which are relatively expensive and have many side effects for the latter. Despite the evidence that only 10% of children with fever in Dar es Salaam have malaria, all these children are still systematically treated for malaria at great expense and with the risk of not being treated for other potentially fatal diseases (Kahama 2008). This evolving epidemiology definitely calls for revision of IMCI (D'Acremont, submitted). The same consideration is needed in relation to the dynamic of the HIV epidemic in young children.

For bacterial infections, there are similar changes. The global problem of antibiotic resistance is particularly pressing in developing countries, where the infectious disease burden is high and cost constraints prevent the widespread application of newer, more expensive agents (reviewed in Okeke 2005a). In Tanzania, there is recent evidence that common microorganisms are multi-resistant to drugs recommended as first line treatment. For example, in 2007, all *Shigella* strains isolated from diarrhoea patients showed high level of resistance to ampicillin, tetracycline, co-trimoxazole and chloramphenicol; moreover, 5% and 2% were also resistant to amoxycilline-clavulanate and azithromycin (Temu 2007). Resistance of bacteria causing ARI has not been extensively studied. In a carriage study published in 2003, *S. pneumoniae* showed a level of resistance to co-trimoxazole of 47% and to penicillin of 21% (Batt 2003). One half of community-acquired *Salmonellae* were resistant to ampicillin and co-trimoxazole, the currently prescribed treatment in Tanzania. Although the correlation between reduced antibiotic susceptibility *in vitro* and clinical response is not always straightforward, recent data from Tanzania show that bacterial resistance has indeed clinical implications since children infected with resistant microorganisms were more likely to die (Blomberg 2007).

One reason for this increase in antibiotic resistance is the wide inappropriate use of antibiotics in clinical practice (Hart 1998). Surveys on antibiotic use in developing countries show antibiotics prescribed in 35 to 60% of clinical encounters although appropriate in less than 20% (Trostle, 1996). In Tanzania a recent study recorded that 99% of children with fever attending the outpatient clinic and testing negative for malaria were given antibiotics, and this mainly in relation to acute respiratory infections (ARI) (VDA & JK, unpublished). Although we know that the inappropriate use of antibiotics by health workers has led to dangerous widespread drug resistance, the different approaches used to reverse this trend have been largely ineffective. In a systematic review of the effectiveness of all possible intervention to promote rational use of antibiotics (WHO 2001), educational/training interventions successfully improved targeted antibiotic prescribing outcomes by only 20% and even these changes were not necessarily sustainable over time. A holistic strategy is needed to contain antibiotic resistance, and improvement of clinician adherence to evidence-based guidelines is one proposed component (Okeke 2005b)

2.1.3 Availability of new technologies for accurate diagnosis and treatment

Two recent technologies have shown significant promise in improving both diagnosis and treatment of children with fever. One is the introduction of Rapid Diagnostic Tests (RDT) for Malaria which demonstrated the ability to accurately diagnose malaria, both in non-immunes (Marx 2005) and semi-immune populations (Ochola 2005), as well as in urban and rural Tanzania (Kahama 2008).

A second technology is the development and use of hand held electronic decision support that makes it easier and faster for health workers to accurately follow electronic IMCI (e-IMCI) algorithms in a clinical setting (Mitchell 2008). Guiding health workers step-by-step through the assessment, classification (diagnosis) and treatment of sick children in rural clinics in Tanzania using PDA has showed that e-IMCI has the potential to improve adherence to procedures, and thus the quality of care (DeRenzi 2008). Training time for e-IMCI was 20 minutes, after which clinicians were easily able to follow even complex algorithms. The clinicians unanimously preferred e-IMCI to following the paper chart booklet, citing it as faster and easier to use. This same technology is being tested for other

diagnostic areas such as the screening of HIV + patients on treatment with ART for infections or side effects of treatment.

In addition to these two technologies, other diagnostic tests and decision support algorithms are being developed rapidly with the likelihood that in 1-2 years time rapid diagnostics for many causes of both childhood and adult illness will be available. Incorporating these new technologies into electronic decision support algorithms will be much easier than into paper charts (see for example Bill and Melinda Gates Foundation Grand Challenges *http://www.gcgh.org/Pages/BrowseByTechnology.aspx#Diagnostics*).

2.2 Past performance on the research field carried out by the applicants

2.2.1 Evidence-based medicine (BG, VDA, MM)

Drs BG & VDA have extensive experience in the field of evidence-based medicine and it is their *credo* that promoting an evidence-based approach is one of the only ways to improve global health at reasonable cost.

For more than 15 years, we have been conducting prospective studies to identify clinical and laboratory predictors of infectious diseases such as malaria (Genton 1994; D'Acremont 2002), typhoid fever and other causes of fever in children in Africa (VDA, BG, unpublished). At the same time, we conducted several systematic reviews and metanalyses in the field of malaria diagnosis (Marx 2005), proportion of fevers due to malaria in Sub-Saharan Africa (SSA) (D'Acremont submitted) and fever in travelers and migrants (D'Acremont 2003). This gave us extensive expertise in the development of decision charts for the diagnosis and management of fever, both in developed countries and in the tropics. We developed practice guidelines that were endorsed by international societies (D'Acremont et al. 2003). We also developed an electronic version of practice guidelines for the use of antibiotic in hospitals and are constantly promoting clear messages for the rational use of antimalarials and antibiotics (D'Acremont 2007; Genton 2007).

The IHI and its directorship has been heavily involved in the evaluation of the IMCI strategy in Tanzania. The IHI, of which Salim Abdullah is the director, has been at the forefront of all impact assessments of IMCI on quality of care, under-5 mortality, cost-effectiveness and equity (Amstrong-Schellenberg 2004, Schellenberg 2003, Bryce 2006, Masanja 2008). He is strongly supporting the new studies assessing use of PDAs for research, clinical and data collection purposes (Shirima 2007).

Dr MM began his work on the development of treatment protocols in 1986 in Pakistan. Working together with the Directors of Health from each of the four Provinces, he developed protocols linked to a data collection and monitoring system for childhood symptoms that were the most common presenting complaints at the primary health care level. This work led to a national program that was implemented through a USAID grant to Pakistan and was one of the earliest successful uses of protocols in such a setting. Since then he has continued in the development and use of clinical standards to improve quality of care in developing countries.

For these achievements, all partners involved are recognized in the field of evidence-based medicine. We feel confident to be able to appropriately revise the decision charts for the management of fever in children in SSA (IMCI-derived). Also, VDA together with JK have already designed, as well as successfully implemented and evaluated an amended IMCI algorithm, which included for the first time a laboratory test in the same geographical areas that have been selected for the present proposal (D'Acremont 2008b). This work was coupled with a large study on etiologies of fever including 1000 children aged 2 months to 10 years (500 in urban Dar es Salaam and 500 in rural Ifakara) where modern diagnostic technologies are used to document as best as possible the causative agent. This study should provide a significant body of evidence for the revision of the IMCI guidelines.

More recently, we have gone beyond clinical research or practice guidelines development, and entered the field of evidence-based policy development and promotion. Indeed, we are involved in several boards that are reviewing the dogma of presumptive treatment with antimalarials for all children

under five years with fever in malaria endemic areas, arguing that it is time to consider other causes of fever, also potentially fatal when left untreated (D'Acremont 2008).

2.2.2 Impact evaluation of new diagnostic tools to promote rationale use of drugs for the management of fever in children in Tanzania (JK, HM, VDA, BG)

The diagnosis of malaria remains a critical issue for improving the management of fever in endemic areas. We have pioneered the field assessment of the first generation Histidin-Rich Protein-2 Rapid Diagnostic Tests (RDTs) for malaria in rural health facilities in endemic areas (Genton 1996, 1998, 2000), and more recently of the lactate dehydrogenase tests (Mueller 2005). Following the demonstration in a systematic review that RDTs performed as well (Marx 2005), or even better than microscopy (Ochola 2006), we designed a project aimed at implementing and evaluating the impact of the introduction of RDTs for the management of fever in children in areas of Tanzania. We could demonstrate a dramatic decline of antimalarial prescription in most health facilities where this intervention has been applied, which did not happen in control health facilities (D'Acremont 2008a). Likewise, the prescription of antibiotics increased significantly, since the clinicians were worried about septicaemia, especially typhoid fever. This outcome forms the basis for the present proposal. We also developed new tools for the evaluation and monitoring of malaria parasite to standard antimalarials. We devised a microarray that is able to identify molecular markers of resistance and tested it in the field in Papua New Guinea, Cambodia and Tanzania. The technology transfer has been done in the last two countries as part of our long-standing collaboration and the research institutes in endemic areas are now able to monitor themselves the dynamic of parasite resistance and provide up-to-date epidemiological data to the national Malaria Control Programme for policy adjustment. This is another example of successful partnership between the institutions involved in the present proposal and a proof that sophisticated technologies can also be applied in countries with limited resources.

2.2.3 Development and evaluation of computerized decision support system (MM, HM, VDA, BG)

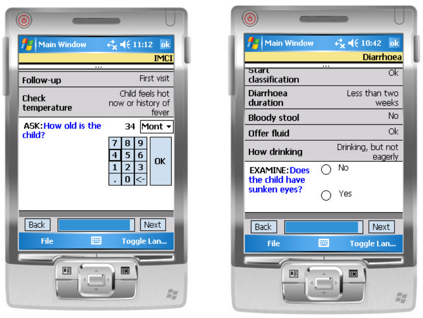

Figure 1: The e-IMCI interface.

D-tree International (MM) has developed and piloted and electronic IMCI (e-IMCI) running on a PDA in 2007 together with IHI. During this initial pilot study, nurses and clinical officers in Tanzania used the electronic to see children who came to the clinic for an acute care visit for one of the problems covered in IMCI. Our pilot results found that e-IMCI improved adherence to IMCI protocols in a sample of 52 patients. In particular we found that protocols were followed in 61% of investigations for paper based IMCI vs. 84.7% for e-IMCI ($p < 0.01$). e-IMCI did not take much more time than paper based IMCI. Further, all the clinicians we have worked with were enthusiastic about e-IMCI and learned how to use it in less than 1 hour.

D-tree International is currently conducting a second study of electronic IMCI together with the IHI. This study is designed to rigorously evaluate the use of e-IMCI by health care providers in terms of feasibility and time spent for the consultation.

This e-IMCI system was built from a related system we have tested in AIDS treatment centres in South Africa evaluating whether lay counsellors using IMAI-based screening protocols on a PDA could accurately determine which patients need referral to a physician during routine HIV treatment visits.

In a related set of studies, VDA & BG developed practice guidelines for the management of febrile patients returning from the tropics We constructed a consultation website *www.fevertravel.ch* that comprises a decision chart and specific diagnostic features providing medical diagnostic assistance to primary care physicians (for details see Ambresin et al 2006). We then integrated an evaluation component aimed at assessing feasibility of implementing guidelines over the internet and adherence

of the target audience (primary care physicians) to the proposed attitude. Outcomes measures included level of adherence of the joining primary care physicians to the recommendations, reasons for non-adherence, discrepancy between diagnoses proposed by the website and final diagnoses, satisfaction of the website users. More than 600 primary care physicians/patients pairs have been included. Preliminary results show that the guidelines are safe and that more than 50% of physicians fully adhere to the recommendations (Müller et al, in preparation). This is an example of electronic algorithms for the management of fever using internet technology including an in-built assessment component. The wide expertise gained in this project will be of great help for the development and evaluation of the PDA tool as described in the present proposal.

Similarly, we developed a computerized decision support system to help for the rational use of antibiotics in hospitals, with again an in-built evaluation component.

2.2.4 References for section 2.2

Ambresin AE, D'Acremont V, Mueller Y, Martin O, Burnand B, Genton B. www.fevertravel.ch: an online study prototype to evaluate the safety and feasibility of computerized guidelines for fever in returning travellers and migrants. *Computer Methods and Programs in Biomedicine* 2007; 85: 19-31.

Armstrong Schellenberg, JR, Adam T. Msinda, H, et. at. 2004, Effectiveness and cost of facility-based IMCI in Tanzania. *The Lancet* 364: 1583-94.

Batt SL, Charalambous BM, Solomon AW et al. Impact of azithromycin administration for trachoma control on the carriage of antibiotic-resistant Streptococcus pneumoniae. Antimicrob Agents Chemother. 2003 Sep;47(9):2765-9.

Beracochea E, Dickson R, Freeman P, Thomason J. Case management quality assessment in rural areas of Papua New Guinea. Trop Doct. 1995, 25(2):69-74.

Black RE, Morris SS, Bryce J, Where and why are 10 million children dying every year? The Lancet, Vol 361,June 28, 2003.

Blomberg B, Manji KP, Urassa WK et al. Antimicrobial resistance predicts death in Tanzanian children with bloodstream infections: a prospective cohort study. BMC Infect Dis. 2007 May 22;7:43.

Bryce J, El Arifeen S, Bhutta ZA et al. Getting it right for children: a review of UNICEF joint health and nutrition strategy for 2006-15. Lancet. 2006 ;368(9538):817-9.

Bryce J, Gouws E, Adam T, Black RE, Schellenberg JA, Manzi F, Victora CG, Habicht JP. "Improving quality and efficiency of facility-based child health care through Integrated Management of Childhood Illness in Tanzania." Health Policy and Planning. 2005 Dec; 20 Supplement 1: i69-i76.

Bryce J, Victora CG, Habicht JP, Black RE, Scherpbier RW; MCE-IMCI Technical Advisors "Programmatic pathways to child survival: results of a multi-country evaluation of Integrated Management of Childhood Illness." Health Policy and Planning. 2005 Supplement 1: i5-i17.

Bryce J, Victora CG, Habicht JP, Vaughan JP, Black RE. "The multi-country evaluation of the integrated management of childhood illness strategy: lessons for the evaluation of public health interventions." American Journal of Public Health. 2004 Mar; 94 406-15.

Bryce J, Victora CG; MCE-IMCI Technical Advisors. "Ten methodological lessons from the multi-country evaluation of integrated Management of Childhood Illness." Health Policy and Planning. 2005 Supplement 1: i94-i105.

D'Acremont V, Landry P, Müller I, Pécoud A, Genton B. Clinical and laboratory predictors of imported malaria : an aid to the medical decision making for returning travelers with fever. *Am J Trop Med Hyg* 2002;66:481-6.

D'Acremont V, Ambresin AE, Burnand B, Genton B. Practice Guidelines for fever in returning travelers and migrants. *Journal of Travel Medicine* 2003; 10 Supplement 2: S25-S52.

D'Acremont V, Lengeler C, Genton B. Stop ambiguous messages on malaria diagnosis. *British Medical Journal*, 2007 ;334 :489.

D'Acremont V, Lengeler C, Mshinda H, Mtasiwa D, Tanner M, Genton B. Overdiagnosis of malaria in Sub-Saharan Africa: time to review the policy of presumptive antimalarial treatment of underfive children with fever. Submitted to *PloS Medicine*.

D'Acremont V, Kahama-Maro J, Mtasiwa D, Genton B, Lengeler C. Massive reduction of antimalarial prescriptions after Rapid Diagnostic Tests implementation in Dar es Salaam, Tanzania. Abstract in Am J Trop Med Hyg Suppl of the 57th Annual Meeting of the American Society of Tropical Medicine and Hygiene, New Orleans, USA, 7-11 December 2008a.

D'Acremont V, Kahama-Maro J, Mtasiwa D, Lengeler C, B. Genton. Withdrawing antimalarials in

febrile children with a negative Rapid Diagnostic Test is safe in a moderately endemic area of Tanzania. Abstract in Am J Trop Med Hyg Suppl of the 57th Annual Meeting of the American Society of Tropical Medicine and Hygiene, New Orleans, USA, 7-11 December 2008b.

DeRenzi, Brian, Neal Lesh, Tapan Parikh, Clayton Sims, Werner Maokola, David Schellenberg, , Gaetano Borriello, Marc Mitchell,e-IMCI: Improving Pediatric Health Care in Low-Income Countries (CHI conference, 2008) in press

Genton B, Smith T, Baea et al. Malaria: how useful are clinical criteria for improving the diagnosis among presumptive cases in a highly endemic area? *Transactions of the Royal Society of Tropical Medicine and Hygiene* 1994;88:537-541.

Genton B, Hii J, Paget S, Alpers M. Rapid manual diagnosis of *Plasmodium falciparum* malaria using P*ara*Sight[R]-F dipsticks applied to human blood and urine. *Journal of Travel Medicine* 1996;3;172-173.

Genton B, Paget S, Beck H-P, Gibson N, Alpers MP, Hii J. Diagnosis of *Plasmodium falciparum* infection using the *Para*Sight[R]-F test in blood and urine of Papua New Guinean children. *Southeast Asian Journal of Tropical Medicine and Public Health* 1998;29:35-40.

Genton B, D'Acremont. Rapid diagnostic tests for malaria in returning travellers (electronic letter). *British Medical Journal* 2000, http://www.bmj.com/cgi/eletters/321/7259/484#EL2.

Genton B, D'Acremont V. Evidence of efficacy is not enough to develop recommendations: antibiotics for treatment of traveler's diarrhea. *Clinical Infectious Diseases* 2007; 44: 1520.

Gouws,E, Bryce J, Pariyo G, Armstrong Schellenberg J, Amaral J, Habicht JP. Measuring the quality of child health care at first-level facilities. Soc Sci Med. 2005 Aug;61(3):613-25.

Hart CA, Kariuki S. Antimicrobial resistance in developing countries. BMJ. 1998 Sep 5;317(7159):647-50.

Kahama-Maro J, D'Acremont V, Mtasiwa D, Genton B, Lengeler C. Low quality of routine microscopy for malaria at different health system levels in Dar es Salaam: Rapid Diagnostic Tests should also be implemented in hospitals and urban settings. Abstract in Am J Trop Med Hyg Suppl of the 57th Annual Meeting of the American Society of Tropical Medicine and Hygiene, New Orleans, USA, 7-11 December 2008.

Marx A, Pewsner D, Egger M, Nüesch R, Bucher HC, Genton B, Hatz C, Jüni P. Accuracy of rapid tests for malaria in travelers returning from endemic areas: systematic review and meta-analysis. *Annals of Internal Medicine* 2005; 142: 836-846.

Masanja H, de Savigny D, Smithson P et al. Child survival gains in Tanzania: analysis of data from demographic and health surveys. Lancet. 2008 Apr 12;371(9620):1276-83.

Mitchell, M, Mayhew, S, and Haivas, I. Integration Revisited, Millennium Project Background Paper on Integration of Services. Oct 2004.

Mitchell, M, Neal Lesh et.al.,Improving Care – Improving Access: The Use of Electronic Decision Support with AIDS patients in South Africa, International Journal of Healthcare Technology and Management (2008) in press

Mueller I, Betuela I, Ginny M, Reeder JC, Genton B. The sensitivity of the OptiMAL rapid diagnostic test to the presence of *Plasmodium falciparum* gametocytes compromises its ability to monitor treatment outcomes in an area of Papua New Guinea where malaria is endemic. *Journal of Clinical Microbiology* 2007; 45: 627-630.

Ochola LB, Vounatsou P, Smith T et al. The reliability of diagnostic techniques in the diagnosis and management of malaria in the absence of a gold standard. Lancet Infect Dis. 2006 Sep;6(9):582-8.

Okeke IN, Laxminarayan R, Bhutta ZA et al. Antimicrobial resistance in developing countries. Part I: recent trends and current status. Lancet Infect Dis. 2005a Aug;5(8):481-93.

Okeke IN, Klugman KP, Bhutta ZA et al. Antimicrobial resistance in developing countries. Part II: strategies for containment. Lancet Infect Dis. 2005b Sep;5(9):568-80.

Ray HN, Boxwala AA, Anantraman V, Ohno-Machado L. Providing context-sensitive decision-support based on WHO guidelines. Proc AMIA Symp. 2002, 637-41.

Schellenberg JA, Victora CG, Mushi A, de Savigny D, Schellenberg D, Mshinda H, Bryce J; Tanzania Integrated Management of Childhood Illness MCE Baseline Household Survey Study Group. Inequities among the very poor: health care for children in rural southern Tanzania. Lancet. 2003 Feb 15;361(9357):561-6.

Shirima K, Mukasa O, Schellenberg JA et al. The use of personal digital assistants for data entry at the point of collection in a large household survey in southern Tanzania. Emerg Themes Epidemiol. 2007 Jun 1;4:5.

Tanzania IMCI multi-country evaluation health facility survey study group, The effect of Integrated Management of Childhood Illness on observed quality of care of under-fives in rural Tanzania. Health Policy and Planning. 19(1): 1–10 2004.

Temu MM, Kaatano GM, Miyaye ND et al. Antimicrobial susceptibility of Shigella flexneri and S.

dysenteriae isolated from stool specimens of patients with bloody diarrhoea in Mwanza, Tanzania. Tanzan Health Res Bull. 2007 Sep;9(3):186-9

The Bellagio Study Group on Child Survival, http://cs.server2.textor.com/bellagio.doc

Trostle J. Inappropriate distribution of medicines by professionals in developing countries. Soc Sci Med. 1996 Apr;42(8):1117-20.

Victora CG, Huicho L, Amaral J, et al. Arehealth interventions implemented where they are most needed? District uptake of the Integrated Management of Childhood Illness strategy in Brazil, Peru and the United Republic of Tanzania. Bull World Health Organ 2006;84:792-801.

WHO. Interventions and strategies to improve the use of antimicrobials in developing countries. WHO/CDS/CSR/DRS/2001.9.

2.3 Rationale and relevance of the planned project

The rapidly changing patterns of disease and resistance patterns, the poor adherence to clinical guidelines using paper based algorithms and the increasing availability of new technologies for more accurate diagnostics and better adherence to evidence based clinical algorithms are the basis of this project. If we are to accurately diagnose and correctly treat acutely ill children, we must take advantage of these new technologies as they become available.

This project will test whether the technologies that are available today can be used to improve the quality of care that is being provided to Tanzanian children through the use of electronic decision support to promote evidence-based medicine and the use of rapid diagnostic tests which promote the rational use of drugs.

In particular, we expect to show that it is feasible and necessary to

- Adapt standardized diagnostic and treatment procedures for the management of childhood illness (IMCI) incorporating more sophisticated clinical algorithms based on current evidence about disease patterns and drug resistance;
- Incorporate guides for health workers to improve communication about diagnosis and treatment to mother or other caretaker;
- Rapidly incorporate these changes into treatment algorithms in an electronic format that is accurately used; and
- Demonstrate improved clinical and cost effectiveness of electronic algorithms when compared to the used of paper based IMCI algorithms leading to a reduction in the inappropriate use of antibiotics and antimalarials for children with fever.

2.4 Objectives

2.4.1 Goal

Improve the quality of health care available in developing countries by making *standardized diagnostic and treatments (or evidence-based medicine)* easily accessible to providers using electronic decision support systems, with a special emphasis on the rational use of antibiotics and antimalarials and improved understanding by mothers of diagnosis and treatment required at home following an IMCI clinic visit.

2.4.2 Project hypotheses

1. Use of decision support systems using mobile technology will enhance the ability to improve and rapidly implement evidence based clinical algorithms by improving adherence of health workers to these improved algorithms, leading to better health outcomes and more rational use of antimalarial and antibiotics, and better patient caretakers' adherence to clinician instructions when compared to paper-based decision charts or usual health facility procedures.

2. The use of mobile technology will reduce time and costs to implement improved IMCI algorithms compared to current practice of paper-based charts and training procedures.

2.4.3 Research questions

1. Can we improve IMCI protocols to be more specific about the use of antimalarial drugs and antibiotics for febrile illnesses?

2. Is the use of electronic algorithms more or less effective and efficient than papers based algorithms in implementing these changes in a field setting?

3. Is the use of new IMCI algorithms aimed at reducing overuse of antimalarials and antibiotics as safe as the old IMCI protocols (or routine practice)?

4. Can we improve IMCI protocols to improve clinician messaging communications so that caretakers better understand instructions for care of the child and subsequently adhere to prescribed advice such that patient outcomes improve?

2.4.4 Specific objectives

Objective 1: To develop IMCI guidelines including new diagnostic strategies such as RDT

Objective 2: To develop both traditional paper based and electronic algorithms and training materials for implementation of the new IMCI guidelines developed in Objective 1.

Objective 3: To compare the use of the old IMCI guidelines (or routine practice) and the new IMCI guidelines developed in Objective 1 in terms of the appropriate use of antimalarial and antibiotic prescription.

Objective 4: To compare the adherence of health workers to the new IMCI algorithms when using i) paper based decision charts and ii) electronic decision support algorithms.

Objective 5: To assess whether the new IMCI guidelines are equivalent in terms of health outcomes (safety) as the old IMCI guidelines (or routine practice).

Objective 6: To compare the economic costs of developing and implementing the new IMCI algorithms when using 1) paper based decision charts and 2: electronic decision support algorithms.

Objective 7: To compare health outcomes and mothers' understanding of the diagnosis and treatment requirements for a child after the IMCI visit comparing traditional paper-based IMCI care and e-IMCI with enhanced communications.

Objective 8: To compare the mothers' adherence to prescribed advice post-visit between the paper-based IMCI and e-IMCI with enhanced communications.

2.5 Methodology

2.5.1. Overview

The project will be conducted in 3 phases that will be conducted sequentially.

Phase 1: Preparatory phase (6 months) will include development of IMCI guidelines including RDT in the overall strategy as endorsed by NMCP and included in the Round 7 Global Fund proposal. The improved IMCI guidelines will be following current WHO amendments regarding fever management and integration of RDT. IMCI algorithms will be developed and both paper and electronic materials will be developed for use in training and implementation of these algorithms.

Phase 2: Study Phase (24 months) will be a randomized control study of the implementation, use and safety of new IMCI protocols for the assessment and treatment of children 2 months-5 years of age with fever, and for the communications between the health worker and the caretaker. This study will be conducted in randomly selected health facilities in urban Dar es Salaam and rural Kilombero / Ulanga districts and will have 3 arms:

a) training and use of new IMCI algorithms for fever using paper decision charts

b) training and use of new IMCI algorithms for fever using electronic algorithms

c) control: old IMCI or routine practice

A second component of this phase will be a study looking at the cost of developing and implementing new IMCI protocols using both the current paper based method and electronic algorithms.

Phase 3: Analysis and writing (6 months)

2.5.2. Specific methodology for each phase

2.5.2.1 Preparatory phase

The Preparatory phase will address the first two objectives:

Objective 1: To develop adapted IMCI guidelines including RDT in the overall strategy.

Objective 2: To develop both traditional paper based and electronic algorithms and training materials for implementation of the new IMCI guidelines developed in Objective 1.

Development of the updated decision tree (IMCI-derived)

For the last two years, we (VDA, JK &BG) have been testing and implementing an algorithm aimed at appropriately managing fever cases, both in highly endemic and moderately endemic areas of Tanzania. This decision tree was largely inspired from the IMCI one, but was adapted to accommodate a malaria test (usually RDT) and all ages in the assessment. We focussed the algorithm on identification of danger signs, as well as diagnostic and treatment procedures. We did not touch upon preventive measures and advice. For the present proposal, we intend to use the new WHO algorithm with possible amendments (under process) for children from 2 months to 5 years based on i) recent literature, ii) the evidence derived from the RDT malaria study. The appropriate choice of antibiotics will be discussed with national authorities to assess whether fist-line treatment for typhoid fever or pneumonia needs to be changed, following the findings in the recent literature and from our study on the etiology of fevers in urban and rural Tanzania (done over the last 3 years) and analysis of molecular markers of *Salmonella* resistance.

Develop clinician messages to caretakers:

Phase 1 will focus on testing the current practice of how health workers communicate with mothers about the child's illness and treatment. Based on this information, interventions will be developed that strengthen how these messages are conveyed to the mother. Examples of these interventions might include asking the mother to repeat back the care instructions, having the pharmacists repeat diagnosis and instructions at the time of dispensing medicines or the use of additional messaging to the mother once she has left the clinic. The results of phase I will be used to identify the most promising interventions to then test in Phase 2 of the study

Development of Chart booklet and Training materials

Once the protocols have been developed a paper based chart booklet and training materials will be needed for use by those clinicians who will implementing the new algorithms in the traditional way. The chart booklet will be designed to closely follow the current layout and color scheme (see Annex 3 for current chart booklet page for fever.) Tanzania has considerable expertise in the modification of chart booklets and these experts will be involved in the development process to ensure conformity with current practice. Once the chart booklets are modified to reflect the new fever algorithms, training curriculum will be developed for use in training clinicians who will be involved in the intervention arms of the study. Again, there is considerable expertise in this in Tanzania and in particular at IHI and the Dar es Salaam City Council City Medical Office of Health and we do not anticipate any problem with this aspect of the study.

Computerization of an electronic algorithm

Both Harvard School of Public Health and D-tree International have developed and tested an electronic version of IMCI in Tanzania and are currently undergoing a larger validation study (see under section 2.2.3). The electronic protocols are installed on either a mobile phone or PDA for use

by clinical officers and nurses in Tanzania. The software we have developed runs on a PDA and guides health workers step-by-step through the full IMCI assessment, classification and treatment plan. The current algorithms follow IMCI exactly and will need to be modified to reflect the changes to these algorithms that are recommended. We currently contract with programmers to do this type of programming and are working with IHI and other local partners to develop this capacity in Tanzania. Our experience is that this reprogramming is not difficult provided the algorithms themselves are clear. We have also found that once clinicians have been taught to use the electronic algorithms, they can easily adopt to new algorithms that are reprogrammed very quickly. Our experience to date with clinicians who have no previous computer experience but have used cell phones is that the training time to learn to navigate the electronic algorithms is less than 1 hour. For this study, the electronic algorithm will run on a mobile phone.

2.5.2.2 Study Phase

The Study Phase will be a randomized control study of the implementation and use of IMCI protocols for the assessment and treatment of children 2 months-5 years of age coming to the clinic. This study will be conducted in randomly selected health facilities in urban Dar es Salaam and rural Kilombero / Ulanga districts and will have 3 arms:

a) training and use of new IMCI algorithms for fever using paper decision charts

b) training and use of new IMCI algorithms for fever using electronic algorithms

c) control: old IMCI algorithm or routine practice (no intervention)

This design is intended to address the following 6 objectives:

Objective 3: To compare the use of the old IMCI guidelines and the new IMCI guidelines developed in Objective 1 in terms of the appropriate use of antimalarial and antibiotic prescription.

Objective 4: To compare the adherence of health workers to the new IMCI algorithms when using i) paper based decision charts and ii) electronic decision support algorithms.

Objective 5: To assess whether the new IMCI guidelines are equivalent in terms of health outcomes (safety) as the old IMCI guidelines (or usual procedures).

Objective 6: To compare the economic costs of developing and implementing the new IMCI algorithms when using 1) paper based decision charts and 2: electronic decision support algorithms.

Objective 7: To compare health outcomes and mothers' understanding of the diagnosis and treatment requirements for a child after the IMCI visit comparing traditional paper-based IMCI care and eIMCI with enhanced communications.

Objective 8: To compare the mothers' adherence to prescribed advice post-visit between the paper-based IMCI and eIMCI with enhanced communications.

This study will be done as a prospective randomized controlled study in health facilities (government run health centres and dispensaries) that are randomly selected from the total number of sites in Dar es Salaam and Kilombero/Ulanga stratified by location (Dar es Salaam and Kilombero/Ulanga and type of facility (health centre and dispensary). Our initial estimate is to use 24 sites with 8 in each arm of the study.

Training component

A formal training on the new IMCI algorithm will be done for all clinicians taking care of patients in the health facilities selected for interventions. In those under a) only paper based decision chart will be used,; in those under b) paper based decision chart will be used first for visualizing and understanding the new algorithm, and then the latter will be removed and replaced by PDAs with formal training on their use. No training, outside advice provided on site when requested, will be undertaken for clinicians under c). The same type of procedures has been used in the previous project (IMALDIA) (D'Acremont 2008a).

Study to assess appropriate use of drugs, estimation of provider's adherence and mother's understanding of prescribed advice (objective 3, 4 & 7):

The total sample size will be about 960 patients in Dar es Salaam and 960 in Kilombero/Ulanga (i.e. 1920 patient consultations observed). The randomization will be by site (health facility) rather than by individual patient so that all patients at an individual site will be assessed using either the original IMCI guidelines, the new IMCI paper guidelines or the new IMCI electronic guidelines. The first half of all patients will be done 4 weeks after training and the second half one year after training to investigate the sustainability of the intervention effect overtime.

Subjects: All voluntary patients between 2 months and 59 months coming to an outpatient facility in the study with an acute illness.

Procedures: Each child between 2 and 59 months brought to a clinic for acute care will be seen by the clinician using the protocols implemented in that particular health facility [original IMCI (or usual practice) or new IMCI paper chart or electronic IMCI protocol] as they would normally. Patient's caretakers will be asked whether they are willing to participate in this study. Those who agree will be read an informed consent script and asked to sign their consent.

If a patient's caretaker agrees, a trained observer will be present to records details of the clinician's consultation and assess whether all procedures mentioned in the IMCI protocol are adhered to or not. A standardized form will be developed to record these observations as well as the time for each encounter. In addition, following the usual consultation, the caretaker will be interviewed to assess comprehension of instructions regarding treatment and follow-up and a trained IMCI expert will repeat the patient assessment to compare the results of his/her formal IMCI assessment with that of the usual clinician. The expert will so determine the IMCI "gold standard" classification and treatment. If the expert feels that the safety of the child is compromised (e.g. diagnosis of pneumonia but no antibiotics prescribed), the expert will adapt the prescription (e.g. add anti-infective drugs). We have developed a set of indicators to assess whether a child is correctly assessed, classified, treated and counselled for use in the IMCI adherence study currently underway. These indicators are based on a very large scale multi-country supported by WHO in 2000 (Gouws, 2005) to assess the quality of care using IMCI compared with non-IMCI. For both observation and re examination phases of the study, the data collection tools will be adapted from the IMCI Multi Country Evaluation instruments (http://www.who.int/imci-mce/Publications/HFS_instruments.pdf).

Outcome measures: adherence to procedures in IMCI protocols, accuracy of diagnosis category, appropriateness of antimalarial and antibiotic prescription, , caretakers' understanding of advice.

Analytical plan: outcomes will be compared between the two intervention groups (new e-IMCI & new paper IMCI) against the control one (C).

Study to assess safety and adherence of caretaker to prescribed treatment and advice (objective 5 & 8):

The total sample size will be about 1200 patients in Dar es Salaam and 960 in Kilombero/Ulanga in the same randomised health facilities (total of 2160). The first half of all patients will be done 4 weeks after training and the second half one year after training to investigate the sustainability of the intervention effect overtime. The patients will be different than those included in the assessment of appropriate use of drugs and adherence, since the latter might be in more 'controlled' conditions than those included in this component, and less likely to have an unfavourable outcome.

Subjects: All voluntary patients between 2 months and 59 months coming to an outpatient facility in the study with a history of acute 'febrile' illness.

Procedures: Each child between 2 and 59 months brought to a clinic for acute care will be seen using the protocols implemented in that particular health facility (original IMCI or new paper chart or electronic IMCI protocol) as they would normally. The patient's caretakers will be asked whether they are willing to participate in a follow-up study. Those who agree will be read an informed consent script and asked to sign their consent. If a patient's caretaker agrees, a trained observer will be present to records details of the clinician's consultation and assess whether all procedures mentioned in the

IMCI protocol are adhered to or not. The diagnosis classification and treatment given will be recorded, as well as a cell phone number if available. The patient's caretaker will be asked to come back after 7 days, so that the child can be assessed for health outcome [cured, not cured (if not cured: worse, stable better)]. If not cured at day 7, they will be reassessed at day 14. A standardized form will be developed to record these observations. As is true in IMCI protocols, children's caretakers will be advised to come back to health facility in case the child is not better or gets worse after 1 or 2 days depending on severity of illness. They will be seen by dedicated staff and assessed clinically in case of unscheduled reattendance or readmission. Additionally we will use this opportunity to test the mothers' understanding of the diagnosis and treatment of the child and whether the prescribed treatment was in fact followed correctly.

Outcome measures: The primary outcome will be the rate of unscheduled reattendance and percent of caretakers who follow prescribed advice. Secondary outcomes will be the rate of patients cured at day 7, the rate of delayed complications and the rate of drug prescribed.

Analytical plan: outcomes will be compared between the two intervention groups (e-IMCI & paper IMCI) and the control one (C).

Similar design and procedures were used previously to assess the safety of not treating febrile children with a negative rapid test for malaria and proved to be quite feasible. The percentage of children followed-up was excellent (97%); the rate of unscheduled reattendance was 12% (D'Acremont 2008b)

Study to estimate cost

A cost study of both paper based and electronic IMCI will be conducted to address the following objective:

Objective 6: To compare the economic costs of developing and implementing the new IMCI algorithms when using i) paper based decision charts and ii) electronic decision support algorithms.

This study will collect cost information and develop a model to ascertain the cost per child seen using both e-IMCI and traditional IMCI and then assess the relative cost-effectiveness on a cost per child correctly treated measure of outcome. This is the same measure that was used in the multi country IMCI evaluation done by WHO in Tanzania (Armstrong, 2004) and so provides an excellent basis for comparison. (Tool available at http://www.who.int/imci-mce/Publications/facility_costs.pdf)In addition to the cost of seeing a child with an acute febrile illness, we will also collect data on the cost of changing the paper based chart booklet and training the health worker in its use. This will be compared with similar data collected to calculate the costs of reprogramming and training a health worker to use the electronic algorithm.

The study will look at the cost of implementation of both paper and electronic IMCI on the basis of time spent by a clinician and the cost of equipment or supplies. For capital goods such as the PDA or booklet, it will be assumed that the useful life of each is approximately 2 years.

Sample size calculation

Appropriateness of drug use, provider's adherence and mothers' understanding of prescribed advice (objectives 3, 4 and 7): The feasibility and adherence study will include 1920 patients (640 per arm). For objectives 3 and 4, this sample size is adequate to detect a significant difference between each arm on the rate of antibiotic prescription assuming that among malaria negative patients (80% of all), 60% will be prescribed antibiotics in the control arm versus 30% in those using the new protocol (intervention groups). For objective 7, this provides 80% power to detect a 20% difference in caretaker comprehension immediately after the clinic visit, at a 0.05 significance level This large sample size is required taking into account the need for stratification by time period and setting (urban vs rural), as well as the potential heterogeneity due to the randomisation per health facility.

Assessment of safety study and caretaker adherence to prescribed treatment and advice (objective 5 & 8): The safety study will include another 2160 patients (720 per arm). This sample size is sufficient to demonstrate equivalence (non-inferiority) of the new algorithm when compared to old IMCI (or routine practice) on the primary outcome assuming a probability of 0.12 of unscheduled reattendance in all arms and an acceptable difference <0.05 (power 80%), with 10% loss of follow-up

(http://www.ucalgary.ca/~patten/blackwelder.html). An equivalence study has been chosen since we aim at achieving at least similar health outcomes but with less drug prescribed (antimalarials and antibiotics) with the new IMCI algorithm. The sample is also sufficient for 80% power to detect a 20% difference in caretaker actions one week after the clinic visit, at a 0.05 significance level.

2.5.2.3 *Phase 3: Analysis and writing*

The analysis will focus on answering the three research questions.

1. Can we improve IMCI protocols to be more specific about the use of antimalarial drugs and antibiotics for febrile illness?

This will be analyzed by comparing the results of arm a) and b) against control arm c) to determine whether the new protocols do improve the overuse of anti-malarials and antibiotics (primary outcome: rate of children being prescribed antibiotics).

2. Is the use of electronic algorithms more or less effective and efficient than papers based algorithms in implementing these changes in a field setting?

This will be analyzed by comparing the results of arms a) with b) to determine the adherence of health workers to the protocols using paper based or electronic algorithms, as well as costs (primary outcome: appropriate diagnosis classification and use of antimalarials and antibiotics).

3. Is the use of new IMCI algorithms aimed at reducing overuse of antimalarials and antibiotics as safe as old IMCI protocols(or routine practice)?

This will be analyzed by comparing the results of arms a) and b) with control arm c) to determine whether the new protocols leads to similar health outcomes (equivalence study) (primary outcome: unscheduled reattendances).

4. Can we improve IMCI protocols to improve clinician messaging communications so that caretakers better understand instructions for care of the child and subsequently adhere to prescribed advice to caretakers such that patient outcomes improve?

The analysis will be done be by comparing the results of arm b) with the paper arm a) and control arm c) to determine whether the electronically enhanced communications between health worker and caregiver leads to an improved understanding by the caregiver of what medicines to give the child and whether this leads to improved health outcomes.

2.5.2.5 Ethical considerations

The project has been already discussed in depth with the different partners, including the ethical considerations. When a project is aiming at improving adherence to universally accepted recommendations such as IMCI guidelines, there is no definite arm to be expected. On the reverse, there is a hope that patients, and especially children will benefit from the intervention planned. The usual criticism of PDA-assisted diagnosis and treatment is that clinicians may feel less concerned and may not investigate the peculiarities of the patients they have to care. However, we have seen during our previous project that the level of care is very poor and it is unlikely that such a tool could lead to worse care. What has been observed in the past with such tools is that the clinicians tend to acquire an automatism that is beneficial for the patient, as long as the automatism complies with updated guidelines, which will be the case for yours. These tools are also very useful as a teaching and training material. If the expert feels that the safety of the child is compromised (e.g. diagnosis of pneumonia but no antibiotics prescribed), the expert will adapt the prescription (e.g. add anti-infective drugs).

The choice to include a control arm (in this instance usual procedures of the health facility) should always be questioned, especially so when the quality of care is known to be below standard. However, the control group is critical in our study to ensure safety of the new IMCI-derived algorithm. Without control arm, we may miss a deleterious effect of the new algorithm on health

outcomes. It is also important to document the impact on the number of drugs prescribed: reducing drug intake is indeed an essential aspect of safety. Lastly, the inclusion of a control group allows a refined assessment of the effectiveness of i) training and supervision of the use of a new algorithm, ii) same but based on an electronic support rather than paper.

Eligible patients and their caretaker will be read an information sheet so that they can give their informed consent if they agree to participate in the study.. The project will be conducted in health facilities selected by the DSM City Council City Medical Office of Health or the District Medical Office in Kilombero/Ulanga and where the person in charge and the staff agree.

2.6 List of individual tasks and responsibilities of the partners (see also section 3.4)

Results	Activities foreseen to reach the results	Indicators with milestones (in months from project start)	Responsible project partner
R1: Computerization of new IMCI e-algorithm and training material			
	A 1.3: Design of new IMCI algorithm	I 1.3.1: New IMCI algorithm (6 months)	STI (collaboration with IHI and CMOH)
	A.1.4 Development of paper based decision chart and training material	I 1.4: Paper decision charts and training brochures (6 months)	IHI & CMOH (collaboration with STI)
	A.1.5: Computerization of new IMCI algorithm	I 1.5: Computerized new IMCI algorithm (6 months)	D-Tree (collaboration with IHI and STI)
	A1.6: Addition of messages from HW to mother in eIMCI algorithm	I 1.6: New eIMCI algorithm with improved messaging incorporated (6 months)	Harvard School of Public Health (collaboration with IHI and STI)
R2: Training of clinicians for new IMCI paper and e-IMCI protocols in intervention arms	A.2.1: Training of clinicians	I 2.1: Clinicians from intervention arms trained for new IMCI paper protocols and e-IMCI (12 months)	CMOH (Dar es Salaam), IHI (K/U districts)
R3: Adherence and appropriate use of drugs	A.3.2: Observation and assessment of 1920 patients consultations	I 3.2: Diagnostic classification, appropriate prescription of antimalarials and antibiotics (30 months)	CMOH (Dar es Salaam), IHI (K/U districts)
R4: Safety of new IMCI algorithm	A.4.1: Follow-up of 2160 patients	I 4.1: Unscheduled reattendance (24 months)	CMOH (Dar es Salaam), IHI (K/U)
R5: Costs	A.5.1: Costing of 300 children	I.5.1: Cost (27 months)	CMOH (Dar es Salaam), IHI (K/U districts)
R6: Conference abstracts and papers	A.6.1: Analysis and writing	I.6.1: Accepted abstracts and papers	STI, IHI, CMOH

Swiss Tropical Institute:

Tasks: Development of the updated decision tree (IMCI-derived); training and field activity support; support for data analysis and writing;
Responsibilities: Overall project coordination; knowledge transfer; academic support; accountability to the funding agency.

Ifakara Health Institute:

Tasks: Development of chart booklet and training material (with STI and CMOH); training of health staff (with STI and CMOH); implementation of PDAs and chart booklet assisted diagnosis and treatment in selected health facilities of K/U; observation and evaluation of consultation processes;

data entry and cleaning; data analysis and writing with support of STI.
Responsibilities: Overall project coordination in the field; conduct of the project on the ground in K/U districts with support of STI; accountability to the STI for expenses in Tanzania.

Dar es Salaam City Council City Medical Office of Health

Tasks: Development of chart booklet and training material (with STI and IHI); training of health staff (with STI); implementation of PDAs and chart booklet assisted diagnosis and treatment in selected health facilities of Dar es Salaam; observation and evaluation of consultation processes; data analysis and writing with support of STI.
Responsibilities: Conduct of the project on the ground in Dar es Salaam with support of IHI and STI.

Harvard School of Public Health

Tasks: Developing the additional messaging and programming them into the improved electronic algorithm; provide mobile phones with new eIMCI programmed.

Responsibilities: Additional programming and design of NIH-specific patient follow-up visits and clinician messaging.

D-tree

Tasks: Computerization of the electronic algorithm; PDA training and maintenance.
Responsibilities: Electronic component of the project.

3 Development relevance of the planned research

This project addresses two key factors critical for the improvement of quality of health care in developing countries: (1) the need to continually revise and update evidence based clinical medicine as new evidence and technologies become available, and (2) the need to increase use of these revised standards in a timely and cost effective way. It builds on a significant body of working showing that clinical algorithms such as IMCI can be used to improve health outcomes if used correctly but that current practice is often not sufficiently adhered to the guidelines to yield the desired results.

First in this work, we will show that the introduction of improved treatment guidelines in Tanzania, and indeed in all of Africa, is both feasible and cost effective. Given the rapidly changing disease and drug resistance patterns that are emerging worldwide, it is critical that changes to standardized treatment guidelines can be done rapidly and effectively even in resource poor environments.

The thorough assessment of PDA-assisted decision making for the management of childhood illness should lead, if successful, to a large improvement in guidelines adherence since there is less room for error or alternation of the algorithms based on individual preference rather than substantiated evidence. The assessment of the safety and cost-effectiveness of PDA electronic algorithms versus paper decision charts or usual procedure will help health care providers, policy makers and national health authorities to choose on the best strategy to ensure that good quality of care is given to the most at need.

3.1 Significance of the planned research for the economic and/or societal development of the partner country

Cutting edge technologies should not be restricted for use in developed countries only. On the contrary, they offer the opportunity to circumvent numerous constraints in terms of human resources and specific competence that are often lacking in developing countries, especially in Africa. Technologies can be used to improve diagnosis and care and to allow task-shifting from medical doctors to clinical officers, nurses, and community health workers if they are given the support they need to maintain high quality care. PDA-assisted decision support for best practice at primary health facilities should improve the quality of care provided, and therefore contribute towards lowering child mortality, both in urban and rural areas. At the same time, the huge burden on health facility budget posed by the overprescription of antimalarial and antibiotic drugs should be drastically reduced, and resources reallocated to more effective preventive or curative strategies. The investigation of the level

of bacterial resistance to common drugs should help policy makers to decide on the most appropriate antimicrobial to use for treating prevalent infectious diseases. Lastly, the potential for PDAs to drastically improve health information systems (routine health statistics, amount of drug prescribed and sold etc.) can lead to considerable economical benefit and a more rational use of limited resources.

3.2 Planned actions to strengthen partner country researcher and research institution capacities

Research capability in the clinical field is currently limited in Tanzania. The present work will strengthen this in a number of ways:

1) One Tanzanian clinician will be given the opportunity to carry out a substantial body of research work and get a PhD degree. This person will be affiliated to the IHI and get her/his degree from the University of Basel. He will spend 3 months per year in Switzerland for attending courses (epidemiology and statistics mainly) and the rest in Tanzania to do the field work. She/he will form a partnership team with a clinician from a developed country (probably Switzerland), so that they can benefit from mutual teaching/learning.

An additional research coordinator will be hired to work on this study. This coordinator will be affiliated with Harvard University and will primarily be responsible for the management of the study component that relates to improved communications between the health worker and the mother of the ill child. In addition, a Tanzanian field implementer will be hired to assist with the research.

2) A number of individuals within the Dar es Salaam City Council and the Ifakara Health Institute will be associated with this work and acquire clinical research experience.

3) Health staff performing usual medical duty in the health facilities will be involved in this work, and hence exposed to research aspects that should improve their skills relevant for their day-to-day practice. They should also acquire expertise in novel technologies that are yet restricted to privileged medical centres.

4) As the project evolves, it is foreseen to raise additional resources for ancillary studies and integrate more junior Tanzanian clinicians and researchers, for example in the frame of a MD or MSc thesis in medical informatics, health information systems and/or medical anthropology.

3.3 Strategy to communicate with intended results users

The first Tanzanian partner is the City Medical Officer of Health for Dar es Salaam which is the executive body for the planning and management of all health facilities in Dar es Salaam. Dr JK is at the same time researcher and City Medical Officer, and is therefore directly in contact with national authorities, policy makers and grassroots medical personal. She is the direct link between the researchers, Ministry of Health and result users, i.e. the medical staff caring for sick children and adults in the health facilities of Tanzania.

The second Tanzanian partner is the Ifakara Health Institute which is heavily involved in developing and assessing new tools aimed at improving child health in rural districts. The IHI, and its director in particular, is also involved in decision-making at the country level. He is very concerned to translate research findings, if effective, into policy, and to assess their impact when implemented under programmatic conditions. Recently, a resources centre has been established within IHI with the aim of disseminating research findings to the media, and hence public, as well as to all interested institutions in the country and elsewhere.

As an illustration, we describe below the process followed during the previous project (IMALDIA) with the same partners. Following the successful implementation of RDTs in health facilities and the demonstration of the safety of the strategy based on laboratory diagnosis rather then presumptive diagnosis, and the impact on antimalarial prescription, we were involved at the national level in the discussion for policy change. After scrutinizing our preliminary results, and even before project completion, the National Malaria Control programme, together with us as researchers, wrote new

guidelines for the management of fever patients. A proposal to the Global Fund for AIDS Tuberculosis and malaria was then submitted in Round 7, including a substantial component for RDTs implementation country-wide. This proposal was recommended for funding. This shows that a stepwise process, including first demonstration of the reliability of the new technology, then proof of its feasibility and safety in close-to-programmatic conditions, and finally demonstration of its expected impact, is likely to attract long-term funding and ensures thus sustainability of the intervention.

We intend to follow the same process in the present project, should the intervention proposed (PDA decision support system) prove to be feasible and cost-effective.

3.4 Distribution of duties, competencies and responsibilities between the partners

We have 5 main partners, one from Switzerland, 2 from Tanzania, one from USA, and one from both USA and Tanzania. The relevant duties, competencies and responsibility are as follows:

Swiss Tropical Institute:

Duties: Design of revised IMCI protocol; selection of counterpart clinician from developed country; support for activities in the field as well as for data analysis and writing.
Competencies: Knowledge on i) evidence based medicine, ii) practice guidelines design; iii) research procedures in developing countries.
Responsibilities: overall project coordination; knowledge transfer; academic support; accountability to the funding agency

Ifakara health institute:

Duties: Implementation of the project on the ground in conjunction with health authorities of Kilombero/Ulanga districts, more specifically for i) selection of research staff (clinicians-observers; field coordinators etc.); ii) selection of health facilities, iii) contact with person in charge in health facilities, v) training of health staff on new protocol, vi) conducted of planned studies; data entry; data cleaning and analysis; writing.
Competencies: Knowledge on research procedures, especially in Tanzania; contact with IRB and NIMR ethics committee; contact with academic institutions in Tanzania.
Responsibilities: Overall project coordination in the field; conduct of the project on the ground in K/U districts with support of IHI, HSPH and STI; accountability to the STI and HSPH for expenses in Tanzania.

Dar es Salaam City Council City Medical Office of Health

Duties: Implementation of the project on the ground in Dar es Salaam together with IHI, more specifically for i) selection of counterpart clinician from Tanzania (PhD candidate), ii) selection of research staff (clinicians-observers; field coordinators etc.); iii) selection of health facilities, iv) contact with person in charge in health facilities, v) training of health staff on new protocol, vi) conducted of planned studies in selected health facilities in Dar es Salaam; data analysis and writing.
Competencies: Knowledge on i) health care management and organization, ii) clinical, diagnostic and treatment procedures; contact with relevant partners within the health system and with the Ministry of Health;
Responsibilities: Conduct of the project on the ground in Dar es Salaam with support of IHI and STI.

Harvard School of Public Health

Duties: Design of revised electronic IMCI protocol that incorporates additional messaging; selection of research coordinator and field assistant for field research in Tanzania; support for activities in the field as well as for data analysis and writing.

Competencies: Extensive experience in the design of research studies, research procedures in developing countries and evidence-based medicine.

Responsibilities: Overall project coordination for Harvard-specific study components; knowledge transfer; academic support; accountability to the funding agency

D-tree

Duties: Computerization of the electronic algorithm; PDA training and maintenance.
Competencies: Knowledge on i) IMCI strategy, ii) electronic protocol design and implementation.
Responsibilities: Electronic component of the project.

3.5 Handling of gender balance and 'do not harm'

The target group for the intervention in this project are all children aged 2 months to 5 years. There will be no therefore no distinction between girls and boys in terms of health benefit.

As far as the project management and coordination is concerned, two out of the 5 investigators are women (VDA & JK). They have been the main players of the previous joint project (IMALDIA) which employed more women than men altogether. These women will be sensitive to the gender issue, as are all other investigators on this project, as can be demonstrated by their recent publication list that include as many, if not more women than men.

The 'do not harm' principle forms the basis for this project which aims at improving the quality of health care for Tanzanian children. Promoting appropriate diagnostic and treatment procedures and rational use of drugs is likely to lead to health benefit rather than harm. The safety assessment of the new algorithm addresses the 'do not harm principle' since it aims at demonstrating equivalence in terms of health outcomes with less drugs prescribed.

4 Management structure

The project will be under the overall responsibility and coordination of the STI, through its two investigators (BG & VDA) who will act as guarantors. They will ensure that the project is conducted under the laws and regulations of both Switzerland and Tanzania and under the principle of good clinical practice. They will be responsible for the provision of scientific and financial reports to SNF in due time.

The preparatory phase will be under the direct responsibility of STI (laboratory component), together with the clinician from the North and the clinicians at IHI and CMOH (development of decision charts and training material). STI will mandate D-Tree for the fine tuning of the algorithm (MM) and the computerization of the algorithm (NL). In addition, MM and NL will develop the communication messages to be used in later stages of the project and oversee that component of the study.

All activities in Tanzania will be under the direct responsibility of the Tanzanian main applicants (HM & JK), with support and technical support from the Swiss main applicants and other collaborators. The CMOH will be responsible for activities run in Dar es Salaam and IHI for those run in K/U.

The clinician from the North and the Tanzanian clinician (PhD student) employed by the project will be involved in the preparatory phase (all levels but laboratory) and be responsible for the day-to-day running of the study phase and for the main analysis and writing. The will both based in Tanzania. It is common practice that PhD students from Tanzania spend approximately two months per year in Switzerland to attend courses.

In terms of location, the preparatory phase will be done both in Switzerland and Tanzania, while the study phase will be run exclusively in Tanzania.

In case of major problems arising in Tanzania, STI investigators will convey a meeting on site with the other investigators and partners to solve the issue.

The health care of patients will remain the responsibility of the clinicians in charge at the health facility that will work independently of the project.

It is foreseen to have at least two annual project meetings with all senior staff involved, one in Tanzania and one in Basel. These procedures are standard for a long time already in the interaction between Dar es Salaam CMOH, IHI and STI.

5 Institutional ethical clearance

The protocol, annexes, study information and any other necessary documents will be submitted to the Ethikkommission beider Basel (EKBB), to the Institutional Review Board of the Ifakara Health Institute, Harvard School of Public Health Institutional Review and the National Institute for Medical Research Review Board for approval.

6 Time Table

The timing of the most important activities of the project are shown below

Time table												
	First year Mar 10-Feb 11				Second year Mar 11-Feb 12				Third year Mar 12-Dec 12			
**Quarters**	1st	2nd	3rd	4th	1st	2nd	3rd	4th	1st	2nd	3rd	4th
Planning, staff recruitment												
**Preparatory phase**												
Development of updated algorithm												
Development of training material												
Computerization of e-algorithm												
**Study phase**												
Identification of health facilities												
Training												
Safety assessment												
Adherence assessment												
Cost study												
**Analysis and writing phase**												
Analysis and writing up of results												

7 Account of "Guidelines for research partnership with developing countries – 11 Principles"

1 Decide on the objective together

The present work is the logical continuation of a work undertaken in 2006 with the same partners on improving the diagnosis of malaria in Dar es Salaam and Kilombero/Ulanga district (IMALDIA). The main finding from this work was that a proper diagnosis of malaria could be made thanks to the use of RDT for malaria, but the clinicians lacked basic knowledge to identify another cause of fever, and hence to manage these cases appropriately. They therefore applied a blanket treatment of antibiotics to almost all malaria negative patients, leading to a dramatic overprescription of these drugs and a huge potential for the rapid spread of bacterial resistance. These findings led the CMOH and IHI to request STI to carry out the next step to ensure a more rational use of drugs, and hence better quality of care delivery. Since this fitted well with the research interests of the Swiss partners present on site, the present proposal could be prepared in a fully collaborative manner in Dar es Salaam.

2 Build up mutual trust

The Dar es Salaam City Medical Office of Health - STI collaboration goes back to 1989 and has been constantly developed since. The now IHI, ex- Ifakara Health Research and Development Centre, - STI relationship goes even back further to 1957. Hence, there is a high level of trust based on numerous previous achievements. One Tanzanian partner got his PhD degree at the STI in 2002 and the other one is supposed to get in 2009.

3 Share information, develop networks

The strong relationship between the Dar es Salaam CMOH, the IHI and the STI is also based on a large shared international network of collaborators, and a constant flow of information between Basel, Dar es Salaam and Ifakara. This is also shown by the numerous other common scientific projects by the investigators (in the area of infectious diseases).

4 Share responsibility

Most (if not all) implementation responsibility for the proposed work lies in the hand of the CMOH and IHI staff, and this is natural since most of the work will be carried out on-site. We also maintain full scientific and administrative transparency so that all senior staff is fully aware of all aspects of the work and administration.

5 Create transparency

As stated above, we have a full scientific and administrative transparency. The budget, as outlined in the present proposal provides the basis for resource allocation and this has been discussed and approved by all involved.

6 Monitor and evaluate the collaboration

Given the long-standing relationship between CMOH, IHI and STI and given the fact that project meetings are planned twice-yearly with the aim to systematically review all aspects of work, including our institutional and personal relations, it is not foreseen to have a separate formal evaluation of the partnership component.

7 Disseminate the results (see also section 3.3)

The CMOH-IHI-STI long-term partnership has always been based on a fair share of the results from studies, with no limitation of access. Presentations in international scientific meetings and publication in scientific journals by all team members has been actively encouraged, and the outstanding publication record of the collaboration is a testimony that this has happened.

8 Apply the results (see also section 3.3)

Our project is highly applied, with an immediate benefit to the health services and the target population. Through the tight systemic integrations and also through the different official function of the Tanzanian investigators the rapid implementation of the research findings can be ensured. We are also planning dissemination at national and international level, to ensure that the results are feeding as soon as possible into further operations.

9 Share profits equitably

Our planned work has no commercial prospects.

With regards to publication, citation as author will be based uniquely on the inputs made to the project, and neither on institutional affiliation nor official function. A fair and transparent system is being used for the last 10 years and we plan to use it for our project.

10 Increase research capacity

See section 3.3

11 Build on the achievements

The long-standing relationship between the Dar es Salaam CMOH, IHI and STI is testimony that we strive to build permanently on the results achieved through our partnership to strengthen the institutions on one hand, and promote further high-quality research on the other hand. Part of this is to train and retain high-quality staff. Also, the present proposal directly addresses issues that have arisen from the achievements made by the successful implementation of the proposal aimed at improving the diagnosis of malaria using RDTs. D-tree International has been working for the past year with the IHI on the development of electronic IMCI algorithms.

Annex 1: Study areas

Dar es Salaam

Dar es Salaam is the economic city of the United Republic of Tanzania, on the East coast of Africa. The current surface area is 1'393 sq. km and the population was estimated to be about 2'500'000 in 2002. As many other sub-Saharan African cities, Dar es Salaam has grown rapidly over the last decades. Dar es Salaam is governed by a political structure consisting of three levels: region, district and division. The three districts (Kinondoni, Temeke and Ilala) are headed by commissioners. The health sector is organised under the City Medical Office of Health (CMOH). Each of the three urban District Medical Offices (DMO) is responsible for and manages one municipal district hospital, one or two health centres and 13-14 dispensaries. In addition, there are non-government health facilities and a thriving private practitioners sector.

From 1990 until 2002 the Swiss Tropical Institute (STI) was the executive agency of the Dar es Salaam Urban Health Project (DUHP), which rehabilitated existing infrastructure and improved management practices (Wyss et al., 2000). From 2000, the IHI was heavily involved in assessing different malaria control strategies appropriate for the specificities of malaria epidemiology in urban setting. From 2005, the project of implementation and impact evaluation of RDTs has been conducted and is still ongoing under a partnership between STI, CMOH and IHI, the Chief Medical Officer (Dr D Mtasiwa) being the principal investigator in Tanzania. As a result, there is a longstanding and strong partnership between the STI – CMOH - IHI, which allows fruitful operational research to be conducted.

Kilombero /Ulanga districts

The Ifakara branch of the IHI is located in southwest Tanzania (350 Km from Dar es Salaam), and is the oldest one. The research station is home a variety of research ranging from entomology of the mosquito to HIV clinical studies. An important resource that was initiated by Ifakara in 1996 (and is still ongoing) is the Demographic Surveillance System (DSS) study which involves regular surveys of registered residents recording all births, deaths, and in and out migrations in a geographically defined area of 25 villages of the Kilombero and Ulanga districts. The laboratory unit at Ifakara is composed of molecular biology, microbiology and immunology divisions. It is connected to the internet by use of VSAT through the Mimcom network.

The IHI, situated in the premises of the St Francis designated district hospital (SDDH), has a long-standing collaboration with all health facilities located in the DSS area, especially for the morbidity monitoring activities. Numerous malaria projects have been conducted in these health facilities, in particular in vivo studies to assess the efficacy of different antimalarial drugs and molecular studies to look at the genetic profile of circulating malaria parasites. More recently, a large project aiming at assessing and improving quality of care in these same health facilities has been initiated (ACCESS project). Part of it is the implementation and evaluation of clinical algorithms for childhood illness including laboratory diagnosis of malaria through RDTs. Because the staff of these health facilities is now familiar with RDTs and its implication in terms of management of fever, we intend to conduct our new proposal in the same health facilities, as a continuation of the search for better quality of care.
