# Supplementary material for: New Algorithm for Managing Childhood Illness Using Mobile Technology (ALMANACH): A Controlled Non-Inferiority Study on Clinical Outcome and Antibiotic Use in Tanzania
Source: PLoS One. 2015 Jul 10;10(7):e0132316. doi: 10.1371/journal.pone.0132316 (PMC4498627; doi:10.1371/journal.pone.0132316)
Supplement: S5 File — (PDF) [file pone.0132316.s005.pdf]

ALMANACH APPLICATION USER MANUAL

The device: Samsung Galaxy tab 7.0 plus

Turning on and off

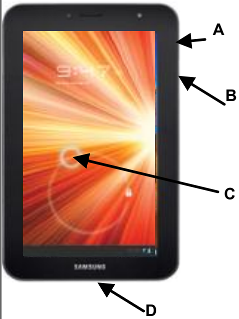

Turning On:
Press and hold the Power/Lock Key (A)

Turning Off:
Press and hold the Power/Lock Key (A)
At the prompt, touch Power off.

Unlocking the Device
Press the Power/Lock Key (A).
Touch and drag the unlock icon to the edge of the circle that appears, as shown. (C)

Charging: Plug charger in the charger port (D)

B: Volume key

On the home page

Applications shortcuts: Touch to open

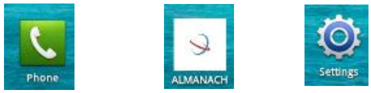

Phone dialer | ALMANACH | Settings

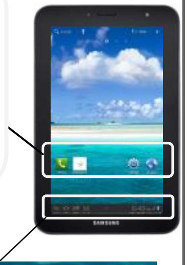

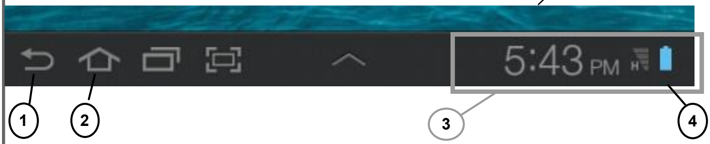

1. Back arrow: to return to the previous screen or option.
2. Home: to go back/display the central Home screen.
3. System Bar: displays navigation buttons and icons that show notifications, battery power, and connection details.
4. Status Icons
Touch the time/status icons area to display a quick settings menu.

The application: ALMANACH

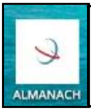

Launch the application

Touch the ALMANACH icon from the home page
Or from the applications' menu Apps

Login

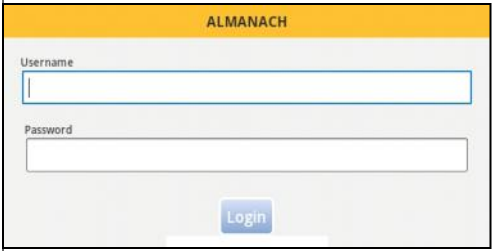

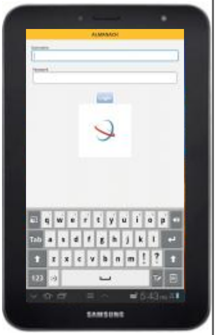

Type in user name and password
then touch « login »

Keyboard: Appears when one touches a space where typing is required e.g. Username, password, weight, temperature, etc

Main screen menu

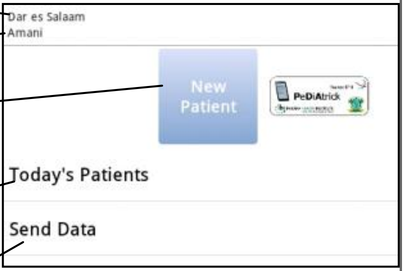

Location

Code of the user

New patient: Touch here to begin a new case in ALMANACH.

Today's patient: Touch here to see and/or retrieve cases saved today.

Send data: Touch here to send all data to the server. The data sent will be saved and be used to generate reports for the health facilities

Navigating

How to navigate:
When a new case is opened, swipe your finger in a horizontal motion from right to left to navigate forward, or left to right to go back.

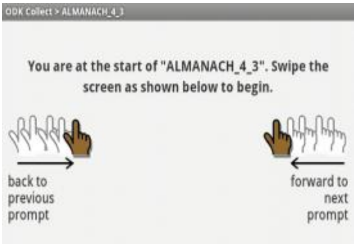

Follow the commands/instructions:
In each screen you will find instructions and commands that will help you to navigate through the algorithm and to decide the best treatment option for the child.

Example: Registration screen

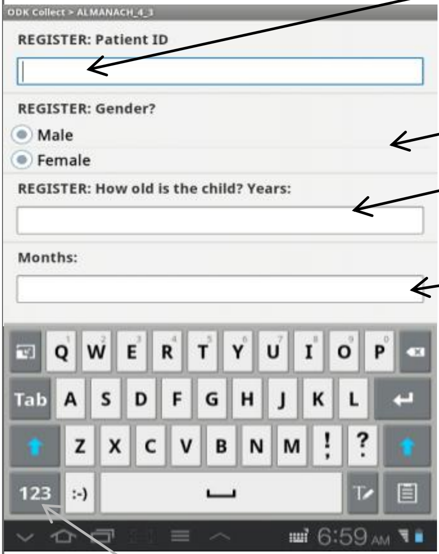

Touch here to type patient's registration ID. This code will be used to retrieve incomplete form in ''Today's patients'' list.

Select patient's gender

Type patient's number of years. Type 0 if age is less than 12 months

Type patient's number of months.

Touch the 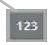 button to display numerical keyboard to enter numbers.

Change language option

ALMANACH application exists in English and in Kiswahili. You can change the language from one to the other when a form is loaded by following this procedure:

From any screen of the form, touch the menu button

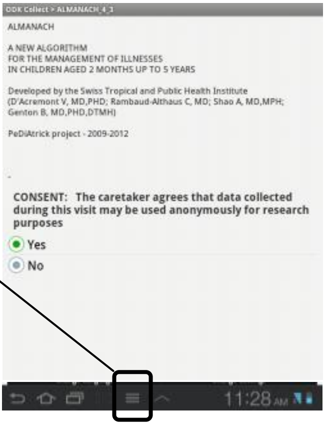

Select ''Change language''

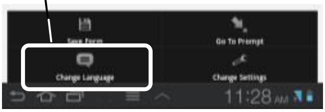

Select ''en'' for English
or ''sw'' for Kiswahili

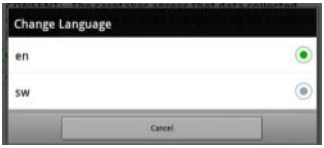

Saving incomplete forms

Example: for laboratory tests:
While the child is sent to the lab, you can **save** the patient form to allow you seeing other patients.
When the child will be back from the lab you will **retrieve** the case and complete the assessment.

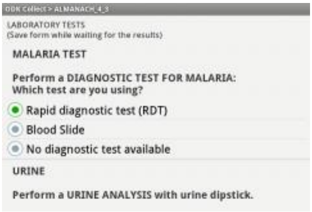

How to save an incomplete form
a- Touch the back arrow to activate saving screen
b- Touch ''save changes'' to save the form while patient is going to the lab.
Note: After saving the form, ALMANACH application will return to the Main Screen Menu *(see p. 2)* ready to begin a new patient.

a

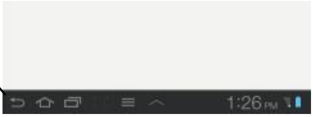

b

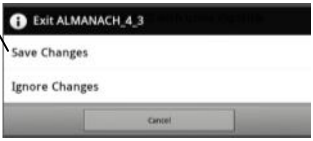

How to retrieve a saved form
c- From main screen menu touch ''Today's patients'' *(see p. 2)*
d- Identify the patient by his ID*(1)*, the time you saved the form*(2)*, the gender of the patient*(3)*, and his/her age in years*(4)*, in the list of today's patients.
e- Touch patient's icon. A new screen comes. Touch ''Continue'' to retrieve the form.
f– The patient's form appears. Swipe right to left until you arrive to the screen for laboratory resuts.

c

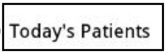

d

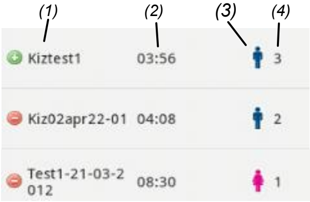

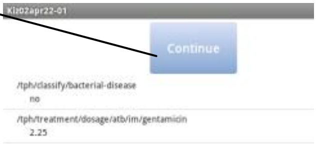

At completion of visit: Save & send data

Saving at completion of visit
Select "Mark Form as Finalized"
Touch "Save Form and Exit".
A message indicates data is saving.
Application returns to main screen.
You can begin a new patient or retrieve a saved case.

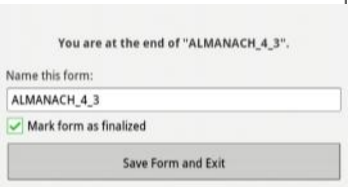

How to send data to the server:
At the end of each working session, you need to send data to the server:

Connect to internet
From the Home page open Settings 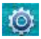

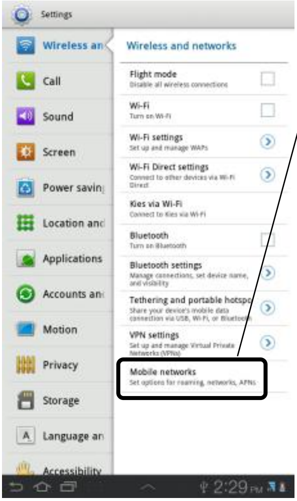

Select ''Mobile Networks''

Then tick ''Use packet data''

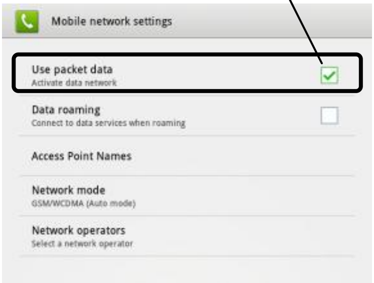

A warning message appears.
Touch ''OK''

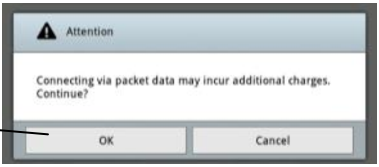

Note: Internet should only be connected while you are sending data. Make sure to disconnect from internet after you are finished uploading your data.

At completion of visit: Save & send - contiued

How to send data to the server - continued

Sending data
Once connected to internet, open ALMANACH

Touch

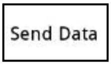

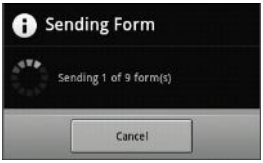

A message appears.
Just wait while data are sent

If the server requires authentication,
touch OK.

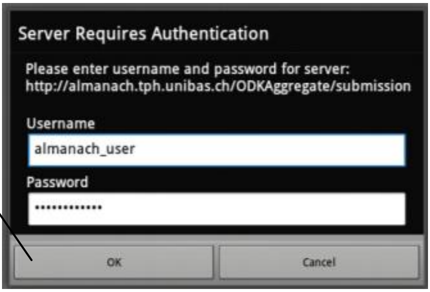

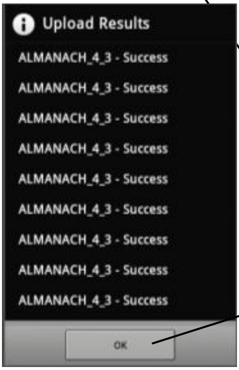

A screen displays the upload results.
Touch OK and you will be back at the main screen menu

Disconnect from the internet:
Go back to the home page. Click “Setting icon”, then click on the “Mobile networks” and deselect “use packet data” box. You are now disconnected from the internet.
